# Estimating the human development impacts and economic returns from an adolescent cash ‘plus’ programme in Kenya: An economic modelling study

**DOI:** 10.1371/journal.pgph.0006822

**Published:** 2026-07-24

**Authors:** William E. Rudgard, Chris Desmond, Broline S. Asuma, Ritapriya Bandyopadhyay, Sopuruchukwu Obiesie, Hernando Grueso, Kathryn Grace Watt, Luciana Leite, Rachel Yates, Brendan Maughan-Brown, Elona Toska, Lucie D. Cluver

**Affiliations:** 1 Department of Social Policy and Intervention, University of Oxford, Oxford, United Kingdom; 2 Centre for Social Science Research, University of Cape Town, Cape Town, South Africa; 3 School of Economics and Finance, University of Witwatersrand, Johannesburg, South Africa; 4 Centre for Research in Health Systems, University of KwaZulu-Natal, Durban, South Africa; 5 Southern Africa Labour and Development Research Unit (SALDRU), University of Cape Town, Cape Town, South Africa; 6 Department of Psychiatry and Mental Health, University of Cape Town, Cape Town, South Africa; African Population and Health Research Center, KENYA

## Abstract

The Government of Kenya plans to scale-up an adolescent-focused cash ‘plus’ programme to improve educational attainment and prevent adolescent pregnancy. We aimed to estimate the programme’s impacts, economic returns, and cost-effectiveness using model-based economic evaluation. We modelled the delivery of conditional cash transfers ‘plus’ adolescent clubs, community conversations, parenting support and psychosocial support to poor adolescents in nine selected counties from 2026 to 2029. Two scenarios were compared: a baseline in which intervention variables remained constant at 2025 levels, and an immediate scale-up sustained throughout 2026–2029. Human development impacts were simulated using an integrated modelling framework. Gains in years of schooling were projected with an education transition model, while reductions in adolescent pregnancy, child marriage, and experiences of violence were estimated with a deterministic age–cohort transition model. Economic returns to schooling were calculated with Mincerian earnings functions. Model parameters were derived from rigorous impact evaluations, nationally representative surveys, and Kenya’s national social registry. The four-year intervention, costing US$46.1 million, is projected to reach 140,000 adolescents, and compared with the baseline, generate an additional 74,900 (+7%) years of schooling; and avert 1,420 (-8%) adolescent pregnancies, 1,500 (-11%) child marriages, 2,970 (-3%) experiences of sexual violence, and 21,800 (-8%) experiences of emotional/physical violence. Furthermore, the intervention is projected to generate US$318 million in discounted lifetime labour earnings, representing a seven-fold return on investment. The estimated cost was US$567 per additional year of schooling, and US$109 when benefits across multiple outcomes were considered, comparing favourably with other adolescent education interventions. Findings were robust to sensitivity analyses, with a >98% probability that returns exceeded programme costs. These findings suggest that scaling up an adolescent focused cash ‘plus’ programme could substantially improve adolescent human development, while delivering strong economic returns and good value for money.

## Background

Adolescents in Kenya account for more than a fifth of the total population [1,2]. Despite considerable progress in adolescent-responsive policies, standards, and guidelines nationally, persistent social and economic constraints, limited access to healthcare, and gender inequities continue to restrict adolescents’ opportunities [3–5]. High school absenteeism and drop-out contributes to early entry into informal, low-paying work, reinforcing cycles of poverty. Adolescent girls face additional risks with HIV acquisition rates six times higher than boys, lower rates of secondary school completion, 23% marrying before age 18, and 15% experiencing adolescent pregnancy [6–8]. Factors like food insecurity, limited parental monitoring, and inequitable gender norms play an important role in increasing these risks [9].

Adolescence is a pivotal developmental window in which investments can yield substantial and lasting returns [10–14]. However, programming has often remained sectorally fragmented focusing on service-delivery interventions, such as contraceptive provision, HIV pre-exposure prophylaxis, maternal healthcare, or education fee waivers [15–18]. Increasingly, social assistance, and particularly cash transfer initiatives, have been recognised as a structural strategy to support adolescent development by alleviating the financial constraints that shape families’ and adolescents’ behaviours [19]. Cash transfers have consistently been shown to improve school enrolment and attendance, reduce child labour, and delay sexual debut [20–27]. Emerging longitudinal evidence further suggests that these benefits may extend into early adulthood through improved labour market outcomes [28].

While education impacts of cash transfers are well established, evidence of effects on adolescent pregnancy, child marriage, and violence victimisation remains mixed [20,29–31]. This has contributed to increasing interest in leveraging cash transfer programmes as platforms for complementary interventions that directly target skills, norms, and behaviours among adolescents and influential reference groups such as caregivers and communities. For example, the Adolescent Girls Initiative Kenya (AGI-K) programme combined conditional cash transfers with safe spaces, financial education, savings activities, and community dialogues to shift community gender norms, reduce adolescent pregnancy and child marriage, and improve adolescent girls’ self-efficacy, savings practices, and financial literacy [32–35]. Similar integrated approaches have demonstrated positive effects on gender attitudes, life skills, and economic participation in other settings across Africa [36–44].

The evidence base has now reached a stage where consideration of scaling is warranted. However, scaling social interventions such as multi-component cash ‘plus’ programmes, requires robust economic evidence to inform decisions about value for money and sustainability. Model-based economic evaluation offers a tool to estimate potential impacts, economic returns, and cost-effectiveness under alternative implementation scenarios [45,46]. Because these programmes generate broad and interrelated effects, economic evaluations must capture a fuller range of potential human development outcomes than traditional cost analyses typically consider [47]. Several relevant outcomes, including reductions in child marriage and adolescent pregnancy, are not readily monetised, and others, such as education, hold intrinsic value in expanding life opportunities and personal development. Economic assessments should therefore incorporate consequential benefits beyond conventional financial metrics, reflecting the full scope of programme impacts. Recent simulations of child-sensitive cash transfers in Malawi, Somalia, Uganda, and Zambia illustrate how modelling can inform investment decisions by estimating both poverty reduction and measurable economic returns [48].

Building on this evidence base, the Government of Kenya developed a national adolescent-focused programme to improve school progression and delay adolescent pregnancy [40,49]. The programme was designed in 2024–25 through a multi-stakeholder technical working group led by the State Department of Social Protection’s Directorate of Child Services in collaboration with development partners. To inform this process, the aim of this study was to conduct a model-based economic evaluation to estimate the projected impact, economic returns, and cost-effectiveness of the proposed programme over a four-year implementation period. The study synthesised evidence from multiple sources to project long-term effects on adolescent education, pregnancy, child marriage, and violence, alongside associated wage-based productivity gains, compared to a counterfactual scenario.

## Methods

### Ethics statement

This study used only secondary, de-identified data and did not involve human participants. Approvals were obtained for the KDHS from The DHS Program (ICF/USAID) and for the VACSs from the Centers for Disease Control and Together for Girls. County-level administrative data were obtained from the Government of Kenya. No ethical approval was required for this study.

### Study design

We conducted a model-based economic evaluation to estimate the costs, human development impacts, and economic returns of scaling an adolescent cash ‘plus’ programme in Kenya between 2026 and 2029. The study was conducted in close consultation with a multistakeholder technical working group comprising government and implementing partners. The group met weekly from June 2024 to March 2025 to review evidence, refine programme design features (target population, intervention mix, transfer level), and validate key modelling assumptions [39].

We report our study in accordance with the Consolidated Health Economics Evaluation Reporting Standard (CHEERS) checklist in see Table A in S1 Appendix [50].

### Study population and setting

The model simulated outcomes for cohorts of adolescent girls and boys aged 10–18 years living in households enrolled in Kenya’s *Inua Jamii* National Safety Net Programme (NSNP), reflecting the target population for the planned cash ‘plus’ programme. The upper age range reflects the Directorate of Child Services’ mandate, which extends to age 18. The NSNP provides targeted assistance via four programmes: the Cash Transfer for Orphans & Vulnerable Children (CT-OVC), the Hunger Safety Net Programme for drought-affected households, the Older Persons Cash Transfer, and the Persons with Severe Disabilities Cash Transfer. In 2023, these programmes collectively reached over 1.3 million households nationwide [51].

The intervention was modelled in 18 sub-counties across nine counties: Bungoma, Homa Bay, Kilifi, Mandera, Marsabit, Migori, Nairobi, Samburu, and Wajir. The counties were selected through group consensus by the technical working group based on high poverty levels; elevated adolescent out-of-school and pregnancy rates; existing social protection programming; and regional representation. The number of sub-counties was determined by the available budget of approximately United States dollars (US$) 40 million. A preliminary top-down costing exercise using administrative data from the NSNP Management Information System (MIS) data indicated that this budget would allow coverage of all adolescents in NSNP households across 18 sub-counties.

### Comparators

To estimate the impacts and economic returns from the cash ‘plus’ programme, we compared two modelling scenarios: a baseline scenario, and an intervention scenario. In the baseline scenario, age-specific incidence rates (health outcomes) and grade-specific transition rates (education outcomes) were held constant at their estimated level in 2025 for the period from 2026 to 2029, reflecting a continuation of the current situation without additional investments. In the intervention scenario, the cash ‘plus’ programme was scaled up and then maintained at that level until 2029. Intervention effects were applied as relative changes to baseline parameters: age-specific incidence rates were reduced according to estimated relative risks, and grade-specific transition probabilities were increased to reflect improvements in school entry and retention. Immediate scale up was assumed feasible given the Government of Kenya’s ability to leverage existing registries of eligible households to target new beneficiaries. The modelled cash ‘plus’ intervention comprised five components:

#### Cash transfers conditioned on school attendance –.

Kenyan shillings (KSh) 1,000 monthly for nine school months, paid to caregivers in addition to existing social assistance. Transfers were conditional on regular school attendance, monitored by teachers. Prolonged non-attendance would lead to the suspension of payments.

#### Adolescent clubs –.

Weekly (girls) and bi-weekly (boys) life-skills clubs addressing reproductive health, gender equality, and personal development

#### Community conversations –.

Quarterly facilitated community conversations engaging caregivers and community leaders to discuss issues affecting adolescents, including education, gender norms, and child protection.

#### Parenting support –.

A 14-session intervention based on the World Health Organization’s and UNICEF’s Parenting for Lifelong Health Teen (PLH Teen) programme, designed to prevent violence by strengthening positive caregiver-adolescent relationships [52].

#### Psychosocial support and referrals –.

A structured case management process delivered through the Directorate of Children Services.

### Study outcomes

We modelled six outcomes with evidence-supported intervention effects of the proposed cash ‘plus’ intervention. They included: years of schooling completed; adolescent pregnancies averted; child marriages averted; experiences of emotional and physical violence averted; and experiences of sexual violence averted. Child marriage was defined as marriage or union before the age of 18, and adolescent pregnancy was defined as pregnancy before the age of 20 [53,54]. Violence outcomes were defined using past 12-months measures: emotional and physical violence perpetrated by parents, adult caregivers, or other adult relatives; and sexual violence (unwanted sexual touching, attempted sex, forced sex, or pressured sex) perpetrated by any individual. We also estimated the expected wage returns from additional years of schooling. All impacts were measured in natural units.

### Model structure

We developed deterministic simulation models in Microsoft Excel to estimate the projected impacts of the adolescent cash ‘plus’ programme. Education impacts were modelled using a grade-structured cohort transition model based on the UNESCO Educational Simulation Model (ESM) [55,56]. This model tracks multiple school-grade cohorts over annual time steps. Its baseline state is defined by age- and grade-specific enrolment rates, and its evolution is determined by grade-specific progression rates, with students able to advance by a maximum of one grade per year.

Health and protection outcomes (adolescent pregnancy, child marriage, and violence victimisation) were modelled using age-structured cohort models based on Lexis modelling principles [57]. These models simulate multiple age cohorts advancing in annual time steps, with each cohort ageing one year per cycle. Cohorts enter at programme-eligible ages (10–18 years) and are followed over time as they age through the relevant risk periods (e.g., up to age 20 for adolescent pregnancy). Incidence of pregnancy, marriage, and violence was determined by age-specific incidence rates [58].

The full model framework, including the education and health transition models, analytical code used to derive effect estimates and parameters, and supporting documentation, is available via the Open Science Framework (OSF) [59].

**Fig 1:**
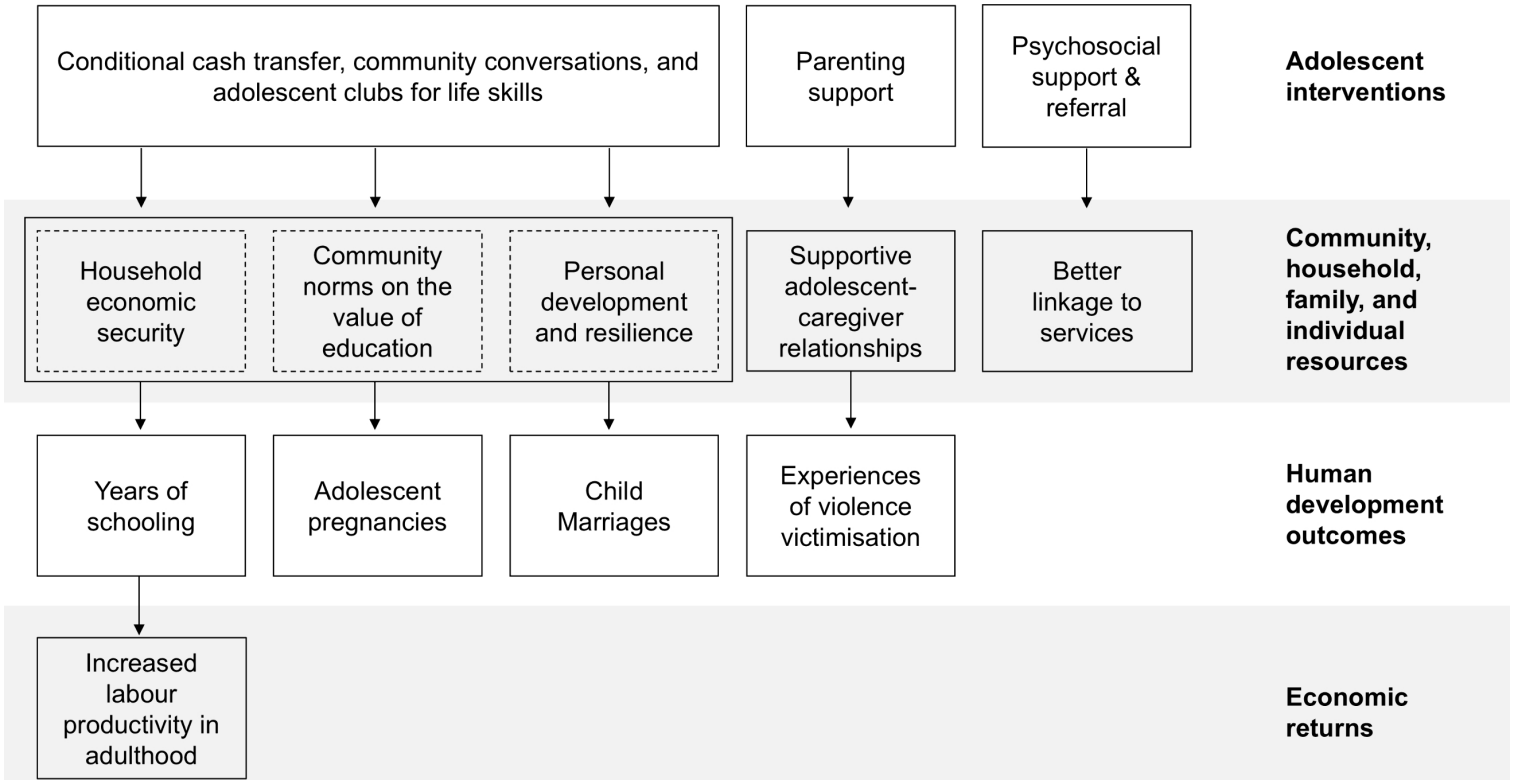
Measurement framework for modelling the impacts of selected interventions on adolescent health and wellbeing, as well as their economic benefits.

Modelling was necessary as the adolescent cash ‘plus’ programme has not been implemented and real-world data on costs and effects are not yet available. A schematic representation of the measurement framework and core evidence-based pathways for how the programme influenced different outcomes is presented in Fig 1. Further details on model structure, data sources, and assumptions are provided in the sections below and in Text A-Text D in S1 Appendix.

### Data sources and parameterisation

Models were parameterised using published literature; publicly available, nationally representative survey data; administrative data from the NSNP’s MIS; and expert consultation.

#### Population parameters*.*

Baseline demographic and outcome parameters were drawn from the 2022 Kenya Demographic and Health Survey (KDHS), and the 2019 Kenya Violence Against Children and Youth Survey (VACS) [56,60]. KDHS provided parameters for schooling, marriage, and pregnancy, while VACS provided parameters for violence outcomes (emotional, physical, and sexual). To reflect programme targeting to NSNP households, KDHS-derived parameters were restricted to the lowest wealth quintile and drawn from the nine priority counties. A similar restriction was not feasible for VACS due to unstable estimates and its national-level representativeness. Therefore, violence victimisation estimates were derived from national-level data [56,60]. Full parameter inputs are summarised in Text E and Text F in S1 Appendix.

#### Intervention effects.

Effectiveness estimates were identified through targeted literature reviews and expert consultation, Table 1, Text G in S1 Appendix and Text H in S1 Appendix. Where available, Kenya-specific evidence was used to parameterise intervention effects; when such evidence was unavailable, we prioritised evidence from geographically and contextually similar sub-Saharan African settings before considering broader regional evidence or assumptions.

**Table 1:** Summary of estimates of intervention effectiveness used to parameterise our measurement framework.

	RR	LCI-UCI	Reference
**Conditional cash transfers, community conversations, and adolescent clubs**			
Effect on return to school in year 1	1.07	1.01-1.14	[32,62]
Effect on primary school retention	1.02	0.99-1.06	[66]
Effect on secondary school retention	1.08	1.03-1.12	[66]
Effect on adolescent pregnancy	0.83	0.65-1.07	[24,33,34]
Effect on child marriage	0.80	0.57-1.12	[24,33]
**Parenting support**			
Effect on emotional violence	0.55	0.54-0.56	[40,52]
Effect on physical violence	0.56	0.55-0.58	[40,52]
Effect on sexual violence	0.79	0.63-0.95	[64]

Abbreviations: RR, Relative risk; LCI, Lower 95% confidence interval; UCI, Upper 95% confidence interval.

Notes: These effect estimates reflect impacts observed during or shortly after programme implementation; post-exit persistence is modelled separately. Effect estimates are derived from experimental studies, except for sexual violence, which is based on observational evidence and may be subject to greater uncertainty. Where multiple studies are referenced, estimates were derived using meta-analysis to pool effect sizes. Effects on adolescent pregnancy and child marriage are not statistically significant (confidence intervals include the null) and should be interpreted with caution.

For the combined conditional cash transfers, community conversations and adolescent clubs, we modelled three education-related effects: i) increased school entry or return in the first year of receipt; ii) increased primary retention; and iii) increased secondary retention. The school return effect was derived from AGI-K and CT-OVC evaluation findings, synthesised through meta-analysis of data from the AGI-K sites in Nairobi and Wajir, and from the multi-district CT-OVC evaluation, to enhance generalisability across distinct contexts [33,34,61]. Retention effects were drawn from the evaluation of the CT-OVC programme [62]. Effects on adolescent pregnancy and child marriage were also derived from AGI-K and CT-OVC evaluation findings [24,33–35].

Parenting support effects on emotional and physical violence victimisation were derived from randomised controlled trials of the Sinovuyo Teen and Furaha Teen interventions conducted in South Africa and Tanzania, respectively, as no comparable Kenyan trial evidence was available at the time of modelling [52,63]. As trials were not powered to detect effects on sexual violence, this parameter was informed by observational evidence [64].

Post-exit effect persistence was modelled as intervention specific. For the combined conditional cash transfers, community conversations, and adolescent clubs intervention, effects on education, pregnancy, and marriage outcomes were assumed to persist for two years following programme exit (whether due to graduation or programme completion). This assumption is consistent with evidence of sustained impacts on education outcomes observed at 48 months post-baseline (approximately two years post-exit) in the AGI-K evaluation, and is broadly supported by evidence from the CT-OVC evaluation, which shows positive but not consistently significant effects on education outcomes at similar follow-up periods [33,34,61]. Impacts on pregnancy and marriage appear to be more context-specific and are primarily observed in the more disadvantaged Wajir setting, compared to Nairobi and the CT-OVC evaluation districts. Given limited evidence to support different persistence periods by outcome, we apply a common duration across outcomes for simplicity. To reflect uncertainty in the persistence of effects, sensitivity analyses were conducted varying the duration of persistence from one to four years post-exit, with four years representing an upper-bound scenario informed by longer-term follow-up evidence from AGI-K in Wajir [35].

For violence outcomes, under the parenting support intervention, we modelled post-exit attenuation using an annual 33% decay in effect size based on long-term follow-up evidence from the Philippines [65].

The model had to account for the joint delivery of five intervention components. Effectiveness estimates for conditional cash transfers, community conversations, and adolescent clubs were drawn from the AGI-K evaluation, which assessed these components jointly and therefore incorporated potential synergistic effects [32–35]. In the absence of robust evidence on cross-component interactions, parenting support effects were assumed to be additive to the cash ‘plus’ platform [38]. The effects of violence case management were not modelled due to insufficient evidence on its effectiveness for reducing violence incidence. However, costs were included to reflect the resources required for ethical programme implementation.

#### Intervention costs.

Programme costs were estimated using an ingredients-based approach informed by the Abdul Latif Jameel Poverty Action Lab (J-PAL) costing template [67]. Cost inputs included transfer payments, personnel, training, supervision, and implementation costs for each intervention component. Administrative costs were estimated at the sub-county level and assumed to scale proportionally across sub-counties. Cash transfer values were determined by the technical working group following a review of existing evidence [68]. Additional costs were included for monitoring compliance with school attendance conditionality by sub-county education officers. Unit costs for adolescent clubs and community conversations were drawn from cost data provided by the AGI-K evaluation team, based on their assessment of scaled implementation in Wajir County, Kenya [49]. Unit costs for parenting support were based on implementation data from PLH Teen programmes in South Africa, Tanzania, and Thailand, shared by the Global Parenting Initiative [38,52]. A full table of unit costs, including ranges and data sources, is available via the OSF repository [59].

As the intervention was expected to increase school enrolment and retention, we also estimated the cost of additional teachers needed to maintain current pupil–teacher ratios in primary and secondary schools. The marginal teacher cost per additional student-year was calculated by dividing 2023 teacher salaries by current pupil–teacher ratios [69,70].

### Lifetime earnings returns from education

We estimated lifetime earning gains using a Mincerian earnings framework. Following Perumal et al. (2022), baseline average wages were assumed to equal two-thirds of Kenya’s 2023 Gross Domestic Product per capita (current US$) [71]. Each additional year of schooling was associated with an 11% increase in earnings, based on regional estimates for Africa from Patrinos and Psacharopoulos (2018) [72].

We assumed a six-year lag between programme receipt and entry into the labour market, reflecting the expected time required to complete schooling. Earnings were projected over a 40-year working life. Real wages were assumed to grow at 2% annually, consistent with Perumal et al. (2022) [71].

### Perspective, time horizon, discounting, and currency conversion

Costs were estimated from a provider perspective, while outcomes and productivity gains were assessed from a beneficiary perspective. Return on investment (ROI) is therefore interpreted as the ratio of monetised beneficiary benefits to programme costs, reflecting a partial societal perspective that does not capture broader social welfare effects such as wider economic impacts or distributional considerations.

Programme implementation costs were modelled over a four-year horizon (2026–2029), reflecting the planned duration of implementation. Consistent with WHO-CHOICE guidelines, long-term health and lifetime earnings effects were projected beyond the programme period to capture their full lifetime impact.

Costs and lifetime earnings gains were discounted at 3% annually to estimate present values at the start of the analytic period (2026) [73]. All costs were adjusted to 2023 price levels in local currency and converted to US dollars using the 2023 average exchange rate of US$ 1 = KSh 134.7 [74,75].

### Sensitivity analysis

We conducted a probabilistic sensitivity analysis (PSA) with 20,000 Monte Carlo draws [58]. Parameter uncertainty was sampled from specified distributions (truncated normal, lognormal, triangular, uniform), Table 2. Present value estimates of lifetime earnings gains were calculated for each draw.

**Table 2:** Model parameters, base-case values, ranges, and probability distributions used in probabilistic sensitivity analysis.

Parameter	Base	Low; High	PSA distribution
Total years of schooling gained	74,900	62,700; 87,200	Truncated Normal (mean = 74,900, SD ≈ 6,250, lower = 62,700, upper = 87,200)
Mincer return per year	11%	5.5%; 16.5%	Truncated Normal(mean = 0.11, SD = 0.03, lower = 0)
Real wage growth (annual)	2%	0%; 4%	Triangular (min = 0%, mode = 2%, max = 4%)
Baseline wage level (annual)	KSh 17,580	14,064; 21,096	Lognormal (mean = 17,580, CV ≈ 0.10) truncated at >0
Discount rate	3%	0%; 5%	Triangular (min = 0%, mode = 3%, max = 5%)
Female:male return ratio	1.00	1.0; 1.3	Truncated Normal (mean = 1.00, SD = 0.08, lower = 1.0, upper = 2.0)
Female realised earnings fraction	1.00	0.6; 1.0	Uniform (0.6,1.0)

Abbreviations: KSh, Kenyan Shillings; CV, Coefficient of variation, SD, Standard deviation; PSA, Probabilistic sensitivity analysis.

Notes: For years of schooling gained, low and high values represent scenario bounds informed by uncertainty in the programme’s estimated effect. In the PSA, total schooling gains are drawn from a truncated normal distribution bounded by these scenario values. For baseline wage, low and high values reflect a ± 20% assumed range around the central estimate to capture uncertainty in wage levels.

We also performed one-way deterministic sensitivity analyses, varying individual parameters across their respective uncertainty ranges while holding others constant. Results were summarised using a tornado diagram.

In addition, we explored structural uncertainty related to the persistence of effects from cash ‘plus’ programming by modelling alternative durations of effect sustainability, informed by evidence suggesting impacts are typically measured up to two years post-receipt, with some evidence of persistence up to four years [24,33–35,61,62,76].

### Cost-effectiveness analysis

We estimated traditional cost-effectiveness ratios (CERs) by dividing total programme costs by total outcome units achieved.

To address the multi-outcome nature of the intervention, we also applied a discounted CER allocation method outlined in Grueso et al. (2024), proportionally allocating programme costs across outcomes based on their contribution to total benefits [77]. These proportional weights represent cost shares rather than welfare weights.

CERs were compared with published estimates from other education-focused programmes in Kenya [78]. Because these comparator estimates were derived using different costing methods, perspectives, time horizons, and contexts, comparisons should be interpreted as indicative rather than directly equivalent.

## Results

Population estimates for Kenya and the 18 sub-counties targeted by the cash ‘plus’ programme are summarised in Table 3. We estimate that 689,000 adolescents live in these sub-counties, of whom 108,000 live in households enrolled in the NSNP and would therefore be eligible for the programme in Year 1. Applying an annual single-age cohort growth rate of 2% to reflect demographic change and cohort replacement, we project that up to 152,000 adolescents in NSNP households would be eligible for the cash ‘plus’ programme over the four-year implementation period.

**Table 3:** Summary of adolescent population estimates and the expected number of beneficiaries for each intervention, using 2023/24 data.

	Estimate	Assumption	Ref
**Kenya**			
GDP 2023 (current US$, billions)	108		[79]
Total population	51,526,000		[80]
Adolescents	10,399,000		[80]
Girls	5,240,000	Girls = 50.4%	[80]
Adolescents living below the poverty line	3,432,000	National poverty rate = 34%	[81]
Girls	1,729,000	National poverty rate = 34%	[81]
**Target sub-counties** ^**1**^			
Adolescents	689,000		[80]
Adolescents living in NSNP households in the first year	108,000		[82]
Girls	54,000	Girls = 50.4%	[80]
Adolescent single age growth rate	2%		[83]
Adolescent school attendance rate in poorest wealth quintile	85%	Uniform rates across all sub-counties within counties	[56]
Girls	84%		[56]
Boys	86%		[56]
Adolescent pregnancy incidence rate (ages 10–19), in poorest wealth quintile (per 100 woman-years)^2^	4.63		[47]
Girl child marriage incidence rate (ages 10–17), in poorest wealth quintile (per 100 woman-years)^2^	3.46		[47]
Emotional violence (past 12 months) among young people (aged 13–21 years)		Uniform rates nationally	[60]
Girls and young women	11%		[60]
Boys and young men	6%		[60]
Physical violence (past 12 months) among young people (aged 13–21 years)			[60]
Girls and young women	13%		[60]
Boys and young men	13%		[60]
Sexual violence (past 12 months) among girls and young women (aged 13–21 years)	15%		[60]
Uptake of cash ‘plus’ programme	91%	A 7% increase in school attendance linked to the cash ‘plus’ programme	[49]

Abbreviations: Ref, Reference; NSNP, National Safety Net Programme; MIS, Management Information System.

Notes: All estimates are rounded to three significant figures. Unless stated otherwise, adolescent refers to the age group 10–18 years. Pregnancy and marriage estimates are derived from age-specific incidence rates from KDHS 2022, aggregated to the reported age bands. Violence estimates are based on past 12-month prevalence from VACS 2019. KDHS estimates are restricted to the poorest wealth quintile in priority counties, while VACS estimates are national due to sample size constraints. ^1^Priority counties include Bungoma, Homa Bay, Kilifi, Mandera, Marsabit, Migori, Nairobi, Samburu, and Wajir. ^2^Single-year age-specific incidence rates underlying these aggregated estimates are reported in the supplementary material.

Baseline school attendance among adolescents aged 10–18 years in the target sub-counties is estimated to be at 85%, with approximately equal rates for girls and boys. Among adolescent girls, 16% report having ever been pregnant and 3% report having entered child marriage. Twelve-month prevalence of violence victimisation is estimated at 17% for emotional violence (23% among girls and 12% among boys), 29% for physical violence (30% among girls and 29% among boys), and 15% for sexual violence among girls.

Applying the AGI-K relative effect estimate (RR = 1.07) to baseline attendance increases attendance from 85% to approximately 91% in the first year of implementation. Among the 108,000 adolescents eligible for the cash transfer component in the first year, this corresponds to approximately 99,400 receiving it. The remaining 9% of adolescents not meeting the attendance requirement reflects structural barriers to school participation, including pregnancy and early motherhood among adolescent girls. Accounting for annual cohort growth and programme turnover, we estimate that approximately 140,000 unique adolescents would receive the cash transfer component of the programme over the full four-year period.

### Estimated intervention costs

A summary of cost components and total costs is summarised in Table 4. We estimate that scaling up the cash ‘plus’ programme over four years would cost a total net present value of US$36.6 million, equivalent to an average of US$9.2 million per year over the implementation period. Additional schooling costs required to maintain existing pupil–teacher ratios in response to increased enrolment were estimated at US$9.5 million. These costs are incurred over a longer time horizon than programme delivery, extending to 2043, and are therefore reflected across multiple future years in the net present value estimates. Combining programme and education system costs, the total net present value cost was US$46.1 million. Relative to approximately 140,000 adolescents reached over the four-year implementation period, this corresponds to an average cost of US$304 per beneficiary.

**Table 4:** Estimated net present value (NPV) of programme and education system costs (US$).

Component	Cost (US$)	% of total
Administration	935,000	2%
Conditional cash transfers	28,200,000	61%
Cash transfers	27,100,000	
School attendance monitoring for conditionality	1,110,000	
Community conversations, adolescent clubs, parenting support	5,470,000	12%
Material development	23,600	
Staff training	2,220,000	
Beneficiary training	3,230,000	
Psychosocial support and referral	1,980,000	4%
Staff training	496,000	
Implementation	1,490,000	
**Programme subtotal**	**36,600,000**	**80%**
Additional teacher salary costs	9,500,000	20%
**Combined total**	**46,100,000**	**100%**

Abbreviations: US, United States.

Notes: All estimates are in US$ 2023 and rounded to three significant figures. Costs are presented as net present values (NPV), discounted at 3% per annum. Estimates reflect a provider perspective and include programme and education system costs associated with increased school participation. Conditional cash transfers include transfer payments and delivery/monitoring costs. Additional teacher salary costs reflect maintaining pupil–teacher ratios. Household costs are not included.

The largest cost driver was the conditional cash transfer component, at US$28.2 million, accounting for approximately 61% of total costs. Community-based components (adolescent clubs and community conversations) were estimated to cost US$5.47 million (12%). Psychosocial support and referrals accounted for US$1.98 million (4%), and administrative costs totalled US$935,000 (2%).

### Estimated intervention impacts on human development outcomes and lifetime earnings

Estimated cumulative impacts of the adolescent cash ‘plus’ programme are summarised in Table 5. Under the intervention scenario, compared with the unchanged policy scenario, we estimate that the programme would generate 74,900 additional years of schooling (+7% relative to baseline); avert 1,500 child marriages (-11%) and 1,420 adolescent pregnancies (-8%); and prevent 2,970 experiences of sexual violence (-3%) and 21,800 experiences of emotional or physical violence (-8%).

**Table 5:** Estimated impacts of cash ‘plus’ programme on beneficiary adolescents and associated economic returns.

	Overall		Girls		Boys	
	Absolute change (Lo-Hi)	Percentage change (Lo-Hi)	Absolute change (Lo-Hi)	Percentage change (Lo-Hi)	Absolute change (Lo-Hi)	Percentage change (Lo-Hi)
Years of schooling gained	74,900 (62,700-87,200)	7 (6–8)	36,100 (30,000-42,100)	7 (5–8)	38,800 (32,600-45,100)	8 (7–9)
Adolescent pregnancies averted	1,420 (868-2,920)	8 (3–17)	1,420 (868-2,920)	8 (3–17)	–	–
Child marriages averted	1,500 (165-3,300)	11 (1-24)	1,500 (165-3,300)	11 (1-24)	–	–
Experiences of emotional & physical violence averted	21,800 (21,700-22,000)	8 (8–8)	12,400 (12,000-12,900)	8 (8–9)	9,410 (9,040-9,770)	8 (8–9)
Experiences of sexual violence averted	2,970 (707-5,230)	3 (1–6)	3,910 (889-6940)	3 (1–6)	–	–
Labour earnings gained from increased schooling, US$ millions	318 (265-430)	11 (11–11)	153 (127-179)	11 (11–11)	165 (138-251)	11 (11–11)

Abbreviations: US, United States; Lo, Low; Hi, High.

Notes: All estimates are rounded to three significant figures. Low and high estimates are derived from simulations using the 95% confidence intervals of pooled effect sizes from meta-analyses of relevant interventions. Percentage changes are relative to baseline levels in the target population. Estimates assume that effects of the cash ‘plu’ component persist for 2 years after cessation of transfers. Parenting-related effects are assumed to decay by 33% per year over this period; alternative scenarios are presented in the supplementary material.

Estimates of labour earnings gains are calculated using baseline parameter values for the labour returns model (including returns to schooling, wage growth, discount rate, and baseline wages). Low and high estimates for economic outcomes reflect uncertainty in intervention effects, while uncertainty in economic parameters and schooling gains is explored separately through probabilistic and deterministic sensitivity analyses.

Lifetime economic returns were estimated by applying an assumed Mincerian return to each additional year of schooling to projected baseline earnings, with returns accruing over a 40-year working life and discounted to present value. The projected economic return from increased schooling is estimated at US$318 million in present value terms. High- and low-range estimates are reported in Table 5.

**Fig 2:**
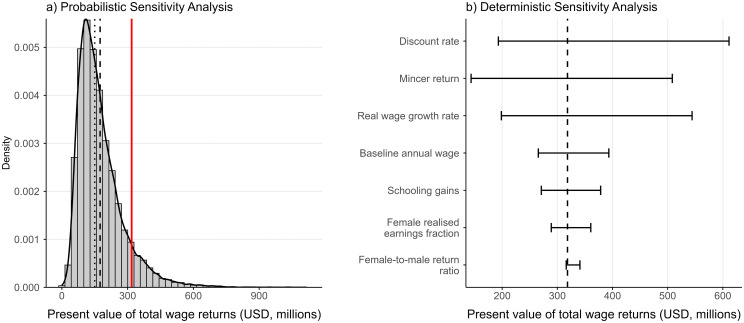
Sensitivity analysis of total incremental present value of lifetime earnings returns. Panel (a) shows the distribution of total lifetime earnings returns from probabilistic sensitivity analysis (20,000 Monte Carlo simulations). The deterministic estimate is shown in red; dashed and dotted lines indicate the simulated mean and median. Panel (b) presents a deterministic one-way sensitivity (tornado) analysis, illustrating the relative influence of parameter uncertainty on total present value, with the dashed vertical line representing the baseline estimate.

In probabilistic sensitivity analysis, the mean present value of total wage returns was US$174 million (SD = US$104 million), with a median of US$150 million, Fig 2. The 95% uncertainty interval ranged from US$49 million to US$441 million. The probability that the total incremental present value exceeded the programme cost of US$46.2 million was 0.98, while the probability of exceeding twice the programme cost was 0.81. Deterministic one-way sensitivity analysis indicated that results were most sensitive to assumptions about the discount rate, mincer return, and real wage growth with comparatively smaller influence from other parameters, including baseline wage levels, schooling gains, and gender-related parameters, Fig 2.

Additional sensitivity analyses explored assumptions regarding the persistence of intervention effects over time. While much of the evidence base for cash transfer programmes reflects impacts measured up to approximately two years post-receipt, longer-term follow-up from AGI-K in Wajir suggests that some effects may persist for up to four years [35]. Under alternative scenarios in which impacts attenuate more rapidly, estimated lifetime earnings returns were reduced but remained positive and exceeded programme costs. Conversely, assuming sustained effects over a longer duration increased projected returns. Overall, conclusions regarding the economic viability of the programme were robust to plausible variations in effect persistence, see Text I in S1 Appendix.

### Estimated intervention cost-effectiveness

Estimates of cost-effectiveness for the modelled cash ‘plus’ programme, alongside comparator interventions identified through literature review and expert consultation are summarised in Table 6.

**Table 6:** Cost-effectiveness of the cash ‘plus’ programme across education, pregnancy, marriage, and violence outcomes, and comparator interventions in Kenya.

Intervention	Follow-up period	Years of schooling gained		Adolescent pregnancies averted		Child marriages averted		Emotional & physical violence averted		Sexual violence averted	
		CER	Discounted CER	CER	Discounted CER	CER	Discounted CER	CER	Discounted CER	CER	Discounted CER
**Cash ‘plus’ programme**	Modelled (lifetime)	567 (487-678)	109 (43-114)	29,900 (14,600-49,000)	4,370 (1,460-4,490)	28,300 (12,800-258,000)	3,660 (1,060-83,000)	1,950 (1,930-1,960)	182 (89-449)	14,300 (8,120-60,100)	6,280 (2,850-30,300)
**Paying for school uniforms** ^**1**^	2-3 years	1,050 (598-4,320)	359 (116-6070)	1,950 (1,010-9,670)	629 (195-15,500)	2,020 (1,150-8,220)	679 (221-11,200)	–	–	–	–
**Girls Merit Scholarship** ^**2**^	1-2 years	857 (561-1810)	857 (561-1810)	–	–	–	–	–	–	–	–
**AGI-K Testing at Scale - Package 3** ^**3**^	1 year	101 (67-203)	9 (8-∞)	622 (276-∞)	336 (130-∞)	430 (244-2,891)	160 (101-∞)	–	–	–	–
**Teacher incentives** ^**4**^	2 years	–	–	–	–	–	–	–	–	–	–

Abbreviations: CER, Cost-effectiveness ratio; AGI-K, Adolescent Girls Initiative-Kenya; US, United States; Lo, Low; Hi, High.

Notes: All estimates are in 2023 US$ and rounded to three significant figures. Low and high estimates are derived from effect sizes corresponding to the lower and upper bounds of the 95% confidence intervals reported in the original published evaluations.

Follow-up periods vary across comparator studies and are not directly comparable to the modelled lifetime horizon used for the cash ‘plus’ programme; comparisons should therefore be interpreted as illustrative. Cost-effectiveness estimates for school uniforms and merit scholarships were drawn from the J-PAL comparative cost-effectiveness analysis (Dhaliwal et al., 2013). The merit scholarship results are based on the Busia district sample and were not statistically significant in the full study sample.

For AGI-K Testing at Scale, confidence intervals cross zero for some outcomes, resulting in unbounded upper estimates for CERs (denoted by ∞). In these cases, ICERs are not well-defined, and no claims of dominance or extended dominance are made. Comparators were selected based on relevance to the Kenyan context and availability of comparable outcome measures.

^1^Duflo et al., 2015; ^2^Kremer et al., 2009; ^3^Austrian et al., 2024; ^4^Glewwe et al., 2003.

For years of schooling gained, the undiscounted CER for the adolescent cash ‘plus’ programme is US$567 per year of schooling. When programmes costs were allocated across all outcomes using the discounted multi-outcome approach, the CER decreased to US$109 per year of schooling. For adolescent pregnancy, the CER was US$29,900 per pregnancy averted (US$4,370 after discounted cost allocation), and for child marriage, the CER was US$28,300 per child marriage averted (US$1,660 after discounted cost allocation).

Compared with published estimates from comparator interventions, the cost-effectiveness of the modelled programme is broadly similar to that of school-based interventions such as uniforms and merit scholarships, and less favourable than point estimates reported for AGI-K Testing at Scale in Wajir (CER of US$101 per year of schooling gained, US$9 after discounted cost allocation) [49,84,85]. However, these comparisons should be interpreted cautiously given differences in study populations, follow-up periods, outcome definitions, and analytical approaches.

Comparisons for adolescent pregnancy and child marriage are less directly comparable, as several comparator interventions were evaluated among girls only, whereas the modelled programme targets both girls and boys. As such, the programme does not appear more cost-effective than comparator interventions for these outcomes, although differences in target populations and study design limit direct comparison. No comparator programmes reported impacts on violence outcomes, precluding comparison for these outcomes.

## Discussion

This modelling study indicates that a US$46.1 million investment in an adolescent cash ‘plus’ intervention across nine prioritised Kenyan counties, implemented over four years, would reach 145,000 adolescents and generate substantial impacts across five adolescent wellbeing outcomes. Compared to a baseline scenario, the intervention is expected to generate gains in years of schooling among adolescents, while also reducing adolescent pregnancies, child marriages, and experiences of sexual, physical, and emotional violence. Furthermore, through increases in years of schooling, the intervention is projected to generate US$318 million in future labour productivity, representing close to a seven-fold return on investment.

The study’s findings indicate significant human development impacts for adolescents, consistent with existing evidence on the positive effects of cash transfers in improving educational attendance and delaying adolescent pregnancy, and the potential of cash ‘plus’ programmes to prevent child marriage [24,32–35]. Although much of the evidence to date has focused on early childhood as a key window for shaping long-term outcomes, adolescence remains a critical and often under-addressed stage of development between childhood and adulthood, during which substantial human development gains can still be realised [86–88]. This is particularly important in Kenya, where adolescents make up a large and growing share of the population, and where investments in their health, education, and future potential can yield considerable social and economic returns [89].

Building on the well-established relationship between years of schooling and improved labour market outcomes, this study estimates that the adolescent cash ‘plus’ programme could generate long-term economic returns through increased adult labour productivity [72]. These findings are consistent with prior research in Kenya showing that secondary education contributes to improved labour market outcomes and reductions in adolescent pregnancy [90]. Although not explicitly modelled in our analysis, education may also support entry into both formal employment and entrepreneurial activities by building life skills, digital and financial literacy, and transferable skills such as problem-solving.

Our estimates depend on several key assumptions, particularly regarding the persistence of programme effects and the projection of long-term earnings. While much of the evidence on cash transfer and cash ‘plus’ programming reflects impacts measured within approximately two years, emerging evidence suggests that some effects may persist for longer periods [33,34,61,76]. Sensitivity analyses undertaken in this study showed that, although the magnitude of estimated returns varied with assumptions about impact, the overall conclusion that the programme yields positive economic benefits remains unchanged.

Estimates of lifetime earnings gains are also sensitive to assumptions about real wage growth and inflation, which can be highly variable in Kenya given fluctuations in economic growth, labour market conditions, and price stability. Incorporating uncertainty in programme effectiveness, particularly in the number of additional years of schooling generated, further contributes to variation in projected returns. These factors influence the present value of benefits and reduce the likelihood of extreme high-return scenarios. Nevertheless, even under conservative assumptions regarding wage growth and discounting, the probability that benefits exceed programme costs remains very high, and the probability of exceeding twice programme costs is also substantial. This supports the robustness of the economic case for investment, while highlighting that the magnitude of returns depends on both macroeconomic conditions and programme effectiveness.

In addition to estimating human development impacts and economic returns, we also estimated the cost-effectiveness of the programme relative to comparator programmes focused on adolescent education outcomes in Kenya. The modelled programme demonstrated favourable cost-effectiveness in improving educational outcomes, with cost-effectiveness broadly comparable to school-based interventions such as uniforms and merit scholarships. For adolescent pregnancy and child marriage, the programme appeared less cost-effective than some comparator interventions. However, these comparisons should be interpreted cautiously given differences in study populations, follow-up periods, outcome definitions, and analytical approaches. Higher cost-effectiveness ratios for these outcomes likely reflect the planned programme’s focus on both girls and boys, whereas some of the comparator interventions targeted only girls. Additionally, the lower cost-effectiveness on child marriage may reflect our national-level modelling, which includes areas with lower baseline prevalence than Wajir, the high-prevalence setting of the AGI-K programme [49].

To our knowledge, this is the first study to assess the impact of scaling up a package of evidence-based, policy-focused interventions on multiple outcomes across the domains of education, health, violence, and labour. By moving beyond single cost–benefit metrics and quantifying impacts across multiple outcome pathways, the study provides a more comprehensive assessment of value across sectors. This is particularly important for facilitating stakeholder engagement and policy development in cross-sectoral programmes, where benefits may otherwise be difficult to compare or attribute [91,92].

In addition, by grounding the model in Kenyan subnational survey data and tailoring key parameters to the Kenyan context wherever possible, we avoid generalising cost-benefit ratios from interventions conducted in very different settings. Given that the absolute impacts and returns of interventions depend heavily on local conditions, this supports more targeted planning in high-need areas and may improve the efficiency and relevance of investment decisions [93,94].

While our study applies rigorous methods, several limitations should also be considered. The model underestimates the benefits accruing to adolescents, as modelled interventions are likely to have additional impacts on adolescents’ lives beyond the outcomes evaluated. For example, there is evidence from Kenya that cash transfers are associated with improved adolescent mental health, but limited longitudinal data prevented us from modelling these effects over time [95]. Similarly, impacts on adolescents’ agency, such as increased voice, choice, and control, are difficult to quantify. Future research should explore incorporating these outcomes into economic models [96].

The study’s reliance on secondary data and modelling meant that private costs borne by participants and their households could not be directly captured. These costs are therefore excluded, and results reflect a provider perspective. Their net effect is likely to be mixed, with increased school participation raising short-term household expenditures and reductions in adolescent pregnancy and early marriage lowering costs. As a result, estimated returns may be over- or understated depending on the balance of these effects.

Potential impacts on education quality are also not fully captured. Although we incorporated the additional costs required to maintain pupil-to-teacher ratios, other aspects of education quality, such as teacher training, instructional materials, and classroom dynamics, may still be affected by rapid expansion, potentially limiting learning outcomes.

The model assumes rollout through existing administrative systems, including social registries and school-based attendance monitoring. The programme scale is moderate relative to existing social protection systems and is implemented over a four-year period, supporting the feasibility of delivery through these channels. In practice, effectiveness will depend on registry accuracy and grievance redress mechanisms to minimise inclusion and exclusion errors. Conditionality verification may impose administrative burdens on schools, and payment delivery systems could face logistical delays, particularly in remote areas. Depending on their severity, these factors could attenuate overall impacts and returns.

We assumed equal economic returns to education for boys and girls. Although evidence suggests that returns to education may differ by gender, with some studies indicating higher marginal returns for women despite lower absolute earnings, our sensitivity analysis indicates that varying this parameter has a relatively small influence on overall projected wage returns [72,97].

Estimates of future earnings gains assume a causal relationship between schooling and productivity in adulthood [71,72]. However, recent long-term follow-ups from early cash transfer evaluations show mixed results, highlighting that improvements in educational attainment do not always translate directly into improved labour market outcomes, especially in contexts with limited job creation [28,98–100]. Nevertheless, Kenya’s more diversified and growing economy, including expansion in the services and information technology sectors, may offer greater opportunities for skilled youth compared to contexts where many long-term follow-up studies have been conducted [101]. Additionally, ongoing government investments in education quality may enhance the value of educational attainment [102].

While most of our model effectiveness parameters were derived from high-quality experimental studies, some parameters required additional assumptions. For example, the effects of parenting support on sexual violence were informed by observational evidence, as randomised trials are rarely powered to detect such outcomes [64]. Additionally, not all evaluations included in the meta-analysis informing education impact parameters were conducted among both sexes. We therefore assumed similar effects for boys and girls. While this may introduce some uncertainty, the broader evidence base, including both girl-focused and mixed-sex programmes, indicates comparable educational impacts for boys and girls [22,103–105].

Although Kenya-specific evidence was prioritised wherever possible, some effectiveness parameters were informed by studies from Tanzania and South Africa where equivalent Kenyan evidence was unavailable [40,106]. These studies were selected because they were conducted in sub-Saharan African settings that are likely to be more comparable to Kenya than broader regional or global evidence. Nevertheless, differences in programme implementation, social norms, and economic context may affect the transferability of effect estimates, and future analyses would benefit from additional Kenya-specific evidence.

There are also limitations in the population parameters used. Outcomes based on self-reported measures, particularly sensitive experiences such as sexual violence and child marriage, may be subject to reporting bias, leading to conservative estimates of their true burden.

Finally, while the programme is pro-poor by design, more granular data and model extensions would enable further disaggregation of impacts by key characteristics (e.g., age, geography, baseline schooling status) for distributional cost-effectiveness analysis. This represents a key area for future work. In addition, broader socio-economic and environmental changes, including climate-related shocks, may influence both programme impacts and economic returns over time. Better understanding and incorporating these risks in future analyses is also important.

Despite these limitations, this study provides robust insights through its integration of high-quality evidence, context-specific data, and modelling of multiple outcome pathways. The findings highlight the value of cross-sectoral, multi-year interventions that address multiple dimensions of adolescent wellbeing, while emphasising the importance of careful targeting, maintaining education quality, and adapting programme design to local contexts. As adolescent cash ‘plus’ programming is scaled through the State Department of Children’s Services, coordinated planning and budgeting across the social protection, health, education, and child protection sectors could help maximise these cross-sectoral benefits.

### Conclusion

Kenya’s planned investments in adolescents cash ‘plus’ programming have the potential to improve access to education, delay pregnancy and marriage, and prevent violence while delivering strong economic returns and good value for money. Using economic methods, we find that these investments are important both socially and economically, offering high returns in terms of future productivity.

## Supporting information

S1 Appendix Supplementary methods, model descriptions, CHEERS checklist, supporting tables, figures, and evidence syntheses. (DOCX)

## References

[1] Bloom DE, Canning D, Sevilla J. The demographic dividend: a new perspective on the economic consequences of population change. Santa Monica, California: RAND; 2003.

[2] UNFPA. The state of world population 2014. Report No. New York, USA: UNFPA; 2014. Available from: https://www.unfpa.org/sites/default/files/pub-pdf/EN-SWOP14-Report_FINAL-web.pdf

[3] Ministry of Health, Republic of Kenya. National Adolescent Sexual and Reproductive Health Policy 2015 [Internet]. Nairobi, Kenya: The Government of Kenya; 2015 [cited 2024 Apr 16]. Report No. Available from: https://tciurbanhealth.org/wp-content/uploads/2018/03/Ministry-of-Health-ASRH-POLICY-2015.pdf

[4] State Department of Children’s Services, Ministry of Labour and Social Protection, Republic of Kenya. National prevention & response plan on violence against children in Kenya 2019-2023. Nairobi, Kenya: The Government of Kenya; 2020. Available from: https://www.unicef.org/kenya/media/1526/file/National%20Prevention%20&%20Response%20Plan%20on%20VAC.pdf

[5] Karianjahi N, Mbogo J, Wambugu C, Tole J, Agweyu A. Embracing the challenge of adolescent health in Kenya. Lancet Child Adolesc Health. 2020;4(2):101–3. doi: 10.1016/S2352-4642(19)30374-8 31740409 PMC7613527

[6] UNICEF. UNICEF Data. HIV/ AIDS Data. Available from: https://data.unicef.org/topic/hivaids/global-regional-trends/. 2023. Accessed 2024 April 16.

[7] UNICEF. UNICEF Data. Available from: https://data.unicef.org/topic/education/secondary-education/. 2022. Accessed 2024 April 16.

[8] United Nations Development Programme (UNDP), United Nations Entity for Gender Equality and the Empowerment of Women (UN Women). The paths to equal: twin indices on women’s empowerment and gender equality. New York: United Nations; 2023. Available from: https://hdr.undp.org/content/paths-equal

[9] Maughan-Brown B, Banougnin BH, Little MT, Hertzog L, Matsha-Carpentier N, Mugambi C, et al. Tackling the triple threat in Kenya: factors associated with protection against HIV risk, gender-based violence, and pregnancy among adolescent girls and young women. AIDS Behav. 2025;29(6):1738–46. doi: 10.1007/s10461-025-04643-9 39939479 PMC12075351

[10] Sheehan P, Sweeny K, Rasmussen B, Wils A, Friedman HS, Mahon J, et al. Building the foundations for sustainable development: a case for global investment in the capabilities of adolescents. The Lancet. 2017;390(10104):1792–806. doi: 10.1016/s0140-6736(17)30872-3 28433259

[11] Sheehan P, Rasmussen B, Sweeny K, Maharaj N, Symons J, Davidson S, et al. Adolescents in a changing world: the case for urgent investment. Melbourne, Australia: Victoria Institute of Strategic Economic Studies; 2023. Available from: https://pmnch.who.int/resources/publications/m/item/adolescents-in-a-changing-world-the-case-for-urgent-investment

[12] Citi & Plan International. The case for hollistic investment in girls: Improving lives, realizing potential, benefitting everyone. New York, NY: Citi GPS: Global Perspectives & Solutions; 2020. Available from: https://www.citiwarrants.com/home/upload/citi_research/AXJ20.pdf

[13] Crone EA, Dahl RE. Understanding adolescence as a period of social-affective engagement and goal flexibility. Nat Rev Neurosci. 2012;13(9):636–50. doi: 10.1038/nrn3313 22903221

[14] Patton GC, Sawyer SM, Santelli JS, Ross DA, Afifi R, Allen NB, et al. Our future: a Lancet commission on adolescent health and wellbeing. Lancet. 2016;387(10036):2423–78. doi: 10.1016/S0140-6736(16)00579-1 27174304 PMC5832967

[15] Snilstveit B, Stevenson J, Phillips D, Vojtkova M, Gallagher E, Schmidt T, et al. Interventions for improving learning outcomes and access to education in low- and middle- income countries: a systematic review. London: International Initiative for Impact Evaluation (3ie); 2015. Available from: http://3ieimpact.org/evidence-hub/publications/systematic-reviews/interventions-improving-learning-outcomes-and-access

[16] Salam RA, Das JK, Lassi ZS, Bhutta ZA. Adolescent health interventions: conclusions, evidence gaps, and research priorities. J Adolesc Health. 2016;59(4, Supplement):S88-92. doi: 10.1016/j.jadohealth.2016.05.006 PMC5026678 27664599

[17] Ekwunife OI, Ejie IL, Okelu V, Mita C, Durosinmi-Eti O, Powell A. Interventions to increase the uptake and continuation of pre-exposure prophylaxis (PrEP) by adolescent girls and young women at high risk of HIV in low-income and middle-income countries: a scoping review. BMJ Glob Health. 2022;7(12). doi: 10.1136/bmjgh-2022-009474 PMC9743293 36593640

[18] UNICEF. Financing social protection in Eastern and Southern Africa. UNICEF; 2025. Available from: https://www.unicef.org/esa/media/15466/file/UNICEF-Financing-Social-Protection-ESAR-2025.pdf

[19] Malhotra A, Elnakib S. 20 years of the evidence base on what works to prevent child marriage: a systematic review. J Adolesc Health. 2021;68(5):847–62. doi: 10.1016/j.jadohealth.2020.11.017 33446401

[20] Cirillo C, Palermo T, Viola F. Non-contributory social protection and adolescents in lower- and middle-income countries: a review of government programming and impacts. Florence: UNICEF Office of Research; 2021. Available from: https://www.unicef-irc.org/publications/1245-non-contributory-social-protection-and-adolescents-in-lower-and-middle-income-countries-a-review-of-government-programming.html

[21] Kabeer N, Waddington H. Economic impacts of conditional cash transfer programmes: a systematic review and meta-analysis. J Dev Eff. 2015;7(3):290–303. doi: 10.1080/19439342.2015.1068833

[22] Bastagli F, Hagen-Zanker J, Harman L, Barka V, Sturge G, Schmidt T, et al. Cash transfers: what does the evidence say? A rigorous review of impacts and the role of design and implementation features. London: Overseas Development Institute; 2016. Available from: https://www.odi.org/sites/odi.org.uk/files/resource-documents/11316.pdf

[23] Kneale D, Kjaersgaard A, de Melo M, Joaquim Picardo J, Griffin S, French RS, et al. Can cash transfer interventions increase contraceptive use and reduce adolescent birth and pregnancy in low and middle income countries? A systematic review and meta-analysis. PLOS Glob Public Health. 2023;3(11):e0001631. doi: 10.1371/journal.pgph.0001631 37943721 PMC10635429

[24] Handa S, Peterman A, Huang C, Halpern C, Pettifor A, Thirumurthy H. Impact of the Kenya cash transfer for orphans and vulnerable children on early pregnancy and marriage of adolescent girls. Soc Sci Med. 2015;141:36–45. doi: 10.1016/j.socscimed.2015.07.024 26246032 PMC4659857

[25] Feyissa GT, Tolu LB, Soboka M, Ezeh A. Effectiveness of interventions to reduce child marriage and teen pregnancy in sub-Saharan Africa: a systematic review of quantitative evidence. Front Reprod Health. 2023;5:1105390. doi: 10.3389/frph.2023.1105390 37064827 PMC10103588

[26] World Health Organization. Closing the gap in a generation: Health equity through action on the social determinants of health. Geneva: World Health Organization; 2008. Available from: https://iris.who.int/bitstream/handle/10665/69832/WHO_IER_CSDH_08.1_eng.pdf?sequence=1

[27] Blankenship KM, Friedman SR, Dworkin S, Mantell JE. Structural interventions: concepts, challenges and opportunities for research. J Urban Health. 2006;83(1):59–72. doi: 10.1007/s11524-005-9007-4 16736355 PMC1473169

[28] Millán TM, Barham T, Macours K, Maluccio JA, Stampini M. Long-term impacts of conditional cash transfers: review of the evidence. World Bank Res Obs. 2019;34(1):119–59. doi: 10.1093/wbro/lky005

[29] Cluver L, Sherr L. Cash transfers—magic bullet or fundamental ingredient? Lancet Glob Health. 2016;4(12):e883-4. doi: 10.1016/S2214-109X(16)30295-9 27815147

[30] Roelen K, Devereux S, Abdulai AG, Martorano B, Palermo T, Ragno LP. How to make ‘cash plus’ work: linking cash transfers to services and sectors. Florence, Italy: UNICEF Office of Research - Innocenti; 2017. Available from: https://www.calpnetwork.org/publication/how-to-make-cash-plus-work-linking-cash-transfers-to-services-and-sectors/

[31] Machado DB, de Siqueira Filha NT, Cortes F, Castro-de-Araujo LFS, Alves FJO, Ramos D, et al. The relationship between cash-based interventions and violence: a systematic review and evidence map. Aggress Violent Behav. 2024;75:101909. doi: 10.1016/j.avb.2023.101909 40949695 PMC12425482

[32] Austrian K, Soler-Hampejsek E, Kangwana B, Wado YD, Abuya B, Maluccio JA. Impacts of two-year multisectoral cash plus programs on young adolescent girls’ education, health and economic outcomes: Adolescent Girls Initiative-Kenya (AGI-K) randomized trial. BMC Public Health. 2021;21(1):2159. doi: 10.1186/s12889-021-12224-3 34819047 PMC8613919

[33] Austrian K, Soler-Hampejsek E, Kangwana B, Maddox N, Diaw M, Wado YD, et al. Impacts of multisectoral cash plus programs on marriage and fertility after 4 years in pastoralist Kenya: a randomized trial. J Adolesc Health. 2022;70(6):885–94. doi: 10.1016/j.jadohealth.2021.12.015 35168885

[34] Kangwana B, Austrian K, Soler-Hampejsek E, Maddox N, Sapire RJ, Wado YD, et al. Impacts of multisectoral cash plus programs after four years in an urban informal settlement: Adolescent Girls Initiative-Kenya (AGI-K) randomized trial. PLoS One. 2022;17(2):e0262858. doi: 10.1371/journal.pone.0262858 35130299 PMC8820646

[35] Austrian K, Maluccio JA, Soler-Hampejsek E, Muluve E, Aden A, Wado YD, et al. Long-term impacts of a cash plus program on marriage, fertility, and education after six years in pastoralist Kenya: a cluster randomized trial. SSM Popul Health. 2024;26:101663. doi: 10.1016/j.ssmph.2024.101663 38577063 PMC10992718

[36] Cluver LD, Orkin FM, Meinck F, Boyes ME, Yakubovich AR, Sherr L. Can social protection improve sustainable development goals for adolescent health? PLOS ONE. 2016;11(10):e0164808. doi: 10.1371/journal.pone.0164808 PMC5066949 27749932

[37] Pedersen GA, Smallegange E, Coetzee A, Hartog K, Turner J, Jordans MJD. A systematic review of the evidence for family and parenting interventions in low- and middle-income countries: child and youth mental health outcomes. J Child Fam Stud. 2019;28(8):2036–55. doi: 10.1007/s10826-019-01399-4

[38] Lachman J, Wamoyi J, Spreckelsen T, Wight D, Maganga J, Gardner F. Combining parenting and economic strengthening programmes to reduce violence against children: a cluster randomised controlled trial with predominantly male caregivers in rural Tanzania. BMJ Glob Health. 2020;5(7):e002349. doi: 10.1136/bmjgh-2020-002349 32641291 PMC7348478

[39] Gardner F, Shenderovich Y, McCoy A, Martin M, Janowski R, Varadan S. WHO guidelines on parenting interventions to prevent maltreatment and enhance parent–child relationships with children aged 0–17 years: report of the reviews for the WHO-INTEGRATE framework. 2023.

[40] Lachman J, Wamoyi J, Martin M, Han Q, Alfaro FAC, Mgunga S, et al. Reducing family and school-based violence at scale: a large-scale pre–post study of a parenting programme delivered to families with adolescent girls in Tanzania. BMJ Global Health. 2024;9(11):e015472. doi: 10.1136/bmjgh-2024-015472 PMC11590853 39581633

[41] Özler B, Hallman K, Guimond M-F, Kelvin EA, Rogers M, Karnley E. Girl empower - a gender transformative mentoring and cash transfer intervention to promote adolescent wellbeing: impact findings from a cluster-randomized controlled trial in Liberia. SSM Popul Health. 2019;10:100527. doi: 10.1016/j.ssmph.2019.100527 31890847 PMC6928354

[42] Waidler J, Prencipe L, Tirivayi N, Mnyawami Lukongo T, Luchemba P, Eeataama F, et al. Post-intervention gendered impacts and moderating factors of a government cash plus intervention for adolescents in Tanzania. SSM - Popul Health. 2025;29:101760. doi: 10.1016/j.ssmph.2025.101760 40007631 PMC11850116

[43] Palermo T, Prencipe L, Kajula L. Effects of government-implemented cash plus model on violence experiences and perpetration among adolescents in Tanzania, 2018‒2019. Am J Public Health. 2021;111(12):2227–38. doi: 10.2105/AJPH.2021.306509 34878869 PMC8667840

[44] Chzhen Y, Prencipe L, Eetaama F, Luchemba P, Lukongo TM, Palermo T. Impacts of a cash plus intervention on gender attitudes among Tanzanian adolescents. J Adolesc Health. 2021;68(5):899–905. doi: 10.1016/j.jadohealth.2020.07.025 32843241

[45] Devonald M, Guglielmi S, Jones N. Investing in adolescent girls: mapping global and national funding patterns from 2016-2020. London: Gender and Adolescence: Global Evidence; 2023. Available from: https://www.gage.odi.org/wp-content/uploads/2023/01/Investing-in-Adolescent-Girls-2-2.pdf

[46] Devonald M, Guglielmi S, Jones N. Investing in adolescent girls: key changes in the bilateral donor funding landscape - 2021 update. London: Gender and Adolescence: Global Evidence; 2023. Available from: https://www.gage.odi.org/publication/investing-in-adolescent-girls-key-changes-in-the-bilateral-donor-funding-landscape-2021-update/

[47] Dhailiwal ID, Duflo ED, Glennerster R, Tulloch C. Comparative cost-effectiveness analysis to inform policy in developing countries: a general framework with applications for education. 2013. Available from: https://dspace.mit.edu/handle/1721.1/116111

[48] Hove F, Chesshire E, Dyson E, Aliga D. An investment case for child sensitive social protection in East and Southern Africa. Nairobi, Kenya: Save the Children International East and Southern Africa Regional Office; 2024. Available from: https://resourcecentre.savethechildren.net/pdf/an-investment-case-for-cssp-in-east-and-southern-africa_8july2024-single-view.pdf

[49] Austrian K, Muluve E, Nanjekho R, Maluccio J, Soler-Hampejsek E. Adolescent girls initiative–Kenya: testing for scale—recommendations report. Nairobi: Population Council; 2024. Available from: https://knowledgecommons.popcouncil.org/focus_adolescents/119

[50] Husereau D, Drummond M, Petrou S, Carswell C, Moher D, Greenberg D. Consolidated health economic evaluation reporting standards (CHEERS) statement. BMJ. 2013;346:f1049. doi: 10.1136/bmj.f1049 23529982

[51] UNICEF Kenya. Desk review on replacement of households in Kenya’s National Safety Net Programme. Nairobi: UNICEF; 2025. Available from: https://www.unicef.org/kenya/media/4456/file/Technical%20paper%20on%20NSNP%20replacements.pdf.pdf

[52] Cluver LD, Meinck F, Steinert JI, Shenderovich Y, Doubt J, Herrero Romero R, et al. Parenting for Lifelong Health: a pragmatic cluster randomised controlled trial of a non-commercialised parenting programme for adolescents and their families in South Africa. BMJ Glob Health. 2018;3(1):e000539. doi: 10.1136/bmjgh-2017-000539 29564157 PMC5859808

[53] UNICEF. Child Marriage Data. Available from: https://data.unicef.org/topic/child-protection/child-marriage/. 2023. Accessed 2024 April 16.

[54] World Health Organization. Adolescent pregnancy. Available from: https://www.who.int/news-room/fact-sheets/detail/adolescent-pregnancy. 2024. Accessed 2025 July 15.

[55] UNESCO, Methods and Analysis Division. The Unesco Educational Simulation Model (ESM). 29. Paris: UNESCO. 1974. Available from: https://files.eric.ed.gov/fulltext/ED095876.pdf

[56] Kenya National Bureau of Statistics, ICF. Kenya demographic and health survey 2022: Volume 1. Nairobi, Kenya, and Rockville, Maryland: KNBS and ICF; 2023. Available from: https://www.dhsprogram.com/methodology/survey/survey-display-566.cfm

[57] Keiding N. Statistical inference in the Lexis diagram. Philos Trans R Soc Lond Ser Phys Eng Sci. 1997;332(1627):487–509. doi: 10.1098/rsta.1990.0128

[58] Drummond MF, Sculpher MJ, Claxton K, Stoddart GL, Torrance GW. Methods for the economic evaluation of health care programmes. Fourth Edition ed. Oxford University Press; 2015.

[59] Rudgard W, Asuma B, Desmond C, Bandyopadhyay R, Obiesie S, Grueso H. Human development impacts and economic returns from investing in adolescents in Kenya. 2026. Available from: https://osf.io/62b53 10.1371/journal.pgph.0006822 PMC13399278 42497171

[60] Together for Girls. Kenya violence against children and youth survey (VACS) report 2020. Available from: https://www.togetherforgirls.org/en/resources/kenya-vacs-report-2019. Accessed 2024 February 28.

[61] Handa S, Palermo T, Rosenberg M, Pettifor A, Halpern CT, Thirumurthy H. How does a national poverty programme influence sexual debut among Kenyan adolescents? Glob Public Health. 2017;12(5):5. doi: 10.1080/17441692.2015.1134617 PMC4976080 26853950

[62] The Kenya CT-OVC Evaluation Team. The impact of Kenya’s cash transfer for orphans and vulnerable children on human capital. J Dev Eff. 2012;4(1):38–49. doi: 10.1080/19439342.2011.653578

[63] Wamoyi J, Lachman J, Martin M, Shenderovich Y, Manjengenja N, Ndyetabura E, et al. The Furaha Adolescent Implementation Research (FAIR) study final report. The Evaluation Fund; 2022. Available from: https://theevaluationfund.net/wp-content/uploads/2020/10/FAIR-Final-Report.pdf

[64] Cluver LD, Rudgard WE, Toska E, Zhou S, Campeau L, Shenderovich Y, et al. Violence prevention accelerators for children and adolescents in South Africa: a path analysis using two pooled cohorts. PLOS Medicine. 2020;17(11):e1003383. doi: 10.1371/journal.pmed.1003383 PMC7652294 33166288

[65] Lachman JM, Alampay LP, Jocson RM, Alinea C, Madrid B, Ward C, et al. Effectiveness of a parenting programme to reduce violence in a cash transfer system in the Philippines: RCT with follow-up. Lancet Reg Health West Pac. 2021;17:100279. doi: 10.1016/j.lanwpc.2021.100279 34734199 PMC8501762

[66] Ward P, Hurrell A, Visram A, Riemenschneider N, O’Brien C, MacAuslan I. Cash transfer programme for orphans and vulnerable children, Kenya operational and impact evaluation, 2007–2009. Oxford: Oxford Policy Management; 2010. Available from: https://www.opml.co.uk/files/Publications/6020-cash-transfer-OVC-kenya/opm-ct-ovc-evaluation-report-july2010-final-kenya-2010-019.pdf

[67] J-PAL. Conducting cost-effectiveness analysis (CEA). Available from: https://www.povertyactionlab.org/resource/conducting-cost-effectiveness-analysis-cea. Accessed 2025 April 13.

[68] World Bank Group. The World Bank. Development Projects: Kenya Cash Transfer for Orphans and Vulnerable Children - P111545. Available from: https://projects.worldbank.org/en/projects-operations/document-detail/P111545

[69] Kenya Institute for Public Policy Research and Analysis. Education and Training Budget Brief 2018-2019. Nairobi, Kenya: Kenya Institute for Public Policy Research and Analysis; 2018. Available from: http://repository.kippra.or.ke/handle/123456789/2268

[70] Teachers Service Commission. TSC circular on new teachers salaries and allowances August 2024. Available from: https://arena.co.ke/tsc-circular-on-new-teachers-salaries-and-allowances-august-2024/. 2024. Accessed 2025 August 17.

[71] Perumal N, Blakstad MM, Fink G, Lambiris M, Bliznashka L, Danaei G, et al. Impact of scaling up prenatal nutrition interventions on human capital outcomes in low- and middle-income countries: a modeling analysis. Am J Clin Nutr. 2021;114(5):1708–18. doi: 10.1093/ajcn/nqab234 34320177 PMC8574629

[72] Patrinos HA, Psacharopoulos G. Returns to education in developing countries. The economics of education. Second ed. Cambridge, Mass: Academic Press; 2020. p. 53–64. Available from: doi: 10.1016/B978-0-12-815391-8.00004-5

[73] Neumann PJ, Sanders GD, Russell LB, Siegel JE, Ganiats TG. Cost-effectiveness in health and medicine. Oxford, UK: Oxford University Press; 2016.

[74] World Bank. World Bank Open Data. Available from: https://data.worldbank.org/indicator/FP.CPI.TOTL.ZG. Accessed 2025 April 12.

[75] Central Bank of Kenya. Foreign exchange rates. Available from: https://www.centralbank.go.ke/forex/. Accessed 2025 April 12.

[76] Baird S, McIntosh C, Özler B. When the money runs out: do cash transfers have sustained effects on human capital accumulation? J Dev Econ. 2019;140:169–85. doi: 10.1016/j.jdeveco.2019.04.004

[77] Grueso H, Desmond C, Rudgard WE, Obiesie S, Cluver L. Get three for the price of one: evaluating the cost-effectiveness of multi-outcome interventions. In: SSRN Scholarly Paper, 2024. Available from: https://papers.ssrn.com/abstract=4527863

[78] Dhaliwal I, Duflo E, Glennerster R, Tulloch C. Comparative cost-effectiveness analysis to inform policy in developing countries: a general framework with applications for education. In: Glewwe P, editor. Education policy in developing countries. University of Chicago Press. 2013. p. 0. doi: 10.7208/chicago/9780226078854.003.0008

[79] World Bank. World Bank Open Data. Available from: https://data.worldbank.org/indicator/NY.GDP.MKTP.CD. Accessed 2025 April 13.

[80] Kenya National Bureau of Statistics. 2023/24 Kenya Housing Survey - Basic Report. Nairobi: Kenya National Bureau of Statistics; 2025. Available from: https://www.knbs.or.ke/wp-content/uploads/2025/01/2023-24-Kenya-Housing-Survey-Basic-Report1.pdf

[81] Kenya National Bureau of Statistics. Comprehensive poverty report. Nairobi: Kenya National Bureau of Statistics; 2020. Available from: https://www.genderinkenya.org/wp-content/uploads/2020/08/CPR-Report-10_08_2020.pdf

[82] Kenya State Department for Social Protection. Management Information System (MIS) data on cash transfer beneficiaries by county and adolescent presence in household; 2025.

[83] World Bank. World Bank Open Data. Available from: https://data.worldbank.org/indicator/SP.POP.GROW. Accessed 2026 March 3.

[84] Duflo E, Dupas P, Kremer M. Education, HIV, and early fertility: experimental evidence from Kenya. Am Econ Rev. 2015;105(9). doi: 10.1257/aer.20121607 PMC4624413 26523067

[85] Kremer M, Miguel E, Thornton R. Incentives to learn. Rev Econ Stat. 2009;91(3):437–56. doi: 10.1162/rest.91.3.437

[86] Heckman JJ, Masterov DV. The productivity argument for investing in young children. Appl Econ Perspect Policy. 2007;29(3):446–93. doi: 10.1111/j.1467-9353.2007.00359.x

[87] Schulenberg J, Maslowsky J. Contribution of adolescence to the life course: what matters most in the long run? In: The study of human development. Routledge; 2018. 10.1080/15427609.2015.1068039 PMC4602400 26478720

[88] Desmond C, Erzse A, Watt K, Ward K, Newell M-L, Hofman K, et al. Realising the potential human development returns to investing in early and maternal nutrition: the importance of identifying and addressing constraints over the life course. PLOS Glob Public Health. 2021;1(10):e0000021. doi: 10.1371/journal.pgph.0000021 36962074 PMC10022083

[89] United Nations Population Division, Department of Economic and Social Affairs. 2019 Revision of World Population Prospects; 2019.

[90] Ozier O. The impact of secondary schooling in Kenya: a regression discontinuity analysis. J Hum Resour. 2018;53(1):157–88. Available from: https://www.jstor.org/stable/26450492

[91] Karoly LA, Kilburn MR, Cannon JS. Early childhood interventions: proven results, future promise. RAND Corporation; 2005. Available from: https://www.rand.org/pubs/monographs/MG341.html

[92] Remme M. Cross-sectoral co-financing: taking a multi-payer perspective in the financing and economic evaluation of structural HIV interventions. London School of Hygiene & Tropical Medicine; 2018. Available from: https://researchonline.lshtm.ac.uk/id/eprint/4647055/

[93] Welte R, Feenstra T, Jager H, Leidl R. A decision chart for assessing and improving the transferability of economic evaluation results between countries. Pharmacoeconomics. 2004;22(13):857–76. doi: 10.2165/00019053-200422130-00004 15329031

[94] Turner HC, Hori Y, Revill P, Rattanavipapong W, Arai K, Nonvignon J. Analyses of the return on investment of public health interventions: a scoping review and recommendations for future studies. BMJ Glob Health. 2023;8(8):e012798. doi: 10.1136/bmjgh-2023-012798 37648275 PMC10471881

[95] Kilburn K, Thirumurthy H, Halpern CT, Pettifor A, Handa S. Effects of a large-scale unconditional cash transfer program on mental health outcomes of young people in Kenya. J Adolesc Health. 2016;58(2):223–9. doi: 10.1016/j.jadohealth.2015.09.023 26576822 PMC4724529

[96] Ha NT, Huong NT, Anh VN, Anh NQ. Modelling in economic evaluation of mental health prevention: current status and quality of studies. BMC Health Serv Res. 2022;22(1):906. doi: 10.1186/s12913-022-08206-9 35831821 PMC9281039

[97] Barouni M, Broecke S. The returns to education in Africa: some new estimates. J Dev Stud. 2014;50(12):1593–613. doi: 10.1080/00220388.2014.936394

[98] Baird S, McKenzie D, Özler B. The effects of cash transfers on adult labor market outcomes. IZA J Dev Migr. 2018;8(1):22. doi: 10.1186/s40176-018-0131-9

[99] Parker SW, Vogl T. Do conditional cash transfers improve economic outcomes in the next generation? Evidence from Mexico. Econ J. 2023;133(655). doi: 10.1093/ej/uead049

[100] Handa S, Tembo G, Natali L, Chakrabarti A. In search of the holy grail: post-intervention effects of an unconditional cash transfer program in Zambia. J Dev Econ. 2025;174:103454. doi: 10.1016/j.jdeveco.2025.103454

[101] Filmer D, Fox L. Youth employment in Sub-Saharan Africa. Washington, DC: World Bank; 2014. Available from: https://documents1.worldbank.org/curated/en/424011468192529027/pdf/Full-report.pdf

[102] African Union Commission, UNICEF. Education spending in Africa: the impacts of COVID-19 and possible recovery pathways. Nairobi: UNICEF Eastern and Southern Africa; 2024. Available from: https://www.unicef.org/esa/media/13876/file/Education%20Spending%20in%20Africa%20%20The%20impacts%20of%20COVID-19%20and%20possible%20recovery%20pathways.pdf.pdf

[103] Baird S, Ferreira FHG, Özler B, Woolcock M. Conditional, unconditional and everything in between: a systematic review of the effects of cash transfer programmes on schooling outcomes. J Dev Eff. 2014;6(1):1–43. doi: 10.1080/19439342.2014.890362

[104] García S, Saavedra JE. Educational impacts and cost-effectiveness of conditional cash transfer programs in developing countries: a meta-analysis. Rev Educ Res. 2017;87(5). doi: 10.3102/0034654317723008

[105] Evans DK, Gale C, Kosec K. The educational impacts of cash transfers in Tanzania. Econ Educ Rev. 2023;92:102332. doi: 10.1016/j.econedurev.2022.102332

[106] Cluver LD, Meinck F, Steinert JI, Shenderovich Y, Doubt J, Herrero Romero R, et al. Parenting for Lifelong Health: a pragmatic cluster randomised controlled trial of a non-commercialised parenting programme for adolescents and their families in South Africa. BMJ Glob Health. 2018;3(1):e000539. doi: 10.1136/bmjgh-2017-000539 29564157 PMC5859808

