# Supplementary material for: Estimating the human development impacts and economic returns from an adolescent cash ‘plus’ programme in Kenya: An economic modelling study
Source: PLOS Glob Public Health. 2026 Jul 24;6(7):e0006822. doi: 10.1371/journal.pgph.0006822 (PMC13399278; doi:10.1371/journal.pgph.0006822)
Supplement: S1 Appendix — (DOCX) [file pgph.0006822.s001.docx]

**S1 Appendix. Supplementary methods, model descriptions, CHEERS checklist, supporting tables, figures, and evidence syntheses.**

# Table A CHEERS Checklist.

| Section/topic | # | Recommendation | Reported on page #/ line # |
| --- | --- | --- | --- |
| TITLE AND ABSTRACT | | | |
| Title | 1 | Identify the study as an economic evaluation or use more specific terms such as “cost-effectiveness analysis”, and describe the interventions compared. | 1 |
| Abstract | 2 | Provide a structured summary of objectives, perspective, setting, methods (including study design and inputs), results (including base case and uncertainty analyses), and conclusions. | 1 |
| INTRODUCTION | |  | |
| Background and objectives | 3 | Provide an explicit statement of the broader context for the study.  Present the study question and its relevance for health policy or practice decisions. | 2/3 |
|  | | | |
| Target population and subgroups | 4 | Describe characteristics of the base case population and subgroups analysed, including why they were chosen. | 3/4 |
| Setting and location | 5 | State relevant aspects of the system(s) in which the decision(s) need(s) to be made. | 3/4 |
| Study perspective | 6 | Describe the perspective of the study and relate this to the costs being evaluated. | 8 |
| Comparators | 7 | Describe the interventions or strategies being compared and state why they were chosen. | 4 |
| Time horizon | 8 | State the time horizon(s) over which costs and consequences are being evaluated and say why appropriate. | 8 |
| Discount rate | 9 | Report the choice of discount rate(s) used for costs and outcomes and say why appropriate. | 8 |
| Choice of health outcomes | 10 | Describe what outcomes were used as the measure(s) of benefit in the evaluation and their relevance for the type of analysis performed. | 5 |
| Measurement of effectiveness | 11a | Single study-based estimates: Describe fully the design features of the single effectiveness study and why the single study was a sufficient source of clinical effectiveness data | 6/7 |
|  | 11b | Synthesis-based estimates: Describe fully the methods used for identification of included studies and synthesis of clinical effectiveness data. | 6/7 and supplementary material |
| Measurement and valuation of preference-based outcomes | 12 | If applicable, describe the population and methods used to elicit preferences for outcomes | NA |
| Estimating resources and costs | 13a | Single study-based economic evaluation: Describe approaches used to estimate resource use associated with the alternative interventions. Describe primary or secondary research methods for valuing each resource item in terms of its unit cost. Describe any adjustments made to approximate to opportunity costs. |  |
|  | 13b | Model-based economic evaluation: Describe approaches and data sources used to estimate resource use associated with model health states. Describe primary or secondary research methods for valuing each resource item in terms of its unit cost. Describe any adjustments made to approximate to opportunity costs. | 7/8 |
| Currency, price date, and conversion | 14 | Report the dates of the estim­­­­ated resource quantities and unit costs. Describe methods for adjusting estimated unit costs to the year of reported costs if necessary. Describe methods for converting costs into a common currency base and the exchange rate. | 8 |
| Choice of model | 15 | Describe and give reasons for the specific type of decision-analytical model used. Providing a figure to show model structure is strongly recommended. | 5 |
| Assumptions | 16 | Describe all structural or other assumptions underpinning the decision-analytical model. | 5 |
| Analytical models | 17 | Describe all analytical methods supporting the evaluation. This could include methods for dealing with skewed, missing, or censored data; extrapolation methods; methods for pooling data; approaches to validate or make adjustments (such as half cycle corrections) to a model; and methods for handling population heterogeneity and uncertainty. | 5 |
| RESULTS | | | |
| Study parameters | 18 | Report the values, ranges, references, and, if used, probability distributions for all parameters/ Report reasons or sources for distributions used to represent uncertainty where appropriate. Providing a table to show the input values is strongly recommended. | 11 |
| Incremental costs and outcomes | 19 | For each intervention, report mean values for the main categories of estimated costs and outcomes of interest, as well as mean differences between the comparator groups. If applicable, report incremental cost-effectiveness ratios. | 11-13 |
| Characterising uncertainty | 20a | Single study-based economic evaluation: Describe the effects of sampling uncertainty for the estimated incremental cost and incremental effectiveness parameters, together with the impact of methodological assumptions (such as discount rate, study perspective). | 12-13 |
|  | 20b | Model-based economic evaluation: Describe the effects on the results of uncertainty for all input parameters, and uncertainty related to the structure of the model and assumptions. | 12-13 |
| Characterising heterogeneity | 21 | If applicable, report differences in costs, outcomes, or cost-effectiveness that can be explained by variations between subgroups of patients with different baseline characteristics or other observed variability in effects that are not reducible by more information. | 14 |
| DISCUSSION | | | |
| Study findings, limitations, generalisability, and current knowledge | 22 | Summarise key study findings and describe how they support the conclusions reached. Discuss limitations and the generalisability of the findings and how the findings fit with current knowledge. | 17-20 |
| OTHER | | | |
| Source of funding | 23 | Describe how the study was funded and the role of the funder in the identification, design, conduct, and reporting of the analysis. Describe other non-monetary sources of support. | 21 |
| Conflicts of interest | 24 | Describe any potential for conflict of interest of study contributors in accordance with journal policy. Int eh absence of a journal policy, we recommend authors comply with International Committee of Medical Journal Editors recommendations. | 21 |

# Text A. Cohort progression model of educational attainment

This simulation estimates years of schooling across twelve grade cohorts within the Kenyan education system. The model is adapted from the UNESCO Educational Simulation Model (ESM) and projects student enrolment and progression over time (1). Cohorts are defined by their starting grade and followed until they exit the education system. The model is parameterised using baseline data from the 2022 Kenya Demographic and Health Survey (KDHS), reflecting the 8-4-4 education system in place at the time (2).

We compare a baseline scenario with an intervention scenario in which the cash plus programme increases school enrolment and progression (3–6). The intervention effect on progression is modelled as time-varying, with effectiveness persisting for two years following exposure before attenuating. (5,6).

## Structure of the education system.

The 8-4-4 education system that was modelled in this study consists of:

- 8 years of primary education grades $(c=1, \ldots, 8)$
- 4 years of secondary education grades $\left( c=9, \ldots, 12 \right)$

University education is not explicitly modelled.

This model focuses on the primary and secondary cycles of the 8-4-4 system, structured as a directed graph:

- Nodes represent grades $c=1, \ldots, 12$
- Edges define transitions between grades and exits from the system

The model captures:

- *Static state:* Enrolment in grade $c$ at time $t$
- *Dynamic transitions*: Annual changes based on rates of repetition, dropout, and promotion between grades

Students are assumed to progress at most one grade per year.

Time is discrete, with $t=1, 2,\ldots$, where cohorts enter in periods $t=1,\ldots,4$and are followed until they exit the education system.

## Notation.

- $E_{c,t}$: Number of students enrolled in grade $c$ at time $t$
- $P_{t}$: Population eligible for entry at time $t$
- $e_{c,t}$: Entry rate into grade $c$ at time $t$
- $r_{c,t}$: Repetition rate at the end of grade $c$ at time $t$
- $\phi_{c,t}$: Dropout or exit rate at the end of grade $c$ at time $t$
- $d_{c,t}$: Progression rate from grade $c$ to $c+1$ at time $t$, where

$$d_{c,t}=1- r_{c,t}-\phi_{c,t}$$

- $s_{t}$: Surviving rate affecting enrolment time $t$
- $\delta_{t}$: Time-varying intervention effect on progression
- $\lambda$: Intervention effect on initial enrolment

## Initial conditions.

The starting distribution across grades is derived from grade-specific enrolment rates by age, ${ER}_{c}$, applied to the population aged 10-18 at $t=1$.

$$E_{c,1}={ER}_{c}\cdot P_{1}, c=1, \ldots, 12$$

## Model dynamics.

Enrolment evolves according to transitions between grades, repetition, dropout, and new entry:

$$E_{c,t+1}=[E_{c-1,t}\cdot d_{c-1,t}+E_{c,t}\cdot r_{c,t}+P_{t} \cdot e_{c,t}]\cdot s_{t}$$

This represents:

- progression from the previous grade
- repetition within the same grade
- new entrants
- adjustment for survival

## Intervention scenario.

Two mechanisms are modelled.

- 1. Increased initial enrolment due to conditionality

The cash transfer is modelled to increase the grade-specific enrolment rate at $t=1$:

$$E_{c,1}^{int}={ER}_{c}\cdot P_{1}\cdot\lambda, \mathrm{for} c=1, \ldots, 12$$

1. Increased grade progression

The intervention is modelled to increases progression rates:

$$d_{c,t}^{int}=d_{c,t}\cdot\delta_{t}$$

To ensure transition probabilities sum to 1 (with repetition fixed), dropout adjusts:

$$\phi_{c,t}^{int}=1-r_{c,t}-d_{c,t}^{int}$$

The intervention effect $\delta_{t}$ is time-varying and assumed to persist for two years following exposure before attenuating.

## Iteration.

The system is updated sequentially across grades and time periods. Cohorts are followed until they exit the education system (through completion or dropout).

## Estimating intervention impact.

The years of schooling are calculated as cumulative enrolment across all grades and time periods:

- Baseline:

$$T_{baseline}=\sum_{c=1}^{12} \sum_{t} E_{c,t}$$

- Intervention:

$$T_{intervention}=\sum_{c=1}^{12} \sum_{t} E_{c,t}^{int}$$

- Impact:

$$\Delta T=T_{intervention}-T_{baseline}$$

$\Delta T$ represents the additional years of schooling generated by the intervention.

# References

1. Unesco. Methods and Analysis Division. The Unesco Educational Simulation Model (ESM) [Internet]. Paris: Unesco; 1974. (Reports and papers in the social sciences; no. 29). Available from: https://files.eric.ed.gov/fulltext/ED095876.pdf

2. Kenya National Bureau of Statistics, ICF. Kenya Demographic and Health Survey 2022: Volume 1 [Internet]. Nairobi, Kenya, and Rockville, Maryland: KNBS and ICF; 2023 [cited 2024 Feb 28]. Available from: https://www.dhsprogram.com/methodology/survey/survey-display-566.cfm

3. Ward P, Hurrell A, Visram A, Riemenschneider N, O’Brien C, MacAuslan I, et al. Cash transfer programme for orphans and vulnerable children, Kenya operational and impact evaluation, 2007–2009 [Internet]. Oxford: Oxford Policy Management; 2010. Available from: https://www.opml.co.uk/files/Publications/6020-cash-transfer-OVC-kenya/opm-ct-ovc-evaluation-report-july2010-final-kenya-2010-019.pdf?noredirect=1

4. Austrian K, Soler-Hampejsek E, Kangwana B, Wado YD, Abuya B, Maluccio JA. Impacts of two-year multisectoral cash plus programs on young adolescent girls’ education, health and economic outcomes: Adolescent Girls Initiative-Kenya (AGI-K) randomized trial. BMC Public Health. 2021 Nov 24;21:2159.

5. Austrian K, Soler-Hampejsek E, Kangwana B, Maddox N, Diaw M, Wado YD, et al. Impacts of Multisectoral Cash Plus Programs on Marriage and Fertility After 4 Years in Pastoralist Kenya: A Randomized Trial. J Adolesc Health. 2022 Jun 1;70(6):885–94.

6. Austrian K, Maluccio JA, Soler-Hampejsek E, Muluve E, Aden A, Wado YD, et al. Long-term impacts of a cash plus program on marriage, fertility, and education after six years in pastoralist Kenya: A cluster randomized trial. SSM - Popul Health. 2024;26:101663.

# Text B. Cohort life-table model of child marriage

This simulation estimates incident child marriage before age 18 across four time-defined cohorts (girls aged 10–17 at cohort entry in 2026–2029), who are followed until they age out of the risk period. The model is parameterised using age-specific estimates from the 2022 Kenya Demographic and Health Survey (KDHS) and survival probabilities drawn from IHME Global Health Data Exchange (1). We compare a baseline scenario with an intervention scenario in which the cash plus programme reduces the risk of child marriage (2,3). The intervention effect is modelled as time-varying, with effectiveness persisting for two years following exposure before attenuating.

Incidence probabilities can be estimated for ages 12–17; girls younger than 12 are therefore assumed not to be at risk during the simulation period. Rates reported per 100 woman-years were converted to annual probabilities using $\boldsymbol{q=1-}\boldsymbol{e}^{\boldsymbol{-r}}$.

## Notation

- $l_{x,t}^{a}$: Number of girls from cohort $a$ alive and at risk of marriage at age $x$ in time period $t$
- $q_{x}$: Age-specific marriage probability of first marriage between ages $x$ and $x+1$
- $d_{x,t}^{a}$: Number of incident marriages at age $x$ in time period $t$
- $s_{x,t}$: Probability of surviving from age $x$ to $x+1$ in time period $t$
- $\delta_{t}$: Time-varying intervention effect (relative risk), incorporating persistence of programme effects

Where:

- $x=12, \ldots, 17$
- $t=1, 2, 3, 4$
- Cohorts are indexed by entry year $a$, so at $t=1$, $a=1$. Cohorts enter in periods $t=1,\ldots,4$and are followed in subsequent periods until they age out of the risk range.

## Model structure

We use a discrete-time cohort life-table approach in which first marriage is treated as an absorbing event. Individuals who marry are removed from the population at risk in subsequent age intervals. First marriage is modelled as a single absorbing event; subsequent marriages are not considered.

*1. Incident marriages.*

In each time period, incident marriages are given by:

- - Baseline scenario:

$$\begin{matrix} d_{x,t}^{a}= l_{x,t}^{a}\cdot q_{x} \end{matrix}$$

- - Intervention scenario:

$$q_{x}^{int}= q_{x}\cdot\delta_{t}$$

$$d_{x,t}^{a, int}= l_{x,t}^{a}\cdot q_{x}^{int}$$

*2. Cohort transition.*

The population at risk evolves according to:

$$l_{x+1,t+1}^{a}= l_{x,t}^{a}\cdot(1-q_{x})\cdot s_{x,t}$$

In the intervention scenario, $q_{x}$is replaced by $q_{x}^{int}$ in both the incidence and transition equations.

*3. Iteration.*

The above steps are applied sequentially across all ages, time periods, and cohorts until all individuals age out of the risk period.

## Estimating intervention impact

Total incident marriages are obtained by summing across cohorts, time periods, and ages:

- - Baseline:

$$D_{baseline}=\sum_{a} \sum_{t} \sum_{x=12}^{17} d_{x,t}^{a}$$

- - Intervention:

$$D_{intervention}=\sum_{a} \sum_{t} \sum_{x=12}^{17} d_{x,t}^{a, int}$$

- - Intervention:

$$\Delta D=D_{baseline}-D_{intervention}$$

$\Delta D$ represents the number of child marriages averted over the simulation period. Negative values at specific ages or time periods reflect temporal shifts in marriages across ages rather than increases in overall incidence.

# References

1. Kenya National Bureau of Statistics, ICF. Kenya Demographic and Health Survey 2022: Volume 1 [Internet]. Nairobi, Kenya, and Rockville, Maryland: KNBS and ICF; 2023 [cited 2024 Feb 28]. Available from: https://www.dhsprogram.com/methodology/survey/survey-display-566.cfm

2. Austrian K, Soler-Hampejsek E, Kangwana B, Maddox N, Diaw M, Wado YD, et al. Impacts of Multisectoral Cash Plus Programs on Marriage and Fertility After 4 Years in Pastoralist Kenya: A Randomized Trial. J Adolesc Health. 2022 Jun 1;70(6):885–94.

3. Austrian K, Maluccio JA, Soler-Hampejsek E, Muluve E, Aden A, Wado YD, et al. Long-term impacts of a cash plus program on marriage, fertility, and education after six years in pastoralist Kenya: A cluster randomized trial. SSM - Popul Health. 2024;26:101663.

# Text C. Cohort life-table model of adolescent pregnancy and intervention impact

This simulation estimates incident adolescent pregnancy before age 20 across four time-defined cohorts (girls aged 10–19 at cohort entry in 2026–2029), who are followed until they age out of the risk period. The model is parameterised using age-specific estimates from the 2022 Kenya Demographic and Health Survey (KDHS) and survival probabilities drawn from IHME Global Health Data Exchange (1). We compare a baseline scenario with an intervention scenario in which the cash plus programme reduces the risk of adolescent pregnancy (2,3). The intervention effect is modelled as time-varying, with effectiveness persisting for two years following exposure before attenuating.

Incidence probabilities can be estimated for ages 12–19; girls younger than 12 are therefore assumed not to be at risk during the simulation period. Rates reported per 100 woman-years were converted to annual probabilities using $q=1-e^{-r}$.

**Notation**

- $l_{x,t}^{a}$: Number of girls from cohort $a$ alive and at risk at age $x$ in time period $t$
- $q_{x}$: Age-specific probability of first pregnancy between ages $x$ and $x+1$
- $d_{x,t}^{a}$: Number of incident pregnancies at age $x$ in time period $t$
- $s_{x,t}$: Probability of surviving from age $x$ to $x+1$ in time period $t$
- $\delta_{t}$: Intervention effect (relative risk of pregnancy)

For second pregnancies:

- $l_{x,t}^{\left( 2 \right)}$: Number of girls alive at age $x$in time period $t$ with one prior pregnancy (at risk of second pregnancy)
- $q^{\left( 2 \right)}$: Probability of second pregnancy (ages 15–19, pooled)
- $d_{x,t}^{\left( 2 \right)}$: Number of second pregnancies at age $x$in time period $t$

Where:

- $x=12, \ldots, 19$
- $t=1, 2, 3, 4$
- Cohorts are indexed by entry year $a$, so at $t=1$, $a=1$. Cohorts enter in periods $t=1,\ldots,4$and are followed in subsequent periods until they age out of the risk range.

**Model structure**

We use a discrete-time cohort life-table approach. First pregnancy is treated as an absorbing event with respect to the first-pregnancy risk set. A second-pregnancy process is modelled separately among girls with one prior pregnancy. Third and higher-order pregnancies were not modelled, as rates among adolescents aged 10–19 were low and their contribution to total pregnancies over the simulation horizon was expected to be negligible.

*1. First pregnancies.*

In each time period, incident first pregnancies are given by:

- Baseline scenario:

$$\begin{matrix} d_{x,t}^{a}= l_{x,t}^{a}\cdot q_{x} \end{matrix}$$

- Intervention scenario:

$$q_{x}^{int}= q_{x}\cdot\delta_{t}$$

$$d_{x,t}^{a,int}= l_{x,t}^{a}\cdot q_{x}^{int}$$

*2. Cohort transition (first pregnancy risk set).*

The population at risk evolves according to:

$$l_{x+1,t+1}^{a}= l_{x,t}^{a}\cdot(1-q_{x})\cdot s_{x,t}$$

In the intervention scenario, $q_{x}$is replaced by $q_{x}^{int}$ in both the incidence and transition equations.

*3. Second pregnancies.*

Second pregnancies are modelled using a dynamic post-first-pregnancy risk set. Individuals enter this risk set one year after their first pregnancy and are followed from ages 15–19 using a pooled probability $q^{\left( 2 \right)}$.

- Risk set transition:

$$l_{x+1,t+1}^{\left( 2 \right)}=(l_{x,t}^{\left( 2 \right)}-d_{x,t}^{\left( 2 \right)}+d_{x,t})\cdot s_{x,t}$$

- Second pregnancy events:

$$d_{x,t}^{\left( 2 \right)}=l_{x,t}^{\left( 2 \right)}\cdot q^{\left( 2 \right)},x=15,\ldots,19$$

- Intervention scenario:

$$q^{\left( 2 \right),\text{int}}=q^{\left( 2 \right)}\cdot\delta$$

$$d_{x,t}^{\left( 2 \right),\text{int}}=l_{x,t}^{\left( 2 \right)}\cdot q^{\left( 2 \right),\text{int}}$$

In the absence of separate estimates, the intervention effect was assumed to apply equally to first and subsequent pregnancies.

*4. Iteration.*

All steps are applied sequentially across ages, time periods, and cohorts until all individuals age out of the risk period.

**Estimating intervention impact**

Total incident pregnancies are obtained by summing across ages, time periods, and cohorts.

Baseline:

$$D_{baseline}=\sum_{a} \sum_{t} \sum_{x=12}^{19} {(d}_{x,t}^{a}+d_{x,t}^{(2)})$$

- Intervention:

$$D_{intervention}=\sum_{a} \sum_{t} \sum_{x=12}^{19} {(d}_{x,t}^{a,int}+d_{x,t}^{\left( 2 \right),int})$$

- Intervention impact:

$$\Delta D=D_{baseline}-D_{intervention}$$

$\Delta D$ represents the number of adolescent pregnancies averted over the simulation period. Negative values at specific ages or time periods reflect temporal shifts in pregnancies rather than increases in overall incidence.

**References.**

1. Kenya National Bureau of Statistics, ICF. Kenya Demographic and Health Survey 2022: Volume 1 [Internet]. Nairobi, Kenya, and Rockville, Maryland: KNBS and ICF; 2023 [cited 2024 Feb 28]. Available from: https://www.dhsprogram.com/methodology/survey/survey-display-566.cfm

2. Austrian K, Soler-Hampejsek E, Kangwana B, Maddox N, Diaw M, Wado YD, et al. Impacts of Multisectoral Cash Plus Programs on Marriage and Fertility After 4 Years in Pastoralist Kenya: A Randomized Trial. J Adolesc Health. 2022 Jun 1;70(6):885–94.

3. Austrian K, Maluccio JA, Soler-Hampejsek E, Muluve E, Aden A, Wado YD, et al. Long-term impacts of a cash plus program on marriage, fertility, and education after six years in pastoralist Kenya: A cluster randomized trial. SSM - Popul Health. 2024;26:101663.

# Text D. Prevalence-based model of violence victimisation

This simulation estimates experiences of emotional, physical, and sexual violence across four time-defined cohorts (2026–2029), who are followed until they age out of the risk period. The model is parameterised using age-specific prevalence estimates among adolescents in the poorest wealth quintiles from the 2019 Kenya Violence Against Children Survey (VACS) (1).

We compare a baseline scenario with an intervention scenario in which a parenting support programme reduces the probability of violence (2–4). The intervention effect is modelled as time-varying, with effectiveness persisting for three years following exposure and attenuating by 33% per year (5).

**Notation**

- $N_{x,t}^{a}$: Number of individuals from cohort $a$ alive at age $x$ in time period $t$
- $v_{x}$: Age-specific probability of experiencing violence in the last year
- $V_{x,t}^{a}$: Number of individuals experiencing violence at age $x$ in time period $t$
- $s_{x,t}$: Probability of surviving from age $x$ to $x+1$ in time period $t$
- $\delta_{t}$: Time-varying intervention effect

Where:

- $x=10, \ldots, 21$
- $t=1, 2, 3, 4$
- Cohorts are defined by entry year, with $a=1$ at $t=1$

**Model structure**

We use a discrete-time prevalence-based model in which the expected number of violence victimisation events in each age-year is calculated by applying age-specific last-year prevalence to the population at risk. Prevalence estimates are interpreted as annual probabilities of experiencing at least one event in the past year.

Violence is treated as a repeatable annual event, rather than an absorbing state. Individuals may experience violence in multiple years.

*1. Violence events*

- - Baseline scenario:

$$\begin{matrix} V_{x,t}^{a}= N_{x,t}^{a}\cdot v_{x} \end{matrix}$$

- - Intervention scenario:

$$v_{x}^{int}= v_{x}\cdot\delta_{t}$$

$$V_{x,t}^{a, int}= N_{x,t}^{a}\cdot v_{x}^{int}$$

*2. Cohort transition*

The population at risk evolves as individuals age:

$$N_{x+1,t+1}^{a}= N_{x,t}^{a}\cdot s_{x,t}$$

*3. Iteration*

All steps are applied sequentially across ages, time periods, and cohorts until all individuals age out of the risk period.

**Estimating intervention impact**

Total violence victimisation events are obtained by summing across cohorts, ages, and time periods:

- Baseline:

$$V_{baseline}=\sum_{a} \sum_{t} \sum_{x=10}^{21} V_{x,t}^{a}$$

- Intervention:

$$V_{intervention}=\sum_{a} \sum_{t} \sum_{x=10}^{21} V_{x,t}^{a, int}$$

- Impact:

$$\Delta V=V_{baseline}-V_{intervention}$$

$\Delta V$ represents the number of violence victimisation events averted over the simulation period. Negative values at specific ages or time periods reflect temporal shifts in events across ages rather than increases in overall violence.

**References**

1. Together for Girls. Together for Girls, Resources. [cited 2024 Feb 28]. Kenya Violence Against Children and Youth Survey (VACS) report 2020. Available from: https://www.togetherforgirls.org/en/resources/kenya-vacs-report-2019

2. Cluver LD, Meinck F, Steinert JI, Shenderovich Y, Doubt J, Romero RH, et al. Parenting for Lifelong Health: a pragmatic cluster randomised controlled trial of a non-commercialised parenting programme for adolescents and their families in South Africa. BMJ Glob Health. 2018 Jan;3(1):e000539.

3. Cluver LD, Rudgard WE, Toska E, Zhou S, Campeau L, Shenderovich Y, et al. Violence prevention accelerators for children and adolescents in South Africa: A path analysis using two pooled cohorts. PLOS Med. 2020 Nov 9;17(11):e1003383.

4. Lachman J, Wamoyi J, Martin M, Han Q, Alfaro FAC, Mgunga S, et al. Reducing family and school-based violence at scale: a large-scale pre–post study of a parenting programme delivered to families with adolescent girls in Tanzania. BMJ Glob Health. 2024 Nov 24;9(11):e015472.

5. Drummond MF, Sculpher MJ, Claxton K, Stoddart GL, Torrance GW. Methods for the Economic Evaluation of Health Care Programmes [Internet]. Fourth Edition. Oxford University Press; 2015. Available from: https://books.google.co.uk/books?id=lvWACgAAQBAJ

6. Lachman JM, Alampay LP, Jocson RM, Alinea C, Madrid B, Ward C, et al. Effectiveness of a parenting programme to reduce violence in a cash transfer system in the Philippines: RCT with follow-up. Lancet Reg Health – West Pac. 2021;17:100279.

# **Text E. Summary of data extracted from the 2022 Kenya Demographic and Health Survey**

All estimates are specific to the nine targeted counties: Bungoma, Homa Bay, Kilifi, Mandera, Marsabit, Migori, Nairobi, Samburu, and Wajir.

**Table E1.** Kenya education estimates for 2021-2022 (current year) and 2020-2021 (previous year) for adolescents 10-18 years in the poorest wealth quintile

| **Grade** | **Grade specific enrolment rate (%)** | **Dropout rate (%)** | **Repetition rate (%)** | **Progression rate (%)** |
| --- | --- | --- | --- | --- |
| **Primary** |  |  |  |  |
| 1 | 0.02 | 0.00 | 0.06 | 0.94 |
| 2 | 0.08 | 0.00 | 0.05 | 0.95 |
| 3 | 0.13 | 0.00 | 0.07 | 0.93 |
| 4 | 0.15 | 0.01 | 0.07 | 0.92 |
| 5 | 0.13 | 0.01 | 0.06 | 0.93 |
| 6 | 0.14 | 0.02 | 0.05 | 0.93 |
| 7 | 0.11 | 0.04 | 0.05 | 0.91 |
| 8 | 0.10 | 0.07 | 0.12 | 0.81 |
| **Secondary** |  |  |  |  |
| 9 | 0.06 | 0.01 | 0.21 | 0.78 |
| 10 | 0.04 | 0.01 | 0.10 | 0.89 |
| 11 | 0.02 | 0.06 | 0.14 | 0.80 |
| 12 | 0.01 | - | 0.44 | - |

Note: All estimates are weighted using DHS sampling weights and restricted to adolescents in the poorest wealth quintile in the nine target counties. Grade-specific enrolment rates represent the proportion of adolescents aged 10–18 years enrolled in each grade in the current academic year. Dropout, repetition, and progression rates are calculated between the previous (2020–2021) and current (2021–2022) academic years. Progression refers to advancement to the next grade conditional on enrolment in the previous year. Rates may not sum exactly to 1 due to rounding. “–” indicates not applicable or insufficient data.

**Table E2.** Kenya education estimates for 2021-2022 (current year) and 2020-2021 (previous year) for adolescents 10-18 years in the poorest wealth quintile, girls

| **Grade** | **Grade specific enrolment rate (%)** | **Dropout rate (%)** | **Repetition rate (%)** | **Progression rate (%)** |
| --- | --- | --- | --- | --- |
| **Primary** |  |  |  |  |
| 1 | 0.02 | 0.01 | 0.04 | 0.95 |
| 2 | 0.07 | 0.00 | 0.06 | 0.94 |
| 3 | 0.11 | 0.01 | 0.06 | 0.93 |
| 4 | 0.14 | 0.00 | 0.08 | 0.92 |
| 5 | 0.13 | 0.00 | 0.06 | 0.94 |
| 6 | 0.15 | 0.02 | 0.03 | 0.95 |
| 7 | 0.12 | 0.04 | 0.05 | 0.91 |
| 8 | 0.11 | 0.10 | 0.07 | 0.83 |
| **Secondary** |  |  |  |  |
| 9 | 0.07 | 0.00 | 0.29 | 0.71 |
| 10 | 0.05 | 0.00 | 0.11 | 0.89 |
| 11 | 0.02 | 0.02 | 0.08 | 0.90 |
| 12 | 0.01 | - | 0.63 | - |

Note: All estimates are weighted using DHS sampling weights and restricted to adolescent girls in the poorest wealth quintile in the nine target counties. Grade-specific enrolment rates represent the proportion of adolescents aged 10–18 years enrolled in each grade in the current academic year. Dropout, repetition, and progression rates are calculated between the previous (2020–2021) and current (2021–2022) academic years. Progression refers to advancement to the next grade conditional on enrolment in the previous year. Rates may not sum exactly to 1 due to rounding. “–” indicates not applicable or insufficient data.

**Table E3.** Kenya education estimates for 2021-2022 (current year) and 2020-2021 (previous year) for adolescents 10-18 years in the poorest wealth quintile, boys

| **Grade** | **Grade specific enrolment rate (%)** | **Dropout rate (%)** | **Repetition rate (%)** | **Progression rate (%)** |
| --- | --- | --- | --- | --- |
| **Primary** |  |  |  |  |
| 1 | 0.03 | 0 | 0.07 | 0.93 |
| 2 | 0.08 | 0.01 | 0.04 | 0.95 |
| 3 | 0.14 | 0 | 0.07 | 0.93 |
| 4 | 0.15 | 0.01 | 0.06 | 0.93 |
| 5 | 0.14 | 0.02 | 0.05 | 0.93 |
| 6 | 0.12 | 0.01 | 0.08 | 0.91 |
| 7 | 0.11 | 0.03 | 0.06 | 0.91 |
| 8 | 0.10 | 0.06 | 0.16 | 0.78 |
| **Secondary** |  |  |  |  |
| 9 | 0.05 | 0.02 | 0.10 | 0.88 |
| 10 | 0.04 | 0.03 | 0.09 | 0.88 |
| 11 | 0.02 | 0.10 | 0.19 | 0.71 |
| 12 | 0.02 | - | 0.35 | - |

Note: All estimates are weighted using DHS sampling weights and restricted to adolescent boys in the poorest wealth quintile in the nine target counties. Grade-specific enrolment rates represent the proportion of adolescents aged 10–18 years enrolled in each grade in the current academic year. Dropout, repetition, and progression rates are calculated between the previous (2020–2021) and current (2021–2022) academic years. Progression refers to advancement to the next grade conditional on enrolment in the previous year. Rates may not sum exactly to 1 due to rounding. “–” indicates not applicable or insufficient data.

**Table E4.** Age-specific incidence of child marriage for adolescent girls 12-17 years in the poorest wealth quintile, based on 10-year recall.

| **Age** | **Total person-years at risk**  **(n)** | **Total incident marriage events**  **(n)** | **Age-specific incidence rate**  **(per 100 person years)** |
| --- | --- | --- | --- |
| **12** | 804 | 1.77 | 0.22 |
| **13** | 876 | 10.43 | 1.19 |
| **14** | 944 | 26.95 | 2.86 |
| **15** | 958 | 37.75 | 3.94 |
| **16** | 867 | 62.68 | 7.23 |
| **17** | 802 | 89.50 | 11.17 |
| **18** | 765 | 80.55 | 10.52 |

Note: Age-specific marriage rates were estimated from KDHS survey data using an adapted DHS-style rate calculation based on reported dates of first marriage. Estimates are weighted using DHS sampling weights and expressed per 100 person-years. Single-year age groups were used for ages 10–24, with a 10-year retrospective observation window preceding the survey. Event counts may be fractional due to weighting.

**Table E5.** Age-specific incidence of first pregnancy for adolescent girls 12-19 years in the poorest wealth quintile, based on 10-year recall.

| **Age** | **Total person-years at risk**  **(n)** | **Total incident pregnancy events**  **(n)** | **Age-specific incidence rate**  **(per 100 person years)** |
| --- | --- | --- | --- |
| **12** | 804 | 8.31 | 1.03 |
| **13** | 876 | 10.66 | 1.22 |
| **14** | 944 | 25.28 | 2.68 |
| **15** | 958 | 19.10 | 1.99 |
| **16** | 867 | 24.36 | 2.81 |
| **17** | 802 | 28.15 | 3.51 |
| **18** | 765 | 27.37 | 3.58 |
| **19** | 712 | 41.59 | 5.84 |

Note: Age-specific pregnancy incidence rates were estimated from KDHS survey data using a DHS-style rate calculation based on the contraceptive calendar. Estimates are weighted using DHS sampling weights and expressed per 100 person-years. Single-year age groups were used, with a 10-year retrospective observation window preceding the survey. Event counts may be fractional due to weighting.

**Table E6.** Age-specific incidence of second pregnancy for adolescent girls 12-19 years in the poorest wealth quintile, based on 10-year recall.

| **Age** | **Total person-years at risk**  **(n)** | **Total incident pregnancy events**  **(n)** | **Age-specific incidence rate**  **(per 100 person years)** |
| --- | --- | --- | --- |
| **15-19** | 183 | 21.48 | 11.71 |

Note: Age-specific second pregnancy incidence rates were estimated from KDHS survey data using an episode-based person-years calculation following the first observed pregnancy. Estimates are weighted using DHS sampling weights and expressed per 100 person-years. Due to limited sample size, rates were aggregated for ages 15–19. Event counts may be fractional due to weighting.

# Text F. Summary of data extracted from the 2019 Kenya Violence Against Children Survey

**Table F1**. Age-specific prevalence of violence victimisation in the last 12 months among adolescents and young adults 13-21 years

|  | **Girls and Young Women (N= 294)** | | | | **Boys and Young Men (N=145)** | | | |
| --- | --- | --- | --- | --- | --- | --- | --- | --- |
| **Age** | **n** | **Sexual violence, %** | **Physical violence, %** | **Emotional violence, %** | **n** | **Sexual violence, %** | **Physical violence, %** | **Emotional violence, %** |
| **13-15** | 435 | 13.2 | 21.1 | 10.8 | 233 | 3.1 | 19.2 | 6.6 |
| **16-18** | 368 | 16.0 | 11.7 | 13.0 | 248 | 1.9 | 11.0 | 6.1 |
| **19-21** | 264 | 16.7 | 4.7 | 8.7 | 177 | 11.8 | 7.9 | 5.0 |

Note: Estimates are based on VACS 2019 (Kenya) and represent past 12-month prevalence. Emotional and physical violence refer to violence perpetrated by parents, adult caregivers, or other adult relatives, while sexual violence includes unwanted sexual touching, unwanted attempted sex, physically forced sex, and sex pressured through harassment or threats, perpetrated by any individual. All estimates are weighted and nationally representative. Sample sizes (n) are unweighted.

# Text G. Methods and results for comprehensive review of evidence for the impact of cash and cash ‘plus’ on adolescent outcomes in Kenya

# Methods.

## Search strategy.

Our review of evidence for cash and cash ‘plus’ programmes in Kenya was conducted in three phases. First, we consulted experts at the Population Council’s GIRL Centre and the World Bank Kenya to identify evaluations of relevant cash ‘plus’ programmes. Second, we reviewed Cirillo et al.’s 2021 comprehensive review of government-implemented cash transfer programmes and adolescent outcomes across low- and middle-income countries, covering 85 studies from 26 countries published up to January 2019 (1). This review included experimental and quasi-experimental studies that reported adolescent-specific outcomes. Third, we conducted supplementary searches of PubMed and Google Scholar to identify any additional relevant studies.

## Study inclusion criteria.

Studies were included if they: (a) evaluated a poverty-targeted programme (e.g. means-tested) that provided cash transfers to caregivers of adolescents; (b) were conducted in Kenya; and (c) used a randomised or quasi-experimental design measuring adolescent outcomes (1).

## Overview of data items.

For each included study, we extracted the following data items using standardised Excel forms: author, year of publication, participant characteristics (age and gender), sample size, outcome indicators, effect sizes, p-values, measures of change, and follow-up duration.

# Results

## Search results.

Consultation with experts identified five studies of the Adolescent Girls Initiative–Kenya (AGI-K) intervention (2–6), including four peer-reviewed published papers (2–5), and one implementation-focused report on a county-scale expansion of AGI-K in Wajir (6).

From Cirillo et al., we identified eight studies in Kenya (7–14). However, only the four studies reporting education, pregnancy, or marriage outcomes were included in the evidence synthesis (7,9,10,13). All included studies evaluated the Cash Transfers for Orphans and Vulnerable Children Programme (CT-OVC). Of these, three were peer-reviewed published papers (7,9,10) and one was an impact evaluation (13).

No additional eligible studies were identified through PubMed or Google Scholar searches.

The included studies are summarised in Table G1, with detailed estimates of adolescent outcomes presented in Tables G2 and Table G3.

## Summary of study designs.

The AGI-K was a randomized controlled trial evaluating a multi-component cash ‘plus’ intervention to improve the well-being of adolescent girls in Kenya (2–5). The cash ‘plus’ intervention included community conversations, cash transfers conditional on school enrolment, life skills training, and financial literacy and savings classes. The evaluation was conducted in Kibera (urban Nairobi) and Wajir County (rural). Participants were randomly assigned to one of four intervention arms, which incrementally added components. Randomisation was conducted at the individual level in Kibera and at the cluster level in Wajir. Follow-up data were collected at 24 and 48 months in both sites, with an additional 72-month follow-up conducted in Wajir only. A subsequent evaluation assessed a county-wide scale-up in Wajir in 2021 (6).

The second intervention was Kenya’s Cash Transfer for Orphans and Vulnerable Children (CT-OVC) programme, introduced in 2004 with support from UNICEF and later integrated into the national budget in 2007. The programme targets ultra-poor households with orphans and vulnerable children and aims to improve child well-being and human capital outcomes. The CT-OVC was evaluated through a cluster randomised controlled trial across seven districts (including Kisumu, Migori, Homa Bay, Suba, Nairobi, Garissa, and Kwale), with households eligible if they were poor, contained at least one OVC, and were not receiving other cash transfers (13). The evaluation included three survey waves (baseline in 2007, with 24-month and 48-month follow-ups in 2009 and 2011), with sample sizes of approximately 1,500 treatment and 700 control households at baseline (9,10,13). Surveys collected detailed household socio-economic data, and the 2011 follow-up included additional modules on fertility (women aged 12–49) and youth outcomes (ages 15–24), including sexual behaviour, mental health, and HIV knowledge.

**Table G1.** Summary of cash ‘plus’ and cash transfer programmes evaluated for their effects on adolescent outcomes in Kenya

| Author (Year) | Programme Name | Aim | Study design | Age, years | Sample size |
| --- | --- | --- | --- | --- | --- |
| Austrian et al. (2021) | AGI-K | Empower economically disadvantaged adolescent girls by providing a comprehensive package of support | Cluster- and individual RCT | 11-14 girls | 4537 |
| Austrian et al. (2022) |  |  | Cluster-RCT | 13-16 girls | 2039 |
| Kangwana et al. (2022) |  |  | Individual RCT | 13-16 girls | 2075 |
| Austrian et al. (2024a) |  |  | Cluster-RCT | Mean = 18 girls | 2023 |
| Austrian et al. (2024b) | AGI-K Scale Up |  | Cluster-RCT | 11-14 girls | 1107 |
| Ward et al. (2010) | CT–OVC | Provide bi-monthly cash-transfers to households living with orphans and vulnerable children in Kenya | Cluster-RCT | 14-17 | 1018 girls and boys |
| Kenya CT-OVC Evaluation Team. (2012) |  |  | Cluster-RCT | >12 | 4175 girls and boys |
| Handa et al. (2015) |  |  | Cluster-RCT | 12-24 | 1547 girls |
| Handa et al. (2017) |  |  | Cluster-RCT | 15-25 | 552 girls / 877 boys |

Abbreviations: AGI-K, Adolescent Girls Initiative-Kenya; CT-OVC, Cash Transfer for Orphans and Vulnerable Children, RCT, Randomised Control Trial.

**Table G2.** Summary of effectiveness estimates from cash ‘plus’ programmes

| Author (Year) | Location | Population | Outcome | Effect size (95 CI) | P value | Unit | Period |
| --- | --- | --- | --- | --- | --- | --- | --- |
| Austrian et al. (2021) | Wajir | Girls | Enrolled in school | 0.10 | p<0.001 | pp | 24m |
|  | Nairobi | Girls | Enrolled in school | 0.01 | P<0.1 | pp | 24m |
| Austrian et al. (2022) | Wajir | Girls | Enrolled in school | 0.07 (0.02; 0.11) | p<0.01 | pp | 48m |
|  | Wajir | Girls | Ever married | -0.04 (-0.08; 0.00) | p<0.05 | pp |  |
|  | Wajir | Girls | Ever pregnant | -0.04 (-0.07; 0.00) | p<0.1 | pp |  |
|  | Wajir | Girls | Ever given birth | -0.02 (-0.05; 0.00) | p<0.1 | pp |  |
| Kangwana et al. (2022) | Nairobi | Girls | Enrolled in school | 0.03 (0.00; 0.07) | p<0.05 | pp | 48m |
|  | Nairobi | Girls | Ever pregnant | -0.01 (-0.04; 0.02) |  | pp |  |
|  | Nairobi | Girls | Ever given birth | -0.02 (-0.04; 0.01) | p<0.1 | pp |  |
| Austrian et al. (2024a) | Wajir | Girls | Enrolled in school | 0.07 (0.01; 0.13) | p<0.05 | pp | 72m |
|  | Wajir | Girls | Ever married | -0.06 (-0.12; 0.02) | p<0.05 | pp |  |
|  | Wajir | Girls | Ever pregnant | -0.04 (-0.09; 0.00) | p<0.1 | pp |  |
|  | Wajir | Girls | Ever given birth | -0.03 (-0.07; 0.00) | p<0.1 | pp |  |
| Austrian et al. (2024b) | Wajir | Out-of-school girls | School attendance 2022 | Arm 1: ref  Arm 2: 0.15  Arm 3: 0.16 | p<0.05  p<0.05 | pp | 16m |
|  | Wajir | Out-of-school girls | School attendance 2023 | Arm 1: ref  Arm 2: 0.19  Arm 3: 0.18 | p<0.05  p<0.05 | pp |  |

Estimates represent pooled treatment effects across intervention arms relative to the community engagement (violence prevention-only) control group.

Abbreviations: CI, confidence intervals; pp, percentage points.

**Table G3.** Summary of effectiveness estimates from cash transfer programmes

| Author (Year) | Population | Outcome | Effect size (95CI) | P value | Unit | Period |
| --- | --- | --- | --- | --- | --- | --- |
| Ward et al (2010) | Boys & girls | Ever attended school | 0.05 | p<0.05 | pp | 24m |
|  | Boys & girls | Enrolled in secondary school | 0.07 | p<0.05 | pp |  |
| Kenya CT-OVC Evaluation Team (2012) | Boys & girls | Returning to school | 0.02 (0.01; 0.04) | p<0.001 | pp | 24m |
|  | Boys & girls | Ever enrolled | 0.03 (0.00; 0.06) | p<0.05 | pp |  |
|  | Boys & girls | Enrolled in school | 0.08 (0.03; 0.12) | p<0.001 | pp |  |
| Handa et al. (2015) | Girls | First pregnancy | -0.05 (-0.09; -0.01) | p<0.05 | pp | 48m |
|  | Girls | Ever married | -0.001 (-0.01; 0.01) | p=0.83 | pp | 48m |
| Handa et al. (2017) | Boys & girls | Enrolled in school | 0.023 (-0.03, 0.08) | p=0.40 | pp | 48m |

Confidence intervals for Handa et al. (2017) were calculated from reported marginal effects and z-statistics.

Abbreviations: CES-D, Center for Epidemiologic Studies Depression Scale; CT-OVC, Cash Transfers for Orphans and Vulnerable Children; CI, confidence intervals; pp, percentage points.

## Quantitative synthesis of effect estimates.

We conducted meta-analyses to pool relative risks across studies for education enrolment, adolescent pregnancy, and child marriage. For each study, effect sizes were calculated from reported counts of events and sample sizes, and log-transformed relative risks and corresponding variances were calculated.

Given heterogeneity in study populations, intervention components, and settings (e.g. urban vs rural contexts), we used random-effects models estimated using restricted maximum likelihood (REML) to obtain pooled effect estimates. This approach allows the true effect size to vary across studies rather than assuming a common effect.

Where appropriate, we conducted analyses restricted to specific follow-up periods (e.g. 24- or 48-month follow-up) to avoid combining non-independent estimates from the same study across time.

*Education enrolment.* At 24 months post-baseline, the pooled estimate from the random-effects meta-analysis shows a relative risk of 1.07 (95% CI: 1.04, 1.09), indicating a statistically significant increase in the likelihood of adolescent school enrolment across the included studies, Figure G1. At 48 months post-baseline, the pooled estimate remains positive at 1.05 (95% CI: 1.02, 1.08), indicating that impacts are sustained over time, although potentially attenuated in magnitude, Figure G2. Effect sizes varied across contexts, with larger impacts observed in more disadvantaged settings.

*Adolescent pregnancy.* At 48 months post-baseline, the pooled estimate from the random-effects meta-analysis shows a relative risk of 0.83 (95% CI: 0.65, 1.07). This suggests a reduction in the risk of adolescent pregnancy, although the effect is not statistically significant, Figure G3.

*Child marriage.* At 48 months post-baseline, the pooled estimate from the random-effects meta-analysis shows a relative risk of 0.80 (95% CI: 0.57, 1.12). This indicates a reduction in the risk of child marriage, though the effect is not statistically significant, Figure G4.


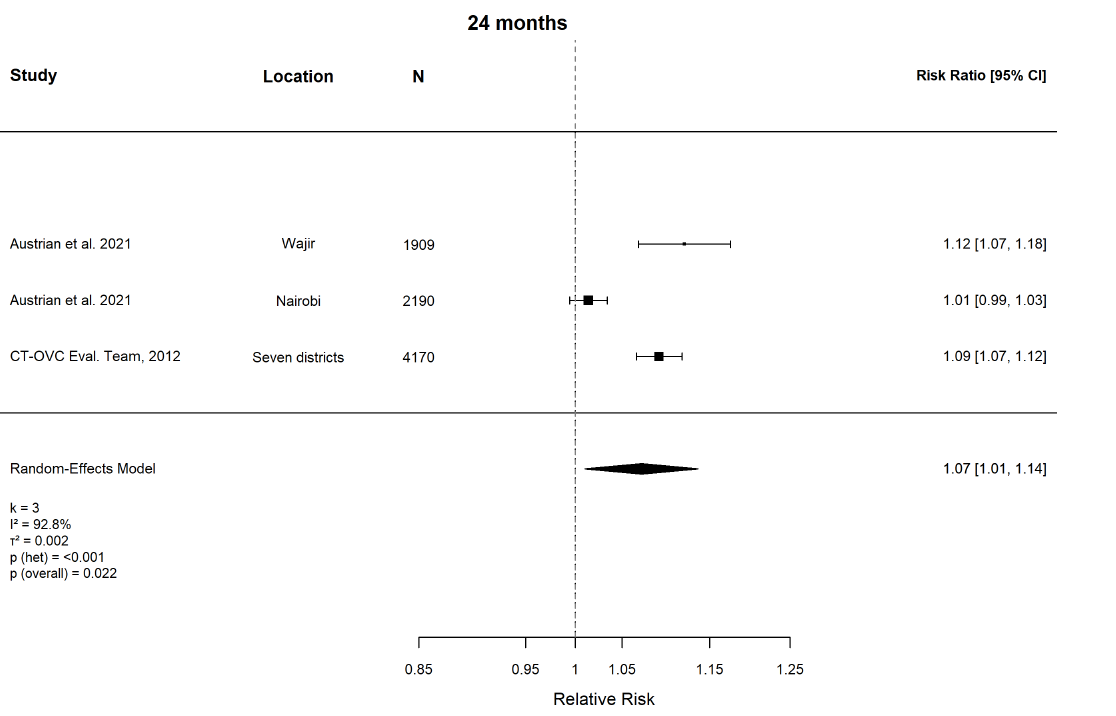


**Figure G1.** Forest plot showing study-specific and pooled relative risk estimates for school enrolment associated with cash and cash ‘plus’ interventions at 24 months post-baseline, based on a random-effects meta-analysis. Squares represent study-specific estimates and horizontal lines their 95% confidence intervals; the diamond represents the pooled estimate.


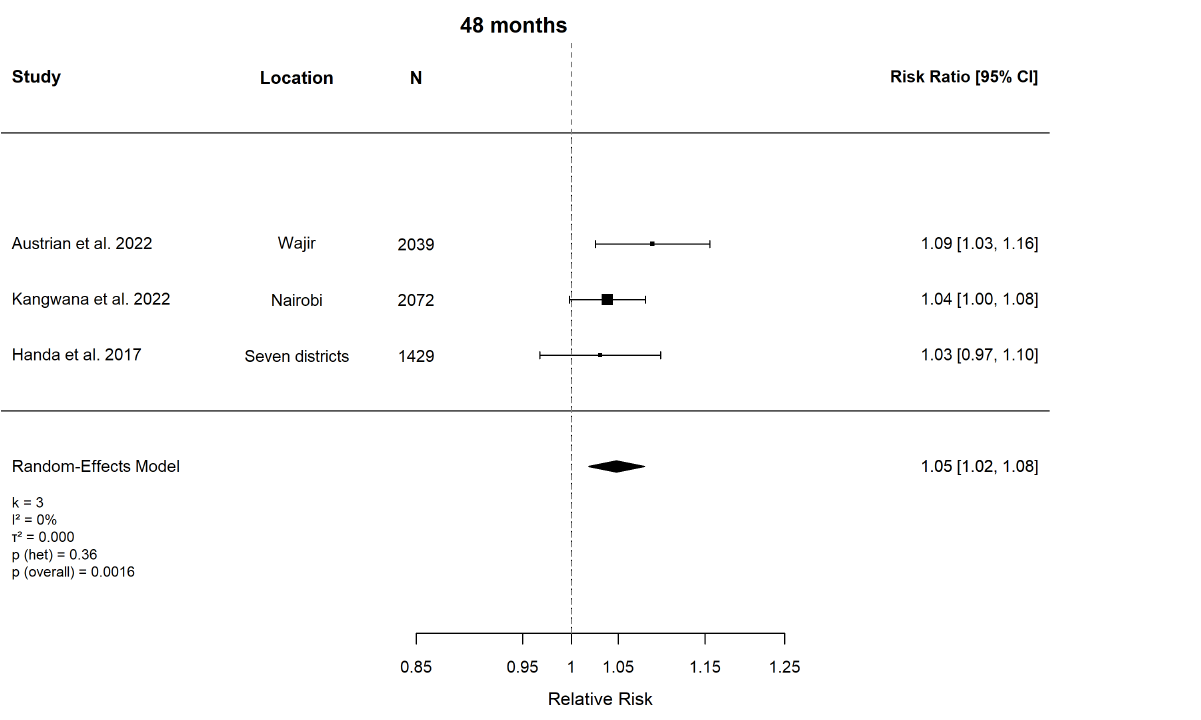


**Figure G2.** Forest plot showing study-specific and pooled relative risk estimates for school enrolment associated with cash and cash ‘plus’ interventions at 48 months post-baseline, based on a random-effects meta-analysis. Squares represent study-specific estimates and horizontal lines their 95% confidence intervals; the diamond represents the pooled estimate.


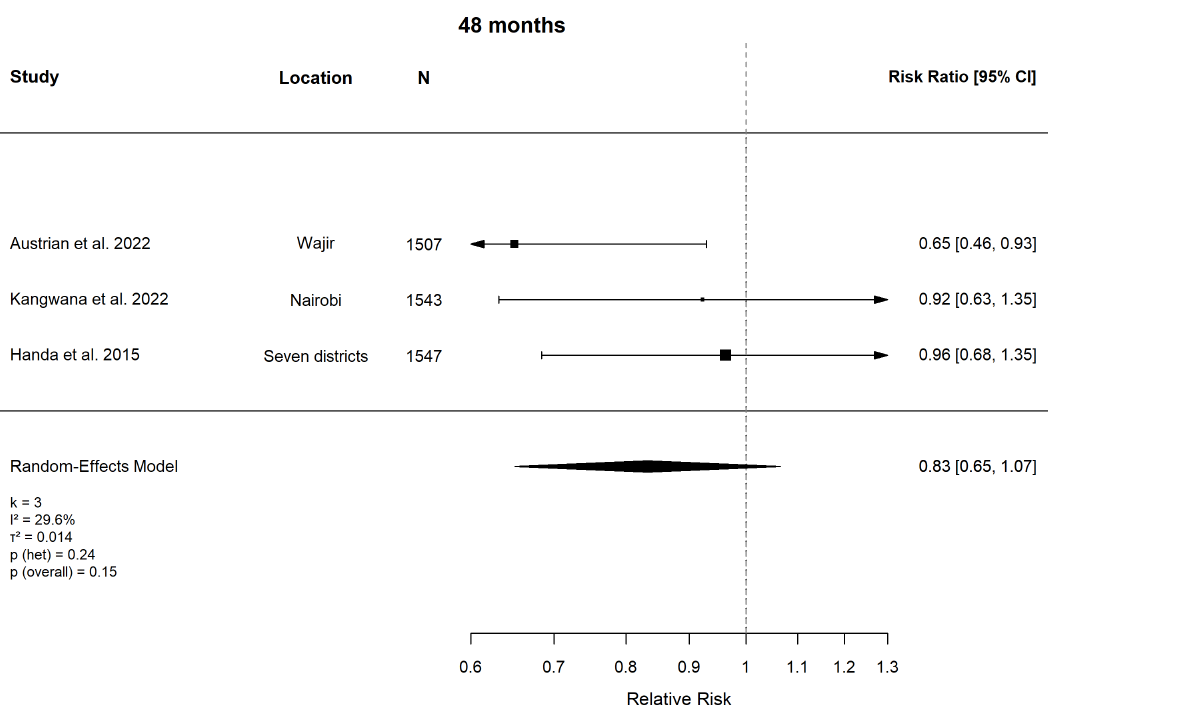


**Figure G3.** Forest plot showing study-specific and pooled relative risk estimates for adolescent pregnancy associated with cash and cash ‘plus’ interventions at 48 months post-baseline, based on a random-effects meta-analysis. Squares represent study-specific estimates and horizontal lines their 95% confidence intervals; the diamond represents the pooled estimate.


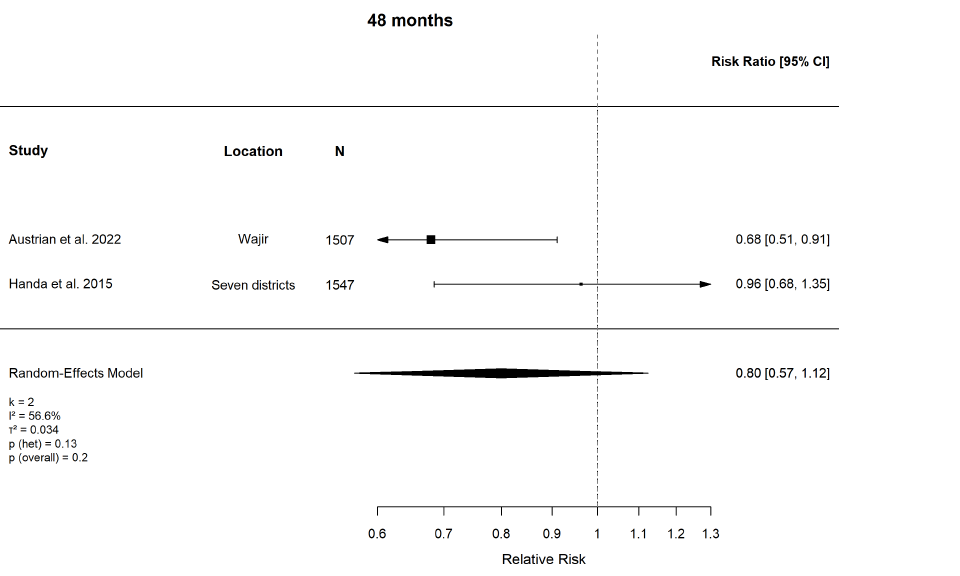


**Figure G4.** Forest plot showing study-specific and pooled relative risk estimates for child marriage associated with cash and cash ‘plus’ interventions at 48 months post-baseline, based on a random-effects meta-analysis. Squares represent study-specific estimates and horizontal lines their 95% confidence intervals; the diamond represents the pooled estimate.

# References

1. Cirillo C, Palermo T, Viola F. Non-contributory Social Protection and Adolescents in Lower- and Middle-Income Countries: A review of government programming and impacts. Florence: UNICEF Office of Research – Innocenti; 2021; Available from: https://www.unicef.org/innocenti/media/3831/file/UNICEF-Non-contributory-Social-Protection-2021.pdf

2. Austrian K, Soler-Hampejsek E, Kangwana B, Wado YD, Abuya B, Maluccio JA. Impacts of two-year multisectoral cash plus programs on young adolescent girls’ education, health and economic outcomes: Adolescent Girls Initiative-Kenya (AGI-K) randomized trial. BMC Public Health. 2021 Nov 24;21(1):2159.

3. Austrian K, Soler-Hampejsek E, Kangwana B, Maddox N, Diaw M, Wado YD, et al. Impacts of Multisectoral Cash Plus Programs on Marriage and Fertility After 4 Years in Pastoralist Kenya: A Randomized Trial. J Adolesc Health. 2022 Jun 1;70(6):885–94.

4. Kangwana B, Austrian K, Soler-Hampejsek E, Maddox N, Sapire RJ, Wado YD, et al. Impacts of multisectoral cash plus programs after four years in an urban informal settlement: Adolescent Girls Initiative-Kenya (AGI-K) randomized trial. PLOS ONE. 2022 Feb 7;17(2):e0262858.

5. Austrian K, Maluccio JA, Soler-Hampejsek E, Muluve E, Aden A, Wado YD, et al. Long-term impacts of a cash plus program on marriage, fertility, and education after six years in pastoralist Kenya: A cluster randomized trial. SSM - Popul Health. 2024;26:101663.

6. Austrian K, Muluve E, Nanjekho R, Maluccio J, Soler-Hampejsek E. Adolescent Girls Initiative–Kenya: Testing for Scale—Recommendations Report [Internet]. Nairobi: Population Council; 2024 Apr. Available from: https://knowledgecommons.popcouncil.org/focus_adolescents/119

7. Team TKCOE. The impact of Kenya’s Cash Transfer for Orphans and Vulnerable Children on human capital. J Dev Eff. 2012;4(1):38–49.

8. Handa S, Halpern CT, Pettifor A, Thirumurthy H. The government of Kenya’s cash transfer program reduces the risk of sexual debut among young people age 15-25. PloS One. 2014;9(1):1. doi:10.1371/journal.pone.0085473

9. Handa S, Peterman A, Huang C, Halpern C, Pettifor A, Thirumurthy H. Impact of the Kenya Cash Transfer for Orphans and Vulnerable Children on early pregnancy and marriage of adolescent girls. Soc Sci Med 1982. 2015;141:36–45.

10. Handa S, Palermo T, Rosenberg M, Pettifor A, Halpern CT, Thirumurthy H. How does a national poverty programme influence sexual debut among Kenyan adolescents? Glob Public Health. 2017;12(5):5. doi:10.1080/17441692.2015.1134617

11. Asfaw S, Davis B, Dewbre J, Handa S, Winters P. Cash Transfer Programme, Productive Activities and Labour Supply: Evidence from a Randomised Experiment in Kenya. J Dev Stud. 2014;50(8):8. doi:10.1080/00220388.2014.919383

12. Kilburn K, Thirumurthy H, Halpern CT, Pettifor A, Handa S. Effects of a Large-Scale Unconditional Cash Transfer Program on Mental Health Outcomes of Young People in Kenya. J Adolesc Health. 2016;58(2):2. doi:10.1016/j.jadohealth.2015.09.023

13. Ward P, Hurrell A, Visram A, Riemenschneider N, O’Brien C, MacAuslan I, et al. CASH TRANSFER PROGRAMME FOR ORPHANS AND VULNERABLE CHILDREN (CT-OVC), KENYA OPERATIONAL AND IMPACT EVALUATION, 2007–2009. 2010.

14. Merttens F, Hurrell A, Marzi M, Attah R, Farhat M, Kardan A, et al. Kenya Hunger Safety Net Programme Monitoring and Evaluation Component. 2009

# Text H. Methods and results for comprehensive review of evidence for the impact of parenting support interventions on adolescent outcomes.

# Methods.

## Search strategy.

We reviewed the World Health Organization’s (WHO’s) global systematic review on the impact of parenting support programmes on child maltreatment and other secondary outcomes published in 2021 (1). Our primary outcomes of interest were emotional, physical and sexual violence victimisation.

The WHO review represents the most comprehensive and up-to-date synthesis of evidence on parenting support programmes globally. It included all randomised controlled trials (RCTs) published up to December 2020, covering 131 studies across 65 countries (1).

One sub-review focused specifically on parenting programmes targeting adolescents aged 10–17 years in low- and middle-income countries (LMICs), extracting evidence from 30 RCTs across 16 countries (1). We explored the feasibility of using these pooled estimates; however, only two studies contributed to the meta-analyses of physical and psychological abuse, and none were conducted in Eastern or Southern Africa (1).

Given this limitation, we conducted a targeted review of all studies identified in the WHO review that met our inclusion criteria. We supplemented this with backward and forward snowballing and included relevant grey literature and programme evaluations identified through expert consultation.

## Study inclusion criteria.

We applied the following criteria for including studies in our evidence synthesis, studies must be a) focused on adolescents; b) be conducted in Eastern or Southern Africa; and c) include emotional and physical victimisation as an outcome, as these were the primary violence outcomes of interest to us.

## Overview review of data items.

From our included studies, we extracted the following data items into data extraction forms in Microsoft Excel: first author, year of publication, study country, title/name of the intervention, aim of the intervention, study sample size, age and gender of study participants, duration of follow-up, outcome indicator(s), effect size, and standard errors.

## Key informant interviews.

We conducted key informant interviews with Dr. Jamie Lachman and Dr. Jenny Doubt, who have extensive experience in the design and implementation of parenting interventions in Africa. Their insights informed study selection, interpretation of results, and identification of relevant unpublished and grey literature.

# Results

## Search results.

From the WHO global review of 131 studies across 65 countries, we identified seven studies conducted in Eastern and Southern Africa (2–8). However, only two met our inclusion criteria: i) the Furaha Teen uncontrolled pre-post evaluation in Tanzania; and ii) the Sinovuyo Teen cluster-RCT in South Africa (2,10). A summary of the two evaluations is provided in Table H1, and a full range of the measured effects of the two interventions is provided in Table H2.

**Table H1.** Summary of included studies.

| Author (Year) | Country | Intervention | Study design | Follow-up | Age, years | Sample size |
| --- | --- | --- | --- | --- | --- | --- |
| Cluver et al (2018) | South Africa | Sinovuyo Teen | Cluster RCT | 1-9months post intervention | 10-18 | 552 families |
| Lachman et al (2024) | Tanzania | Furaha Teen | Uncontrolled pre-post-study | Endline | 10-14 girls | 27 319 parent-child dyads |

Abbreviations: RCT, randomised controlled trial. Sinovuyo in isiXhosa translates to ‘We have Joy’, and Furaha in Swahili translates to ‘Joy’.

## Summary of study designs.

Both Sinovuyo Teen and Furaha Teen are part of the Parenting for Lifelong Health (PLH) suite of interventions, which aim to reduce violence against children and adolescents by improving parenting practices. These programmes consist of structured, group-based parenting interventions delivered over approximately 14 sessions to adolescents and their caregivers. Both interventions at targeted to at-risk families living with adolescent. The core contents of these sessions cover a range of topics including: praise and relationship building, managing stress and anger, family problem-solving, planning together to protect adolescents from community violence, monthly family budgeting, and saving and responding to crises.

The Sinovuyo Teen programme was implemented in the Eastern Cape province of South Africa and evaluated using a cluster randomised controlled trial design (2). The study included adolescents aged 10–18 years and their caregivers across 40 communities, which were randomised into treatment and control groups. The intervention was delivered through community-based group workshops, supplemented with home visits for participants who missed sessions.

The Furaha Teen programme targeted adolescent girls aged 10–14 years and their caregivers and was delivered at scale across eight districts in Tanzania (10). The evaluation, known as the Furaha Adolescent Implementation Research (FAIR) study, used an uncontrolled pre–post design.

The programme reached over 75,000 beneficiaries, including 27,319 parent–adolescent dyads included in the quantitative analysis. The evaluation employed multilevel regression models to estimate programme effects on multiple outcomes related to parenting practices and child maltreatment.

**Interpretation**

Across both studies, parenting support interventions were associated with substantial reductions in physical and emotional violence, particularly in the Furaha Teen evaluation, which demonstrated large and statistically significant effects across multiple outcomes.

However, the evidence base is limited to two studies, and findings are heterogeneous across contexts and study designs. The strongest effects were observed in large-scale implementation settings, while smaller trials showed more variable results.

These findings should therefore be interpreted with caution, but suggest that parenting support interventions may be a promising approach for reducing adolescent violence in Eastern and Southern Africa.

**Table H2.** Effect estimates for primary violence outcomes from the Furaha Teen trial, Tanzania and Sinovuyo Teen trial, South Africa

| Author (Year) | Outcomes | Effect size | |
| --- | --- | --- | --- |
| Caregiver report | | Beta (SE) | IRR (95% CI) |
| Lachman et al. (2024) | Overall maltreatment | −0.60 (0.01) | 0.55 (0.54-0.56) |
|  | Physical abuse | −0.66 (0.01) | 0.51 (0.5-0.53) |
|  | Psychological abuse | −0.58 (0.01) | 0.56 (0.55-0.57) |
|  | School violence victimisation | −0.25 (0.02) | 0.78 (0.74-0.81) |
|  | Positive parent involvement | 0.01 (0.03) | 1.03 (0.98-1.09) |
|  | Poor parent supervision | −1.21 (0.05) |  |
|  | Parent support of education | −0.50 (0.02) |  |
|  | Child depression | −0.72 (0.04) |  |
|  | Child emotional problems | −0.94 (0.02) |  |
|  | Child conduct problems | −1.44 (0.02) |  |
|  | Sexual health communication | −0.55 (0.01) |  |
|  | Child conduct problems | −0.22 (0.01) |  |
|  | Sexual health communication§§ | 1.62 (0.02) |  |
| Cluver et al. (2018) | Physical and emotional abuse |  | 0.55 (0.4-0.75) |
|  | Depression | -3.72 (1.26) |  |
|  | Alcohol and substance use |  | 0.67 (0.49-0.99) |
|  | Adolescent externalising behaviour | -1.86 (1.06) |  |
|  | Positive parenting | 1.18 (0.52) |  |
|  | Involved parenting | 2.80 (0.83) |  |
|  | Poor parent supervision | 4.37 (0.80) |  |
|  | Household economic hardship | -3.83 (0.69) |  |
|  | Planning for risk avoidance | 1.41 (0.36) |  |
| Adolescent report | | Beta (SE) | IRR (95% CI) |
| Lachman et al. (2024) | Overall maltreatment | −0.57 (0.01) | 0.57 (0.56-0.58) |
|  | Physical abuse | −0.58 (0.01) | 0.56 (0.55-0.58) |
|  | Psychological abuse | −0.60 (0.01) | 0.55 (0.54-0.56) |
|  | School violence victimisation | −0.17 (0.02) | 0.84 (0.82-0.86) |
|  | Positive parent involvement | −0.82 (0.03) | −0.82 (1.03-0.98) |
|  | Poor parent supervision | −0.63 (0.05) |  |
|  | Parent support of education | −0.50 (0.02) |  |
|  | Child depression | −0.43 (0.04) |  |
|  | Child emotional problems | 0.02 (0.02) |  |
|  | Child conduct problems | −0.17 (0.02) |  |
|  | Sexual health communication | 1.77 (0.01) |  |
| Cluver et al. (2018) | Physical and emotional abuse |  | 0.90 (0.65-1.24) |
|  | Depression and suicidality |  | 1.02 (0.77-1.35) |
|  | Alcohol and substance abuse |  | 0.55 (0.33-0.93) |
|  | Adolescent externalising behaviour | 0.97 (0.82) |  |
|  | Positive parenting | 0.59 (0.57) |  |
|  | Involved parenting | 2.54 (0.92) |  |
|  | Poor parent supervision | -2.42 (0.78) |  |
|  | Household economic hardship | -1.87 (0.78) |  |
|  | Planning for risk avoidance | 0.78 (0.33) |  |

Abbreviations: SE, standard error; IRR, incidence rate ratio; IPV, Intimate Partner Violence; CI, confidence interval.

# References

1. Backhaus S, Schafer M, Melendez-Torres GJ, Knerr W, Lachman JM. World Health Organization Guidelines on Parenting Interventions to Prevent Maltreatment and Enhance Parent–Child Relationships with Children aged 0-17 Years: Report of the Systematic Reviews of Evidence.

2. Cluver LD, Meinck F, Steinert JI, Shenderovich Y, Doubt JS, Herrero Romero R, et al. Parenting for Lifelong Health: A pragmatic cluster randomised controlled trial of a non-commercialised parenting programme for adolescents and their families in South Africa. 2018;

3. Puffer ES, Green EP, Sikkema KJ, Broverman SA, Ogwang-Odhiambo RA, Pian J. A church-based intervention for families to promote mental health and prevent HIV among adolescents in rural Kenya: Results of a randomized trial. J Consult Clin Psychol. 2016 Jun;84(6):511–25.

4. Betancourt TS, Ng LC, Kirk CM, Brennan RT, Beardslee WR, Stulac S, et al. Family-based promotion of mental health in children affected by HIV: a pilot randomized controlled trial. J Child Psychol Psychiatry. 2017 Aug;58(8):922–30.

5. Bell CC, Bhana A, Petersen I, McKay MM, Gibbons R, Bannon W, et al. Building Protective Factors to Offset Sexually Risky Behaviors among Black Youths. J Natl Med Assoc. 2008 Aug;100(8):936–44.

6. Bogart LM, Skinner D, Thurston IB, Toefy Y, Klein DJ, Hu CH, et al. Let’s Talk!, A South African Worksite-Based HIV Prevention Parenting Program. J Adolesc Health Off Publ Soc Adolesc Med. 2013 Nov;53(5):602–8.

7. Armistead L, Cook S, Skinner D, Toefy Y, Anthony ER, Zimmerman L, et al. Preliminary results from a family-based HIV prevention intervention for South African youth. Health Psychol Off J Div Health Psychol Am Psychol Assoc. 2014 Jul;33(7):668–76.

8. Bhana A, Mellins CA, Petersen I, Alicea S, Myeza N, Holst H, et al. The VUKA Family Program: Piloting a family-based psychosocial intervention to promote health and mental health among HIV infected early adolescents in South Africa. AIDS Care. 2014 Jan;26(1):10.1080/09540121.2013.806770.

9. Lachman J, Wamoyi J, Martin M, Han Q, Alfaro FAC, Mgunga S, et al. Reducing family and school-based violence at scale: a large-scale pre–post study of a parenting programme delivered to families with adolescent girls in Tanzania. BMJ Glob Health [Internet]. 2024 Nov 24 [cited 2025 Mar 3];9(11). Available from: https://gh.bmj.com/content/9/11/e015472

10. Wamoyi J, Lachman J, Martin M, Shenderovich Y, Manjengenja N, Ndyetabura E, et al. The Furaha Adolescent Implementation Research (FAIR) Study: Final Report. 2022.

11. Parenting for Lifelong Health [Internet]. [cited 2024 Apr 12]. Available from: <https://www.who.int/teams/social-determinants-of-health/parenting-for-lifelong-health>

**Text I.** **Sensitivity of key impacts to persistence assumptions.**

We assessed the sensitivity of estimated programme impacts to assumptions regarding the duration over which effects on education, fertility, and marriage persist after programme completion. The main analysis assumes impacts persist for 2 years following cessation of transfers, with partial annual decay.

Here, we vary the duration of persistence (1, 2, 4 years) while holding all other model parameters constant. Results should be interpreted as cumulative impacts under alternative persistence assumptions. Across all outcomes, estimated impacts increase monotonically with longer persistence durations, with no change in the direction of effects.

Uncertainty intervals are not presented for these scenarios as the analysis isolates structural assumptions regarding persistence; uncertainty in underlying effect sizes is presented in the main results.

**Table I1.** Education and associated economic impacts under alternative persistence assumptions

| Persistence | Years of schooling gained | % change | Labour earnings gained from increased schooling, US$ millions | Relative to baseline |
| --- | --- | --- | --- | --- |
| 1 year | 65,100 | 6 | 277 | 0.87x |
| 2 years | 74,900 | 7 | 318 | 1.00x |
| 4 years | 93,600 | 9 | 396 | 1.25x |

Abbreviations: US, United States.

Notes: Relative to baseline values under the 2-year persistence assumption. All other model parameters are held constant.

**Table I2.** Fertility and marriage impacts

| Persistence | Child marriages averted | % change | Relative to baseline | Pregnancies averted | % change | Relative to baseline |
| --- | --- | --- | --- | --- | --- | --- |
| 1 year | 1250 | 9 | 0.83x | 1140 | 7 | 0.80x |
| 2 years | 1500 | 11 | 1.00x | 1420 | 8 | 1.00x |
| 4 years | 2000 | 14 | 1.33x | 1930 | 11 | 1.36x |

Notes: Relative to baseline values under the 2-year persistence assumption. All other model parameters are held constant.
