# Supplementary material for: Estimating the human development impacts and economic returns from an adolescent cash ‘plus’ programme in Kenya: An economic modelling study
Source: PLOS Glob Public Health. 2026 Jul 24;6(7):e0006822. doi: 10.1371/journal.pgph.0006822 (PMC13399278; doi:10.1371/journal.pgph.0006822)
Supplement: Attachment — Submitted filename: Response to reviewers comments.docx [file pgph.0006822.s003.docx]

**Reviewer #1:**

**Review of “Estimating the human development impacts and economic returns from an adolescent cash ‘plus’ programme in Kenya: An economic modelling study” submitted to PLOS Global Public Health (PGPH-D-25-2492). The article, presumably based on a comprehensive report or feasibility study, represents a thorough example of approximating the economic impacts of scaling up a cash transfer plus program in Kenya. Impact studies alone too often provide only estimated effects on a selected set of important outcomes, occasionally presenting cost-effectiveness but only rarely providing insights into actual economic returns or cost-benefit (with benefits measured here as the returns to increased human capital in the labor market). As an example of the latter, this study can serve as a model guide for other similar exercises. Important in its approach is indicating some sensitivity around the mid-case estimates, as there are many underlying assumptions. The concluding discussion makes clear some of the potential weaknesses, including possible understatement of benefits suggesting findings might be conservative.**

**Response:** Thank you for this thoughtful assessment. We are grateful for your positive appraisal of the study, and your comments have helped to significantly strengthen the manuscript.

In response to your observation regarding uncertainty around mid-case estimates, we have strengthened the manuscript by expanding the sensitivity analyses. Specifically, we conducted a probabilistic sensitivity analysis of the economic returns component, incorporating uncertainty in both labour market parameters and programme effectiveness (including schooling gains), alongside a deterministic one-way sensitivity analysis presented as a tornado plot. These additions improve transparency around key assumptions, explicitly characterise the distribution of possible outcomes, and strengthen the assessment of robustness. These analyses are now described in the Methods section and presented in Figure 2.

**1. p. 2 – The tables containing program impacts do not strongly suggest to me that the cash plus is greater than cash for this Kenyan program, but I may be missing something (Table 2 in supplementary materials).**

**Response:** We agree that Table 2 does not uniformly show larger point estimates for cash ‘plus’ interventions relative to cash-only programmes across all outcomes. The manuscript has been revised to clarify this interpretation and to avoid implying consistent superiority in effect sizes.

Our intention is not to claim consistent superiority of cash ‘plus’ interventions in effect size, but rather to highlight their broader multidimensional scope. While effect sizes on specific indicators may be comparable, cash ‘plus’ interventions are structured to address multiple domains simultaneously, including education, fertility-related outcomes, gender norms, skills, and economic participation. The revised text clarifies this distinction.

We also agree that the modelled outcomes may underestimate the full range of impacts, particularly for domains such as adolescent agency, which are difficult to quantify and are not fully captured in the current analysis. This has been clarified in the Discussion.

**2. p. 3 I was unfamiliar with the term “decision analytic-economic modelling” as a specific approach, and suggest it be defined (sorry if I missed this).**

**Response:** Thank you for this comment. We agree that the term “decision-analytic modelling,” while commonly used in health economics to describe modelling approaches that synthesise evidence to inform policy decisions, may not be widely recognised across all audiences. To improve clarity and align with more commonly used terminology, we have replaced this with “model-based economic evaluation” throughout the manuscript.

The Methods section now more clearly describes the model structure. Specifically, we use a suite of linked models, including age–period–cohort simulations implemented within a Lexis framework to model population dynamics, and grade–period–cohort simulations to capture educational progression. Outputs from these components are synthesised within an economic evaluation framework to estimate long-term costs and returns.

***Methods:*** *“We developed deterministic simulation models in Microsoft Excel to estimate the projected impacts of the adolescent cash ‘plus’ programme. Education impacts were modelled using a grade-structured cohort transition model based on the UNESCO Educational Simulation Model (ESM) (55,56). This model tracks multiple school-grade cohorts over annual time steps. Its baseline state is defined by age- and grade-specific enrolment rates, and its evolution is determined by grade-specific progression rates, with students able to advance by a maximum of one grade per year.*

*Health and protection outcomes (adolescent pregnancy, child marriage, and violence victimisation) were modelled using age-structured cohort models based on Lexis modelling principles (57). These models simulate multiple age cohorts advancing in annual time steps, with each cohort ageing one year per cycle. Cohorts enter at programme-eligible ages (10–18 years) and are followed over time as they age through the relevant risk periods (e.g., up to age 20 for adolescent pregnancy). Incidence of pregnancy, marriage, and violence was determined by age-specific incidence rates (58).*

*The full model framework, including the education and health transition models, analytical code used to derive effect estimates and parameters, and supporting documentation, is available via the Open Science Framework (OSF) (59).”*

**3. p. 3 full range of potential outcomes – yes, though in practice and in this study that is not feasible. Perhaps a fuller range?**

**Response:** We agree that capturing the full range of outcomes is not feasible, and we have amended the text to refer to a *fuller* range of outcomes.

***Background:*** *“Because these programmes generate broad and interrelated effects, economic evaluations must capture a fuller range of potential human development outcomes than traditional cost analyses typically consider.”*

**4. p. 3 Indicates plans to implement the program in 2025. Since it is now late in the year, has this been done or dates modified?**

**Response:** Although Cabinet and Treasury approval have been secured, implementation has been delayed. The manuscript has been revised to remove references to specific implementation dates and to retain the framing of the analysis as a policy-informing tool.

***Background:*** *“Building on this evidence base, the Government of Kenya has developed a national adolescent-focused programme aimed at improving school progression and delaying adolescent pregnancy (40,50). The programme was designed in 2024-25 through a multi-stakeholder technical working group led by the State Department of Social Protection’s Directorate of Child Services in collaboration with development partners. As part of this policy design process, the aim of this study was to estimate the projected impact, economic returns, and cost-effectiveness of the proposed programme over a four-year implementation period.”*

**5. p. 6 – A provider perspective for costs and a beneficiary perspective for outcomes/benefits seems appropriate. It was nice to see that additional teacher costs, for example, were taken into consideration. But this approach does exclude private costs which for some could be important (and may also be heterogeneous).**

**Response:** We agree that adopting a provider perspective for costs and a beneficiary perspective for outcomes is appropriate for the objectives of this analysis. However, as you note, this approach excludes private costs borne by participants and their households, which may be important and heterogeneous across subgroups.

Because this study relied on secondary data and modelling rather than primary data collection, we were not able to capture these private costs directly. We have therefore added a paragraph to the Limitations section acknowledging this exclusion and discussing the potential implications for interpretation.

The revised manuscript also clarifies that, in this context, private costs to participants are likely to be small relative to total programme costs incurred by providers. We emphasize that this does not imply that such costs are unimportant, particularly for vulnerable households, but rather that their exclusion is unlikely to substantially affect the overall cost–benefit estimates from the provider perspective adopted in this study.

***Discussion:*** *“Second, because this study relied on secondary data and modelling rather than primary data collection, we were not able to capture private costs borne by participants and their households directly. These are therefore not included, and results reflect a provider perspective. The direction of these costs is likely to be mixed, with increased school participation raising short-term household expenditures and reductions in adolescent pregnancy and early marriage lowering costs. Returns may therefore be overstated or understated depending on the balance of these effects.”*

**6. How sensitive are the findings to alternative discount rates, if some were considered?**

**Response:** Sensitivity to alternative discount rates and other key parameters was assessed through probabilistic and deterministic sensitivity analyses.

In the probabilistic sensitivity analysis, which jointly varies macroeconomic and labour market parameters as well as programme effectiveness (including schooling gains), the mean present value of total wage returns was US$174 million (SD = US$104 million), with a median of US$150 million (Figure 2a). The 95% uncertainty interval ranged from US$49 million to US$441 million. The probability that the total incremental present value exceeded the programme cost of US$46.2 million was 0.98, while the probability of exceeding twice programme costs was 0.81.

Deterministic one-way sensitivity analysis indicated that results were most sensitive to assumptions regarding the discount rate, the Mincer return to schooling, and real wage growth, with schooling gains also contributing meaningfully to overall uncertainty.

Descriptions of these methodological approaches have been added to the Methods section, with corresponding results and interpretation presented in the Results and Discussion sections. Taken together, these analyses indicate that while the magnitude of estimated returns varies with alternative assumptions, the conclusion that the programme generates substantial positive economic returns remains robust.

***Methods:*** “*We conducted a probabilistic sensitivity analysis (PSA) with 20,000 Monte Carlo draws (58). Parameter uncertainty was sampled from specified distributions (truncated normal, lognormal, triangular, uniform), Table 2. Present value estimates of lifetime earnings gains were calculated for each draw.*

*We also performed one-way deterministic sensitivity analyses, varying individual parameters across their respective uncertainty ranges while holding others constant. Results were summarised using a tornado diagram.”*

***Results:*** *“In probabilistic sensitivity analysis, the mean present value of total wage returns was US$174 million (SD = US$104 million), with a median of US$150 million, Figure 2. The 95% uncertainty interval ranged from US$49 million to US$441 million. The probability that the total incremental present value exceeded the programme cost of US$46.2 million was 0.98, while the probability of exceeding twice the programme cost was 0.81. Deterministic one-way sensitivity analysis indicated that results were most sensitive to assumptions about the discount rate, mincer return, and real wage growth with comparatively smaller influence from other parameters, including baseline wage levels, schooling gains, and gender-related parameters, Figure 2.”*

**7. I did not notice explicit discussion of the return to education in the labor market results, but possibly missed them in the appendix? If not, a brief description of that may be useful, 40 years is a long time period.**

**Response:** Thank you for this helpful point. The Results section has been revised to more explicitly describe how the return to education was modelled over beneficiaries’ working lives. Lifetime economic returns are now clearly described as being estimated by applying a Mincerian return to each additional year of schooling to projected baseline earnings, with returns accruing over a 40-year working life and discounted to present value.

Given the long time horizon, sensitivity analyses around key labour market assumptions, including returns to schooling, wage growth, discount rates, and gender-related earnings parameters, have also been expanded. While absolute net present values vary under alternative assumptions, the overall conclusions remain robust.

***Results:*** *“Lifetime economic returns were estimated by applying an assumed Mincerian return to each additional year of schooling to projected baseline earnings, with returns accruing over a 40-year working life and discounted to present value. The projected economic return from increased schooling is estimated at US$318 million in present value terms. High- and low-range estimates are reported in Table 5.”*

**Reviewer #2:**

**Review report on manuscript: Estimating the human development impacts and economic returns from an adolescent cash ‘plus’ programme in Kenya: An economic modelling study**

**Summary**

**The manuscript models the human-development impacts, economic returns, and cost-effectiveness of an adolescent cash “plus” package in nine Kenyan counties (2026-2029). Components include conditional cash transfers (CCTs) tied to school attendance, adolescent clubs, community conversations, parenting support (PLH-Teen), and case management (modelled qualitatively). The model projects sizeable gains in years of schooling and reductions in adolescent pregnancy, child marriage, and violence, at ~US$42.5m over four years, with an ROI of ~US$246m in future labour productivity and favourable CERs relative to several education comparators.**

**Overall, the paper addresses a timely policy question with an ambitious cross-sector modelling framework. However, in its current form the economic methods, parameter provenance, and sensitivity analysis communication need revising before the paper gets published and results inform policy decisions.**

**Response:** We appreciate this careful and constructive summary. We agree that clearer presentation of the economic methods, parameter provenance, and uncertainty analysis is essential for policy relevance.

In response, we have restructured the manuscript to align more closely with the CHEERS reporting checklist, with improved reporting of the analytic perspective, time horizon, model structure, parameter sources, and costing approach. We have also strengthened the Results section to more clearly present base-case findings alongside uncertainty analyses.

We added both probabilistic and deterministic sensitivity analyses in the revision. The probabilistic sensitivity analysis jointly varies key demographic, programme-effectiveness (including schooling gains), labour market, and macroeconomic parameters, and reports uncertainty intervals and the probability that benefits exceed costs. We also added a deterministic one-way sensitivity analysis, presented as a tornado plot, to identify the parameters driving results. These additions strengthen the assessment of uncertainty and robustness.

To support reproducibility, we have made the full model framework, code, and supporting materials publicly available via the Open Science Framework (OSF), with the link provided in the manuscript.

Together, these revisions strengthen the clarity and policy relevance of the study.

**1. Transparency & reproducibility of the model: The study rests on multiple linked Excel models (education transition; Lexis age-cohort models; earnings projections). Please release the full code/model files and a runbook (inputs → transformations → outputs), plus a parameter dictionary mapping each input to a source and a baseline value/CI. PLOS requires open data/code “sufficient to reproduce the findings”; the current statement (“all relevant data are within the paper/SI”) is not enough for a decision-analytic model.**

**Response:** Thank you for this comment. The modelling framework consists of multiple linked Excel-based models, including education transitions, Lexis age–cohort simulations, and earnings projections. In response, we have created a public Open Science Framework repository containing the model files and the syntax needed to reproduce the analyses: <https://doi.org/10.17605/OSF.IO/62B53>. We have also updated the data availability statement and expanded the methodological description and parameter documentation in the manuscript and supplementary materials to improve transparency and reproducibility.

***Methods:*** *“The full model framework, including the education and health transition models, analytical code used to derive effect estimates and parameters, and supporting documentation, is available via the Open Science Framework (OSF) (59).”*

**2. Effect‐size synthesis and external validity: Several effects are pulled from AGI-K and other evaluations, with some meta-analytic pooling across Nairobi and Wajir and carry-over to mixed-sex beneficiaries. That’s defensible but show: (i) inclusion criteria; (ii) pooling method; (iii) between-site heterogeneity; (iv) why effects for girls can be applied to boys (or provide sex-specific sensitivity bounds). Where effects are observational (e.g., sexual violence under parenting), flag risk of bias and down-weight in PSA.**

**Response:** Thank you for this comment. We have strengthened the transparency of our effect-size synthesis and its external validity in both the Supplementary Materials and the main manuscript.

In the Supplementary Materials (S8 and S9 Appendices), we now provide a detailed description of the inclusion criteria used to select studies, the approach used to pool effect sizes across settings and follow-up time, and summary measures of between-site heterogeneity. This includes clarification of how estimates were combined and how variation across sites was assessed.

We have also expanded the Methods and Discussion sections to more clearly justify the application of education effects across sexes. Specifically, we now note that not all evaluations included in the meta-analysis were conducted among both sexes, which introduces some uncertainty. However, the evidence base includes both girl-focused and mixed-sex programmes, including Kenya’s CT-OVC evaluation, which spans a wide range of counties (Kisumu, Migori, Homa Bay, Suba, Nairobi, Garissa, and Kwale). Together with AGI-K evidence from Wajir and Nairobi, this provides an evidence base that is both geographically diverse and inclusive of mixed-sex samples across urban, rural, and pastoralist contexts. In addition, systematic reviews indicate no consistent gender differences in the educational effects of cash transfers, supporting the application of these effects across sexes.

We have also clarified the treatment of uncertainty across model components. Probabilistic sensitivity analysis (PSA) is applied to parameters driving long-term economic outcomes, including labour market assumptions (returns to schooling, wage growth, discount rates) and programme effectiveness through uncertainty in schooling gains. For human development outcomes, uncertainty is represented using bounded high and low effect estimates derived from the underlying evaluations, rather than being resampled within the PSA. For sexual violence, the effectiveness estimate is derived from observational evidence, and we explicitly acknowledge the greater uncertainty associated with this parameter. This is also reflected in wider confidence intervals around this estimate, as shown in Table 1, compared with those derived from experimental studies. We now also highlight this distinction in the Table 1 legend.

***Discussion:*** *“Additionally, not all evaluations included in the meta-analysis informing education impact parameters were conducted among both sexes. We therefore assumed similar effects for boys and girls. While this may introduce some uncertainty, the broader evidence base, including both girl-focused and mixed-sex programmes, indicates comparable educational impacts for boys and girls (22,102–104). A further strength of the meta-analysis is that it draws on evidence from a wide range of locations within Kenya for cash ‘plus’ interventions, as well as from studies in Tanzania and South Africa for parenting components, increasing confidence in the applicability of estimated effects across diverse settings (40,105).”*

**3. Time dynamics and decay assumptions: The authors assume impacts persist for three years post-exit (education, pregnancy/marriage) and a 33% annual decay for violence. Justify with evidence and show sensitivity to shorter/larger decay (0% to 50%)-these priors heavily influence a four-year horizon and any carry-forward effects.**

**Response:** Thank you for this important point. The original manuscript assumed impacts persisted for three years post-exit; we agree this was stronger than is directly supported by the available evidence and have revised our approach accordingly.

The AGI-K evaluation provides the most relevant longitudinal evidence on the persistence of impacts. It shows sustained effects at approximately two years post-exit (48 months post-baseline), with stronger and more consistent persistence observed for education outcomes across both Nairobi and Wajir. In contrast, impacts on adolescent pregnancy and child marriage are smaller, less precisely estimated, and more context-specific, with clearer effects primarily observed in the more disadvantaged Wajir setting. As shown in S8 Appendix, education effects remain relatively stable over time, whereas fertility-related outcomes attenuate and are not consistently statistically significant. This pattern indicates that persistence is not well captured by a single uniform assumption. Schooling effects appear relatively durable, while fertility-related impacts are more context-specific and attenuated.

We have therefore revised the model to adopt a baseline persistence of two years post-exit, which is directly supported by the AGI-K evidence and further informed by findings from the CT-OVC evaluation, which show suggestive but not consistently statistically significant improvements in education outcomes at approximately 48 months post-baseline. Evidence for pregnancy and marriage outcomes in CT-OVC is similarly limited and heterogeneous, reinforcing the interpretation that these effects are weaker and more context-specific.

Given limited evidence to support different persistence periods by outcome, we apply a common persistence duration across outcomes for parsimony, while explicitly acknowledging differences in the strength and consistency of evidence.

To reflect uncertainty, we now conduct sensitivity analyses varying the duration of persistence from 0 to 4 years post-exit, with four years representing an upper-bound scenario informed by longer-term follow-up evidence from AGI-K (72-month follow-up), derived from a single study in the Wajir setting. This sensitivity analysis is reported in S10 Appendix.

These revisions ensure that the modelling of time dynamics and decay is more closely aligned with the available empirical evidence and that uncertainty is transparently explored.

***Methods:*** *“Post-exit effect persistence was modelled as intervention specific. For the combined conditional cash transfers, community conversations, and adolescent clubs intervention, effects on education, pregnancy, and marriage outcomes were assumed to persist for two years following programme exit (whether due to graduation or programme completion). This assumption is consistent with evidence of sustained impacts on education outcomes observed at 48 months post-baseline (approximately two years post-exit) in the AGI-K evaluation, and is broadly supported by evidence from the CT-OVC evaluation, which shows positive but not consistently statistically significant effects on education outcomes at similar follow-up periods (33,34,61).”*

***Methods:*** *“In addition, we explored structural uncertainty related to the persistence of effects from cash ‘plus’ programming by modelling alternative durations of effect sustainability, informed by evidence suggesting impacts are typically measured up to two years post-receipt, with some evidence of persistence up to four years (24,33–35,61,62,76).”*

***Results:*** *“Additional sensitivity analyses explored assumptions regarding the persistence of intervention effects over time. While much of the evidence base for cash transfer programmes reflects impacts measured up to approximately two years post-receipt, longer-term follow-up from AGI-K in Wajir suggests that some effects may persist for up to four years (35). Under alternative scenarios in which impacts attenuate more rapidly, estimated lifetime earnings returns were reduced but remained positive and exceeded programme costs. Conversely, assuming sustained effects over a longer duration increased projected returns. Overall, conclusions regarding the economic viability of the programme were robust to plausible variations in effect persistence, S10 Appendix.”*

**4. Costing scope and supply-side capacity: The authors account for added teacher salary costs to preserve pupil-teacher ratios (good), but quality constraints likely extend to classrooms, materials, and supervision. Model alternative “supply-constrained” scenarios (e.g., only 50-75% of added enrolment accommodated) and show how CERs/ROI move. Also clarify whether administrative/monitoring costs scale linearly with coverage.**

**Response:** We agree that broader supply-side constraints are important and could affect both the feasibility and effectiveness of programme scale-up.

In the current model, we partially account for supply-side pressures by incorporating additional teacher salary costs to maintain baseline pupil–teacher ratios under increased enrolment. However, we do not explicitly model wider system constraints or capacity limits. Modelling such scenarios would require additional data on system capacity and the relationship between crowding and learning outcomes, which to our knowledge, are not currently available in a form suitable for this analysis.

We have therefore added a limitation to the Discussion noting that unmodelled supply constraints could reduce the magnitude of projected impacts if expansion outpaces system capacity. We also highlight this as an important area for future research that could inform scenario analysis.

With respect to programme delivery costs, administrative costs were estimated at the sub-county level and assumed to scale proportionally across sub-counties in the base model; this is now clarified in the Methods*.*

***Discussion:*** *“Second, the model assumes that increases in school enrolment will not compromise education quality. Although we incorporated the additional costs required to maintain pupil-to-teacher ratios, other aspects of education quality, such as teacher training, instructional materials, and classroom dynamics, may still be affected by rapid expansion, potentially limiting learning outcomes.”*

***Methods:*** *“Administrative costs were estimated at the sub-county level and assumed to scale proportionally across sub-counties.”*

**5. Double-counting worries vs. “discounted CER”: The “discounted CER across outcomes” allocates cost shares proportional to outcome contributions. This is a choice, not a standard in CEA, and can be misread as a welfare weight. Please: (i) rename (e.g., “multi-outcome cost allocation”); (ii) define the weights formally; (iii) compare against conventional single-outcome ICERs and a parallel CBA (one metric) to avoid the appearance of double-counting benefits.**

**Response:** This is an important clarification. The “discounted CER across outcomes” does not imply welfare weighting or aggregation of benefits in a way that risks double-counting. Rather, it allocates total programme costs across distinct outcome domains in proportion to their modelled contribution.

We retain this terminology because it reflects a published methodological approach described in Grueso et al. (2024; doi: 10.1080/19439342.2025.2591638), where the discounted CER framework is formally defined and applied. Additional technical detail on the derivation and properties of this method is available in that paper.

We have revised the manuscript to more clearly define the allocation approach, specify how weights are constructed, and emphasise that these represent proportional cost shares rather than social welfare weights. We also present conventional single-outcome cost-effectiveness ratios alongside the multi-outcome allocation results, and report the cost–benefit analysis separately, to avoid any appearance of double-counting.

***Methods:*** *“We estimated traditional cost-effectiveness ratios (CERs) by dividing total programme costs by total outcome units achieved.*

*To address the multi-outcome nature of the intervention, we also applied a discounted CER allocation method outlined in Grueso et al. (2024), proportionally allocating programme costs across outcomes based on their contribution to total benefits (73). These proportional weights represent cost shares rather than welfare weights.*

*CERs were compared with published estimates from other education-focused programmes in Kenya (74). Because these comparator estimates were derived using different costing methods, perspectives, time horizons, and contexts, comparisons should be interpreted as indicative rather than directly equivalent.”*

***Results:*** *“Relative to published estimates from comparator interventions, the modelled programme appears more cost-effective than the Kenya Teacher Incentives Programme, the Kenya Paying for School Uniforms Programme, and the Kenya Girls Merit Scholarship Programme, but less cost-effective than AGI-K Testing at Scale programme in Wajir (CER of US$101 per year of schooling gained, US$ 9 after discounted cost allocation) (49,80,81).*

*Relative to published estimates, the modelled programme does not appear more cost-effective than comparator interventions in reducing adolescent pregnancy or child marriage, though comparisons should be interpreted cautiously given methodological differences. Notably, the programme targets both girls and boys, whereas several comparators focused on girls. No comparator programmes reported impacts on violence outcomes, limiting direct comparisons.”*

**6. ROI framing and labour-market realism: ROI uses Mincer returns (11%/year of schooling), baseline wages = 2/3 GDPpc, 2% real wage growth, 3% discounting, and equal returns by sex. Provide Kenya-specific sensitivity: (a) Mincer returns ±50%; (b) 0-2% real growth; (c) female returns potentially higher than male but realized earnings lower due to participation gaps; (d) unemployment/underemployment risk in slower job-creation paths. Present ROI tornado plots and probability of ROI>1.**

**Response:** Thank you for this suggestion. We have added Kenya-specific sensitivity analyses to the ROI framework, including variation in Mincer returns (±50%), alternative assumptions on real wage growth (0–2%), and scenarios reflecting differential returns by sex alongside lower realised earnings due to participation gaps. In addition, we now incorporate uncertainty in programme effectiveness through variation in the number of additional years of schooling generated. These results are now presented using tornado plots, and we report the probability that ROI exceeds one. Together, these analyses demonstrate the robustness of our findings to key sources of uncertainty. Relevant sections of the Methods, Results, and Supplementary Materials have been updated accordingly.

**7. Comparator set and dominance claims: The authors compare CERs to a small set of education interventions and to AGI-K at scale in Wajir. Please expand or clarify comparators (uniforms, scholarships, teacher incentives) and ensure outcome definitions, populations, time horizons, and price years are aligned. Where AGI-K confidence intervals cross zero (unbounded ICERs), explain how you treat dominance or extended dominance.**

**Response:** We acknowledge this point. This study was not designed as a formal head-to-head cost-effectiveness analysis across interventions, and we did not attempt to fully harmonise outcome definitions, time horizons, analytic perspectives, or price years across comparator studies. The comparator CERs were drawn from published sources to provide contextual benchmarks rather than to support strict dominance claims.

We have clarified this in the Methods and Results sections and have tempered language to avoid implying formal dominance or extended dominance. In particular, for AGI-K in Wajir, where confidence intervals cross zero and ICERs may be unbounded, we now report point estimates with appropriate caution and do not make definitive efficiency rankings.

The comparator CERs are presented as contextual benchmarks rather than directly harmonised estimates. To improve transparency, we have added a column indicating the follow-up period for each comparator study, along with a detailed table note stating: *“Follow-up periods vary across comparator studies and are not directly comparable to the modelled lifetime horizon used for the cash ‘plus’ programme; comparisons should therefore be interpreted as illustrative.”* We further clarify that differences in outcome definitions, follow-up, populations, and costing methods mean that no formal dominance or extended dominance claims should be inferred.

***Discussion:*** *“In addition to estimating human development impacts and economic returns, we also estimated the cost-effectiveness of the programme relative to comparator programmes focused on adolescent education outcomes in Kenya. The modelled programme demonstrated favourable cost-effectiveness in improving educational outcomes, with cost-effectiveness broadly comparable to school-based interventions such as uniforms and merit scholarships. For adolescent pregnancy and child marriage, the programme appeared less cost-effective than some comparator interventions. However, these comparisons should be interpreted cautiously given differences in study populations, follow-up periods, outcome definitions, and analytical approaches. Higher cost-effectiveness ratios for these outcomes likely reflect the planned programme’s focus on both girls and boys, whereas some of the comparator interventions targeted only girls. Additionally, the lower cost-effectiveness on child marriage may reflect our national-level modelling, which includes areas with lower baseline prevalence than Wajir, the high-prevalence setting of the AGI-K programme (49).”*

**8. Targeting & equity analysis: The program targets NSNP households in 18 sub-counties. Provide equity-sensitive results: by sex, wealth quintile, county, rural/urban, initial out-of-school, and baseline prevalence (pregnancy/marriage/violence). Report concentration indices or simple distributional cost-effectiveness slices. Decision-makers will want to see who benefits most per shilling.**

**Response:** This is an important suggestion. We agree that equity-sensitive and distributional cost-effectiveness analyses would provide valuable insight into who benefits most per shilling invested.

However, conducting subgroup-specific modelling by age, geography, baseline schooling status, or other indicators of vulnerability would require substantially more granular data and structural extensions to the current model. This would constitute a new analysis rather than a minor revision.

We have added a paragraph in the Discussion acknowledging this limitation and highlighting equity-focused modelling as a priority area for future work.

***Discussion:*** *“Finally, while the programme is pro-poor by design, more granular data and model extensions would enable further disaggregation of impacts by key characteristics (e.g. age, geography, baseline schooling status) for distributional cost-effectiveness analysis. This represents a key area for future work.”*

**9. Overlaps and interactions across components: Most effect sizes come from bundled “cash plus” packages (cash + clubs + dialogues). Parenting is modelled separately. Consider interaction terms (super-additivity or crowd-out) and an ablation analysis (which component drives which outcome at the margin?). This helps with scale-design (what to keep if budgets tighten).**

**Response:** We agree that understanding complementarities across components would be valuable for programme design. However, the studies included in this review do not provide the variation needed to assess how individual components contribute to overall effects or how they interact. Most evaluations randomise bundled cash ‘plus’ packages against a control group rather than independently varying components, and parenting interventions are typically evaluated as stand-alone programmes rather than factorial designs. As a result, there is no credible evidence in the underlying studies to estimate super-additivity, crowd-out, or marginal component effects. We have therefore refrained from modelling interaction terms or attributing outcomes to specific components, as doing so would go beyond what the data can support. We now clarify this limitation in the text and note that future studies using factorial or multi-arm designs would be required to answer these questions rigorously.

**10. Violence outcomes measurement and ethics: Specify exact VACS/KDHS constructs, time frames, and baseline rates used; clarify whether modelled reductions reflect perpetration-specific pathways (e.g., caregiver vs. peer/partner) and how reporting bias is handled. Given the sensitivity of violence case management, justify excluding its effect from the base model but including its costs (or vice versa).**

**Response:** Thank you for this important comment. We have clarified the measurement of violence outcomes by specifying the exact VACS/KDHS constructs, reference periods, and baseline parameters used in the model.

Specifically, KDHS 2022 is used to parameterise age-specific incidence rates for adolescent pregnancy and child marriage, derived from reported age at first marriage and DHS calendar-based identification of pregnancy onset. These inputs are now explicitly described and reported in the manuscript and supplementary materials. Violence outcomes are based on past 12-month prevalence measures. Physical and emotional violence refer to violence perpetrated by parents, adult caregivers, or other adult relatives, and sexual violence includes unwanted sexual touching, unwanted attempted sex, physically forced sex, and sex pressured through harassment or threats, perpetrated by any individual. These estimates are drawn from VACS 2019 (national level).

We further clarify that modelled reductions are applied to caregiver-perpetrated violence pathways (emotional and physical violence), consistent with the intervention’s mechanism of action. No equivalent effect is assumed for violence perpetrated by non-caregivers, and sexual violence is modelled separately given the broader perpetrator definition.

Regarding reporting bias, we acknowledge that violence estimates are based on self-report and are likely subject to underreporting, particularly for sexual violence. In the absence of robust correction factors, we use observed prevalence as conservative baseline inputs and note this as a limitation in the interpretation of results.

We also now explain whether modelled reductions operate through caregiver- versus peer/partner-related pathways, and how potential reporting bias is considered in the interpretation of results.

In addition, we have clarified the treatment of violence case management in the model, including the rationale for excluding its effects from the base-case outcomes while retaining associated costs, given ethical and evidentiary considerations. These clarifications have been added to the Methods.

***Methods:*** *“The effects of violence case management were not modelled due to insufficient evidence on its effectiveness for reducing violence incidence. However, costs were included to reflect the resources required for ethical programme implementation.”*

***Discussion:*** “*Outcomes based on self-reported measures, particularly sensitive experiences such as sexual violence and child marriage, may be subject to reporting bias and are therefore likely to represent conservative estimates of the true underlying burden.”*

**11. Uncertainty communication: The authors present low/high based on 95% CIs of pooled effects. Add a proper probabilistic sensitivity analysis (1,000+ draws): show joint uncertainty on key outcomes, cost, ICERs, and ROI; give decision-useful statements (e.g., probability that “cash-plus” is more cost-effective than uniform/scholarship at thresholds X).**

**Response:** We agree that joint uncertainty is important for decision-making. We have therefore expanded the analysis to include a probabilistic sensitivity analysis (PSA) with 20,000 Monte Carlo draws, jointly propagating uncertainty in key parameters governing monetised benefits and economic returns. These include labour market assumptions (returns to schooling, wage growth, discount rates), as well as programme effectiveness through uncertainty in schooling gains.

For each draw, we compute lifetime benefit streams and return-on-investment (ROI), generating full distributions rather than relying on univariate confidence intervals. We now report uncertainty intervals derived from these distributions and present decision-relevant summaries, including the probability that benefits exceed programme costs and the probability of exceeding twice programme costs.

**12. Implementation realism & timeline: The model assumes immediate 2026 scale-up via existing registries and attendance-conditionality monitoring by teachers. Provide an implementation risk analysis: (i) registry accuracy and grievance redress; (ii) conditionality verification costs/compliance; (iii) payment rails; (iv) potential distortions (e.g., dropouts due to pregnancy losing transfers) and mitigation (grace periods, re-entry).**

**Response:** Thank you for this important point. The model assumes implementation beginning in 2026 through existing administrative systems, including social registries and school-based monitoring mechanisms. We agree that operational factors such as registry accuracy and grievance redress, conditionality verification, payment systems, and potential unintended behavioural responses are important for real-world implementation.

While a full implementation risk analysis is beyond the scope of the current modelling study, we have added a dedicated subsection in the Discussion to address these considerations explicitly. From a modelling perspective, these factors are expected to influence the timing and magnitude of impacts rather than the underlying direction of effects. For example, phased rollout or implementation delays would shift benefits over time and reduce net present value due to discounting, without fundamentally changing the overall conclusions under plausible scenarios. This is also because the programme scale is moderate relative to existing social protection delivery systems. We have clarified this in the revised Discussion.

***Discussion:*** *“The model assumes rollout through existing administrative systems, including social registries and school-based attendance monitoring. The programme scale is moderate relative to existing social protection systems and is implemented over a four-year period, supporting the feasibility of delivery through these channels. In practice, effectiveness will depend on registry accuracy and grievance redress mechanisms to minimise inclusion and exclusion errors. Conditionality verification may impose administrative burdens on schools, and payment delivery systems could face logistical delays, particularly in remote areas. Depending on their severity, these factors could attenuate overall impacts and returns.”*

**13. Climate and shock sensitivity: Kenya faces climate and macro shocks that could shift enrolment, fertility, and violence. Include scenario analysis for drought/flood years (attendance falls, households’ marginal utility of cash rises), and for macro downturns (lower wage growth), given that the authors explicitly noted these risks.**

**Response:** Thank you for this comment. We agree that climate and macroeconomic shocks are important factors that can affect enrolment, fertility, violence, and labour-market outcomes in Kenya. Explicitly modelling these dynamics would require additional assumptions and data beyond the scope of the current analysis. We have therefore not included scenario analyses for climate or macroeconomic shocks in this paper. We have, however, added a sentence to the Limitations section noting these risks and clarifying that we are actively developing methods to incorporate climate and shock sensitivity into future modelling work.

***Discussion:*** *“In addition, broader socio-economic and environmental changes, including climate-related shocks, may influence both programme impacts and economic returns over time. Better understanding and incorporating these risks in future analyses is also important.”*

**1. Perspective: The authors use a provider perspective for costs and beneficiary perspective for outcomes. They should clarify the welfare perspective when presenting ROI (societal? producer surplus?).**

**Response:** We thank the reviewer for this clarification request. Costs are measured from a provider (implementer) perspective, while benefits are measured at the beneficiary level. When presenting ROI, we therefore adopt a partial societal welfare perspective, in which programme costs represent real resource use and outcomes are valued as gains to beneficiaries. The ROI should be interpreted as the ratio of monetised beneficiary benefits to programme costs, rather than a full social surplus measure that would additionally account for fiscal externalities, general equilibrium effects, or distributional weights. We now clarify this interpretation explicitly in the methods and ROI discussion.

***Methods:*** *“Costs were estimated from a provider perspective, while outcomes and productivity gains were assessed from a beneficiary perspective. Return on investment (ROI) is therefore interpreted as the ratio of monetised beneficiary benefits to programme costs, reflecting a partial societal perspective that does not capture broader social welfare effects such as fiscal externalities, general equilibrium impacts, or distributional considerations.”*

**2. Outcome definitions: Adolescent pregnancy (<20) vs. program age (10-18), explain alignment in the Lexis setup.**

**Response:** Thank you for this comment. Although programme eligibility is ages 10–18, adolescent pregnancy is defined as occurring before age 20. We align these by using an age-structured Lexis framework in which cohorts enter the model at ages 10–18 and are followed over time as they age up to 20 for adolescent pregnancy and 18 for child marriage. This allows programme exposure during eligibility ages to influence risk at later ages (particularly 15–19), consistent with standard demographic definitions of adolescent pregnancy. We have clarified this alignment in the methodological appendices by explicitly describing cohort entry, ageing, and follow-up across the full risk period.

*“Health and protection outcomes (adolescent pregnancy, child marriage, and violence victimisation) were modelled using age-structured cohort models based on Lexis modelling principles (57). These models simulate multiple age cohorts advancing in annual time steps, with each cohort ageing one year per cycle. Cohorts enter at programme-eligible ages (10–18 years) and are followed over time as they age through the relevant risk periods (e.g., up to age 20 for adolescent pregnancy). Incidence of pregnancy, marriage, and violence was determined by age-specific incidence rates (58).”*

**3. Price year & exchange rate: Good practice shown; add a table of unit costs with ranges and sources for replication.**

**Response:** Thank you for this suggestion. We have added a table of unit costs, including ranges and data sources, to support transparency and replication, available via the Open Science Framework (OSF) repository, and updated the manuscript text accordingly.

***Methods:*** *“A full table of unit costs, including ranges and data sources, is available via the OSF repository (59).”*

**4. Terminology: Replace “discounted CER across outcomes” with a less standard-sounding label, as above.**

**Response:** Thank you for this suggestion. We recognise that the term “discounted CER across outcomes” may be unfamiliar and could be interpreted as implying welfare weighting. However, we retain this terminology because it reflects a published methodological approach described in Grueso et al. (2024), where the discounted CER framework is formally defined and applied.

To improve clarity, we have revised the manuscript to explicitly define this approach, clarify that it represents a proportional cost allocation across outcomes rather than a welfare-weighted aggregation, and distinguish it from conventional single-outcome cost-effectiveness ratios. We also present standard single-outcome CERs and report the cost–benefit analysis separately to avoid any potential misinterpretation.

**5. CHEERS checklist: Include as SI; several required items (model structure diagram with modules, PSA methods, validation) should be cross-referenced.**

**Response:** Thank you for this suggestion. We have included the CHEERS checklist as Supplementary Information and cross-referenced the relevant items in the manuscript.
