# Supplementary material for: Estimating the human development impacts and economic returns from an adolescent cash ‘plus’ programme in Kenya: An economic modelling study
Source: PLOS Glob Public Health. 2026 Jul 24;6(7):e0006822. doi: 10.1371/journal.pgph.0006822 (PMC13399278; doi:10.1371/journal.pgph.0006822)
Supplement: Attachment — Submitted filename: Response_to_reviewers_comments_auresp_2.docx [file pgph.0006822.s004.docx]

**Reviewer #3:**

**You state you used articles from outside Kenya - South Africa and Tanzania- is it correct in assuming you used those in the modelling? These are different country contexts and should not be used to extrapolate for Kenya.**

**Response:** Thank you for this important comment. Our approach was to prioritise evidence sources according to their contextual relevance and availability. Where available, we used Kenya-specific data. In cases where Kenyan evidence was unavailable, we sought evidence from neighbouring or regionally comparable settings before considering broader regional estimates, global estimates, or assumptions.

The studies from South Africa and Tanzania were therefore used only where relevant Kenyan data were not available and were selected because they represent sub-Saharan African contexts that are likely to be more comparable to Kenya than evidence drawn from global sources or unsupported assumptions. We agree that contextual differences between countries should be recognised and have clarified in the manuscript which parameters were informed by evidence from outside Kenya. We have also highlighted this as a limitation and an area where future Kenya-specific data would strengthen model estimates.

***Methods:*** *“Effectiveness estimates were identified through targeted literature reviews and expert consultation (Table 1, S8 Appendix, and S9 Appendix). Where available, Kenya-specific evidence was used to parameterise intervention effects; when such evidence was unavailable, we prioritised evidence from geographically and contextually similar sub-Saharan African settings before considering broader regional evidence or assumptions.”*

***Methods:*** *“Parenting support effects on emotional and physical violence victimisation were derived from randomised controlled trials of the Sinovuyo Teen and Furaha Teen interventions conducted in South Africa and Tanzania, respectively, as no comparable Kenyan trial evidence was available at the time of modelling (52,63).”*

***Discussion:*** *“Although Kenya-specific evidence was prioritised wherever possible, some effectiveness parameters were informed by studies from Tanzania and South Africa where equivalent Kenyan evidence was unavailable (40,106). These studies were selected because they were conducted in sub-Saharan African settings that are likely to be more comparable to Kenya than broader regional or global evidence. Nevertheless, differences in programme implementation, social norms, and economic context may affect the transferability of effect estimates, and future analyses would benefit from additional Kenya-specific evidence.”*

**Given the importance of the study- what is the next step for county governments - can you discuss a bit about budgeting for these interventions? Is this feasible for the government to support? What are the programmatic implications -- you state stakeholders will benefit as will policies - but unsure how this fits in the Kenya context of devolution.**

**Response:** Thank you for this valuable suggestion. We agree that the implications of these findings within Kenya's devolved governance system warrant further discussion. We have expanded the Discussion section to better articulate how county governments could utilise this evidence in planning and budgeting processes.

Our findings suggest that adolescent cash-plus interventions may represent a worthwhile investment given their potential benefits across multiple sectors, including education, health, social protection, and gender-based violence prevention. However, implementation at scale would require consideration of fiscal constraints, inter-sectoral coordination, and the respective responsibilities of national and county governments under devolution

We have therefore expanded the discussion to highlight both the opportunities and challenges associated with financing and implementing these interventions within county systems. In particular, we note that further work is needed to understand sustainable financing mechanisms, the distribution of costs and benefits across levels of government, and how adolescent-focused interventions can be integrated into existing county development and social protection priorities.

***Discussion:*** *“As adolescent cash-plus programming is being scaled through the State Department for Children’s Services, these results suggest that county governments may benefit from incorporating adolescent wellbeing objectives into joint planning and budgeting processes across social protection, health, education, and child protection sectors, reflecting the broad distribution of programme benefits.”*

**Reviewer #4:**

**This manuscript does a very good job demonstrating the adolescent cash "plus" program in Kenya has significant impact in terms of additional years of schooling, averting adolescent pregnancies, child marriage and sexual violence. In addition the estimates for job earnings and cost-effectiveness is commendable. This work is a good reminder of for programmers across boundaries to pay attention to implement similar schemes to bring changes in the lives of adolescents.
The authors have also clarified and addressed several of the questions raised by previous reviewers.**

**Response:** We thank the reviewer for their positive assessment of our manuscript and for recognising the contribution of this work to the evidence base on adolescent cash-plus programmes. We appreciate the reviewer's acknowledgement of the revisions made in response to previous comments. The constructive feedback from all reviewers has helped strengthen the manuscript, and we are grateful for the opportunity to improve the clarity and policy relevance of our study.
