# Supplementary material for: Estimating the human development impacts and economic returns from an adolescent cash ‘plus’ programme in Kenya: An economic modelling study
Source: PLOS Glob Public Health. 2026 Jul 24;6(7):e0006822. doi: 10.1371/journal.pgph.0006822 (PMC13399278; doi:10.1371/journal.pgph.0006822)
Supplement: Peer review history

# Author comment: Author response to Decision Letter 0

**DOI:** 10.1371/journal.pgph.0006822.r001

**Transfer Alert**

This paper was transferred from another journal. As a result, its full editorial history (including decision letters, peer reviews and author responses) may not be present.

24 Aug 2025

# Peer review: Decision Letter 0

**DOI:** 10.1371/journal.pgph.0006822.r002

**Authors:** Dickson Amugsi

5 Oct 2025

PGPH-D-25-02429

Estimating the human development impacts and economic returns from an adolescent cash ‘plus’ programme in Kenya: An economic modelling study

PLOS Global Public Health

Dear Dr. Rudgard,

Thank you for submitting your manuscript to PLOS Global Public Health. After careful consideration, we feel that it has merit but does not fully meet PLOS Global Public Health’s publication criteria as it currently stands. Therefore, we invite you to submit a revised version of the manuscript that addresses the points raised during the review process.

EDITOR: Please insert comments here and delete this placeholder text when finished. Be sure to:

- Please ensure that your decision is justified on PLOS Global Public Health’s publication criteria and not, for example, on novelty or perceived impact.
  Please submit your revised manuscript by 25/10/2025. If you will need more time than this to complete your revisions, please reply to this message or contact the journal office at globalpubhealth@plos.org. When you're ready to submit your revision, log on to https://www.editorialmanager.com/pgph/ and select the 'Submissions Needing Revision' folder to locate your manuscript file.
  Please include the following items when submitting your revised manuscript:

- A rebuttal letter that responds to each point raised by the editor and reviewer(s). You should upload this letter as a separate file labeled 'Response to Reviewers'.
- A marked-up copy of your manuscript that highlights changes made to the original version. You should upload this as a separate file labeled 'Revised Manuscript with Track Changes'.
- An unmarked version of your revised paper without tracked changes. You should upload this as a separate file labeled 'Manuscript'.

Guidelines for resubmitting your figure files are available below the reviewer comments at the end of this letter.

We look forward to receiving your revised manuscript.

Kind regards,

Dickson Abanimi Amugsi, PhD

Academic Editor

PLOS Global Public Health

Journal Requirements:

1. Please note that PLOS Global Public Health has specific guidelines on code sharing for submissions in which author-generated code underpins the findings in the manuscript. In these cases, all author-generated code must be made available without restrictions upon publication of the work. Please review our guidelines at https://journals.plos.org/globalpublichealth/s/materials-and-software-sharing#loc-sharing-code and ensure that your code is shared in a way that follows best practice and facilitates reproducibility and reuse.

If the reviewer comments include a recommendation to cite specific previously published works, please review and evaluate these publications to determine whether they are relevant and should be cited. There is no requirement to cite these works unless the editor has indicated otherwise.

Additional Editor Comments (if provided):

[Note: HTML markup is below. Please do not edit.]

Reviewers' comments:

Reviewer's Responses to Questions

**Comments to the Author**

1. Does this manuscript meet PLOS Global Public Health’s publication criteria? Is the manuscript technically sound, and do the data support the conclusions? The manuscript must describe methodologically and ethically rigorous research with conclusions that are appropriately drawn based on the data presented.

Reviewer #1: Yes

Reviewer #2: Partly

**********

2. Has the statistical analysis been performed appropriately and rigorously?

Reviewer #1: Yes

Reviewer #2: Yes

**********

3. Have the authors made all data underlying the findings in their manuscript fully available (please refer to the Data Availability Statement at the start of the manuscript PDF file)?

The PLOS Data policy requires authors to make all data underlying the findings described in their manuscript fully available without restriction, with rare exception. The data should be provided as part of the manuscript or its supporting information, or deposited to a public repository. For example, in addition to summary statistics, the data points behind means, medians and variance measures should be available. If there are restrictions on publicly sharing data—e.g. participant privacy or use of data from a third party—those must be specified.

Reviewer #1: Yes

Reviewer #2: No

**********

4. Is the manuscript presented in an intelligible fashion and written in standard English?

PLOS Global Public Health does not copyedit accepted manuscripts, so the language in submitted articles must be clear, correct, and unambiguous. Any typographical or grammatical errors should be corrected at revision, so please note any specific errors here.

Reviewer #1: Yes

Reviewer #2: Yes

**********

5. Review Comments to the Author

Please use the space provided to explain your answers to the questions above. You may also include additional comments for the author, including concerns about dual publication, research ethics, or publication ethics. (Please upload your review as an attachment if it exceeds 20,000 characters)

Reviewer #1: Review of review of “Estimating the human development impacts and economic returns from an adolescent cash ‘plus’ programme in Kenya: An economic modelling study” submitted to PLOS Global Public Health (PGPH-D-25-2492). The article, presumably based on on a comprehensive report or feasibility study, represents a thorough example of approximating the economic impacts of scaling up a cash transfer plus program in Kenya. Impact studies alone too often provide only estimated effects on a selected set of important outcomes, occasionally presenting cost-effectiveness but only rarely providing insights into actual economic returns or cost-benefit (with benefits measured here as the returns to increased human capital in the labor market). As an example of the latter, this study can serve as a model guide for other similar exercises. Important in its approach is indicating some sensitivity around the mid-case estimates, as there are many underlying assumptions. The concluding discussion makes clear some of the potential weaknesses, including possible understatement of benefits suggesting findings might be conservative.

COMMENTS

1. p. 2 – The tables containing program impacts do not strongly suggest to me that the cash plus is greater than cash for this Kenyan program, but I may be missing something (Table 2 in supplementary materials).

2. p. 3 I was unfamiliar with the term “decision analytic-economic modelling” as a specific approach, and suggest it be defined (sorry if I missed this).

3. p. 3 full range of potential outcomes – yes, though in practice and in this study that is not feasible. Perhaps a fuller range?

4. p. 3 Indicates plans to implement the program in 2025. Since it is now late in the year, has this been done or dates modified?

5. p. 6 – A provider perspective for costs and a beneficiary perspective for outcomes/benefits seems appropriate. It was nice to see that additional teacher costs, for example, were taken into consideration. But this approach does exclude private costs which for some could be important (and may also be heterogeneous).

6. How sensitive are the findings to alternative discount rates, if some were considered?

7. I did not notice explicit discussion of the return to education in the labor market results, but possibly missed them in the appendix? If not, a brief description of that may be useful, 40 years is a long time period.

Reviewer #2: Review report on manuscript: Estimating the human development impacts and economic returns from an adolescent cash ‘plus’ programme in Kenya: An economic modelling study

Summary

The manuscript models the human-development impacts, economic returns, and cost-effectiveness of an adolescent cash “plus” package in nine Kenyan counties (2026-2029). Components include conditional cash transfers (CCTs) tied to school attendance, adolescent clubs, community conversations, parenting support (PLH-Teen), and case management (modelled qualitatively). The model projects sizeable gains in years of schooling and reductions in adolescent pregnancy, child marriage, and violence, at ~US$42.5m over four years, with an ROI of ~US$246m in future labour productivity and favourable CERs relative to several education comparators.

Overall, the paper addresses a timely policy question with an ambitious cross-sector modelling framework. However, in its current form the economic methods, parameter provenance, and sensitivity analysis communication need revising before the paper gets published and results inform policy decisions.

Major comments

1. Transparency & reproducibility of the model

The study rests on multiple linked Excel models (education transition; Lexis age-cohort models; earnings projections). Please release the full code/model files and a runbook (inputs → transformations → outputs), plus a parameter dictionary mapping each input to a source and a baseline value/CI. PLOS requires open data/code “sufficient to reproduce the findings”; the current statement (“all relevant data are within the paper/SI”) is not enough for a decision-analytic model.

2. Effect‐size synthesis and external validity

Several effects are pulled from AGI-K and other evaluations, with some meta-analytic pooling across Nairobi and Wajir and carry-over to mixed-sex beneficiaries. That’s defensible but show: (i) inclusion criteria; (ii) pooling method; (iii) between-site heterogeneity; (iv) why effects for girls can be applied to boys (or provide sex-specific sensitivity bounds). Where effects are observational (e.g., sexual violence under parenting), flag risk of bias and down-weight in PSA.

3. Time dynamics and decay assumptions

The authors assume impacts persist for three years post-exit (education, pregnancy/marriage) and a 33% annual decay for violence. Justify with evidence and show sensitivity to shorter/larger decay (0% to 50%)-these priors heavily influence a four-year horizon and any carry-forward effects.

4. Costing scope and supply-side capacity

The authors account for added teacher salary costs to preserve pupil-teacher ratios (good), but quality constraints likely extend to classrooms, materials, and supervision. Model alternative “supply-constrained” scenarios (e.g., only 50-75% of added enrolment accommodated) and show how CERs/ROI move. Also clarify whether administrative/monitoring costs scale linearly with coverage.

5. Double-counting worries vs. “discounted CER”

The “discounted CER across outcomes” allocates cost shares proportional to outcome contributions. This is a choice, not a standard in CEA, and can be misread as a welfare weight. Please: (i) rename (e.g., “multi-outcome cost allocation”); (ii) define the weights formally; (iii) compare against conventional single-outcome ICERs and a parallel CBA (one metric) to avoid the appearance of double-counting benefits.

6. ROI framing and labour-market realism

ROI uses Mincer returns (11%/year of schooling), baseline wages = 2/3 GDPpc, 2% real wage growth, 3% discounting, and equal returns by sex. Provide Kenya-specific sensitivity: (a) Mincer returns ±50%; (b) 0-2% real growth; (c) female returns potentially higher than male but realized earnings lower due to participation gaps; (d) unemployment/underemployment risk in slower job-creation paths. Present ROI tornado plots and probability of ROI>1.

7. Comparator set and dominance claims

The authors compare CERs to a small set of education interventions and to AGI-K at scale in Wajir. Please expand or clarify comparators (uniforms, scholarships, teacher incentives) and ensure outcome definitions, populations, time horizons, and price years are aligned. Where AGI-K confidence intervals cross zero (unbounded ICERs), explain how you treat dominance or extended dominance.

8. Targeting & equity analysis

The program targets NSNP households in 18 sub-counties. Provide equity-sensitive results: by sex, wealth quintile, county, rural/urban, initial out-of-school, and baseline prevalence (pregnancy/marriage/violence). Report concentration indices or simple distributional cost-effectiveness slices. Decision-makers will want to see who benefits most per shilling.

9. Overlaps and interactions across components

Most effect sizes come from bundled “cash plus” packages (cash + clubs + dialogues). Parenting is modelled separately. Consider interaction terms (super-additivity or crowd-out) and an ablation analysis (which component drives which outcome at the margin?). This helps with scale-design (what to keep if budgets tighten).

10. Violence outcomes: measurement and ethics

Specify exact VACS/KDHS constructs, time frames, and baseline rates used; clarify whether modelled reductions reflect perpetration-specific pathways (e.g., caregiver vs. peer/partner) and how reporting bias is handled. Given the sensitivity of violence case management, justify excluding its effect from the base model but including its costs (or vice versa).

11. Uncertainty communication

The authors present low/high based on 95% CIs of pooled effects. Add a proper probabilistic sensitivity analysis (1,000+ draws): show joint uncertainty on key outcomes, cost, ICERs, and ROI; give decision-useful statements (e.g., probability that “cash-plus” is more cost-effective than uniform/scholarship at thresholds X).

12. Implementation realism & timeline

The model assumes immediate 2026 scale-up via existing registries and attendance-conditionality monitoring by teachers. Provide an implementation risk analysis: (i) registry accuracy and grievance redress; (ii) conditionality verification costs/compliance; (iii) payment rails; (iv) potential distortions (e.g., dropouts due to pregnancy losing transfers) and mitigation (grace periods, re-entry).

13. Climate and shock sensitivity

Kenya faces climate and macro shocks that could shift enrolment, fertility, and violence. Include scenario analysis for drought/flood years (attendance falls, households’ marginal utility of cash rises), and for macro downturns (lower wage growth), given that the authors explicitly noted these risks.

Minor comments (editorial/clarificatory)

1. Perspective: The authors use a provider perspective for costs and beneficiary perspective for outcomes. They should clarify the welfare perspective when presenting ROI (societal? producer surplus?).

2. Outcome definitions: Adolescent pregnancy (<20) vs. program age (10-18), explain alignment in the Lexis setup.

3. Price year & exchange rate: Good practice shown; add a table of unit costs with ranges and sources for replication.

4. Terminology: Replace “discounted CER across outcomes” with a less standard-sounding label, as above.

5. CHEERS checklist: Include as SI; several required items (model structure diagram with modules, PSA methods, validation) should be cross-referenced.

**********

6. PLOS authors have the option to publish the peer review history of their article (what does this mean?). If published, this will include your full peer review and any attached files.

**Do you want your identity to be public for this peer review?**  If you choose “no”, your identity will remain anonymous but your review may still be made public.

For information about this choice, including consent withdrawal, please see our Privacy Policy.

Reviewer #1: No

Reviewer #2: No

**********

[NOTE: If reviewer comments were submitted as an attachment file, they will be attached to this email and accessible via the submission site. Please log into your account, locate the manuscript record, and check for the action link "View Attachments". If this link does not appear, there are no attachment files.]

-->Figure Resubmissions:

While revising your submission, we strongly recommend that you use PLOS’s NAAS tool (https://ngplosjournals.pagemajik.ai/artanalysis) to test your figure files. NAAS can convert your figure files to the TIFF file type and meet basic requirements (such as print size, resolution), or provide you with a report on issues that do not meet our requirements and that NAAS cannot fix.

After uploading your figures to PLOS’s NAAS tool - https://ngplosjournals.pagemajik.ai/artanalysis, NAAS will process the files provided and display the results in the "Uploaded Files" section of the page as the processing is complete. If the uploaded figures meet our requirements (or NAAS is able to fix the files to meet our requirements), the figure will be marked as "fixed" above. If NAAS is unable to fix the files, a red "failed" label will appear above. When NAAS has confirmed that the figure files meet our requirements, please download the file via the download option, and include these NAAS processed figure files when submitting your revised manuscript.

# Author comment: Author response to Decision Letter 1

**DOI:** 10.1371/journal.pgph.0006822.r003

24 Mar 2026

Attachment Submitted filename: Response to reviewers comments.docx

# Peer review: Decision Letter 1

**DOI:** 10.1371/journal.pgph.0006822.r004

**Authors:** Dickson Amugsi

25 May 2026

PGPH-D-25-02429R1

Estimating the human development impacts and economic returns from an adolescent cash ‘plus’ programme in Kenya: An economic modelling study

PLOS Global Public Health

Dear Dr. Rudgard,

Thank you for submitting your manuscript to PLOS Global Public Health. After careful consideration, we feel that it has merit but does not fully meet PLOS Global Public Health’s publication criteria as it currently stands. Therefore, we invite you to submit a revised version of the manuscript that addresses the points raised during the review process.

Please submit your revised manuscript by 06/06/2026. If you will need more time than this to complete your revisions, please reply to this message or contact the journal office at globalpubhealth@plos.org. When you're ready to submit your revision, log on to https://www.editorialmanager.com/pgph/ and select the 'Submissions Needing Revision' folder to locate your manuscript file.

Please include the following items when submitting your revised manuscript:

- A letter that responds to each point raised by the editor and reviewer(s). You should upload this letter as a separate file labeled 'Response to Reviewers'.
- A marked-up copy of your manuscript that highlights changes made to the original version. You should upload this as a separate file labeled 'Revised Manuscript with Track Changes'.
- An unmarked version of your revised paper without tracked changes. You should upload this as a separate file labeled 'Manuscript'.

Guidelines for resubmitting your figure files are available below the reviewer comments at the end of this letter.

As the corresponding author, your ORCID iD is verified in the submission system and will appear in the published article. PLOS supports the use of ORCID, and we encourage all coauthors to register for an ORCID iD and use it as well. Please encourage your coauthors to verify their ORCID iD within the submission system before final acceptance, as unverified ORCID iDs will not appear in the published article. *Only*  the individual author can complete the verification step; PLOS staff *cannot*  verify ORCID iDs on behalf of authors.

We look forward to receiving your revised manuscript.

Kind regards,

Dickson Abanimi Amugsi, PhD

Academic Editor

PLOS Global Public Health

Journal Requirements:

1. If the reviewer comments include a recommendation to cite specific previously published works, please review and evaluate these publications to determine whether they are relevant and should be cited. There is no requirement to cite these works unless the editor has indicated otherwise.

2. Please review your reference list to ensure that it is complete and correct. If you have cited papers that have been retracted, please include the rationale for doing so in the manuscript text, or remove these references and replace them with relevant current references. Any changes to the reference list should be mentioned in the rebuttal letter that accompanies your revised manuscript. If you need to cite a retracted article, indicate the article’s retracted status in the References list and also include a citation and full reference for the retraction notice.

Additional Editor Comments (if provided):

Thank you for submitting your manuscript for publication. While two reviewers (reviewers 1 and 4) recommended acceptance, reviewer 3 raised some minor issues that I invite you to address before making a final decision.

I look forward to receiving your revision soon. Thank you!

[Note: HTML markup is below. Please do not edit.]

Reviewers' comments:

Reviewer's Responses to Questions

**Comments to the Author**

1. If the authors have adequately addressed your comments raised in a previous round of review and you feel that this manuscript is now acceptable for publication, you may indicate that here to bypass the “Comments to the Author” section, enter your conflict of interest statement in the “Confidential to Editor” section, and submit your "Accept" recommendation.

Reviewer #1: All comments have been addressed

Reviewer #3: (No Response)

Reviewer #4: All comments have been addressed

**********

2. Does this manuscript meet PLOS Global Public Health’s publication criteria? Is the manuscript technically sound, and do the data support the conclusions? The manuscript must describe methodologically and ethically rigorous research with conclusions that are appropriately drawn based on the data presented.

Reviewer #1: Yes

Reviewer #3: Yes

Reviewer #4: Yes

**********

3. Has the statistical analysis been performed appropriately and rigorously?

Reviewer #1: Yes

Reviewer #3: I don't know

Reviewer #4: Yes

**********

4. Have the authors made all data underlying the findings in their manuscript fully available (please refer to the Data Availability Statement at the start of the manuscript PDF file)?

The PLOS Data policy requires authors to make all data underlying the findings described in their manuscript fully available without restriction, with rare exception. The data should be provided as part of the manuscript or its supporting information, or deposited to a public repository. For example, in addition to summary statistics, the data points behind means, medians and variance measures should be available. If there are restrictions on publicly sharing data—e.g. participant privacy or use of data from a third party—those must be specified.

Reviewer #1: Yes

Reviewer #3: Yes

Reviewer #4: Yes

**********

5. Is the manuscript presented in an intelligible fashion and written in standard English?

PLOS Global Public Health does not copyedit accepted manuscripts, so the language in submitted articles must be clear, correct, and unambiguous. Any typographical or grammatical errors should be corrected at revision, so please note any specific errors here.

Reviewer #1: Yes

Reviewer #3: Yes

Reviewer #4: Yes

**********

6. Review Comments to the Author

Please use the space provided to explain your answers to the questions above. You may also include additional comments for the author, including concerns about dual publication, research ethics, or publication ethics. (Please upload your review as an attachment if it exceeds 20,000 characters)

Reviewer #1: Thank you for addressing my and the other reviewer comments

Reviewer #3: The article was well written,

1. You state you used articles from outside Kenya - South Africa and Tanzania- is it correct in assuming you used those in the modelling? These are different country contexts and should not be used to extrapolate for Kenya.

2. Given the importance of the study- what is the next step for county governments - can you discuss a bit about budgeting for thse interventions? Is this feasible for the government to support? What are the programmatic implications -- you state stakeholders will benefit as will policies - but unsure how this fits in the Kenya context of devolution.

To our knowledge, this is the first study to assess the impact of scaling up a package

of evidence-based, policy-focused interventions on multiple outcomes across the

domains of education, health, violence, and labour. By moving beyond single cost–

benefit metrics and quantifying impacts across multiple outcome pathways, the study

provides a more comprehensive assessment of value across sectors. This is

particularly important for facilitating stakeholder engagement and policy development

in cross-sectoral programmes, where benefits may otherwise be difficult to compare

or attribute (91,92).

Reviewer #4: This manuscript does a very good job demonstrating the adolescent cash "plus" program in Kenya has significant impact in terms of additional years of schooling, averting adolescent pregnancies, child marriage and sexual violence. In addition the estimates for job earnings and cost-effectiveness is commendable. This work is a good reminder of for programmers across boundaries to pay attention to implement similar schemes to bring changes in the lives of adolescents.

The authors have also clarified and addressed several of the questions raised by previous reviewers.

**********

7. PLOS authors have the option to publish the peer review history of their article (what does this mean?). If published, this will include your full peer review and any attached files.

**Do you want your identity to be public for this peer review?**  If you choose “no”, your identity will remain anonymous but your review may still be made public.

For information about this choice, including consent withdrawal, please see our Privacy Policy.

Reviewer #1: No

Reviewer #3: No

Reviewer #4: No

**********

[NOTE: If reviewer comments were submitted as an attachment file, they will be attached to this email and accessible via the submission site. Please log into your account, locate the manuscript record, and check for the action link "View Attachments". If this link does not appear, there are no attachment files.]

Figure Resubmissions:

While revising your submission, we strongly recommend that you use PLOS’s NAAS tool (https://ngplosjournals.pagemajik.ai/artanalysis) to test your figure files. NAAS can convert your figure files to the TIFF file type and meet basic requirements (such as print size, resolution), or provide you with a report on issues that do not meet our requirements and that NAAS cannot fix.

After uploading your figures to PLOS’s NAAS tool - https://ngplosjournals.pagemajik.ai/artanalysis, NAAS will process the files provided and display the results in the "Uploaded Files" section of the page as the processing is complete. If the uploaded figures meet our requirements (or NAAS is able to fix the files to meet our requirements), the figure will be marked as "fixed" above. If NAAS is unable to fix the files, a red "failed" label will appear above. When NAAS has confirmed that the figure files meet our requirements, please download the file via the download option, and include these NAAS processed figure files when submitting your revised manuscript.

# Author comment: Author response to Decision Letter 2

**DOI:** 10.1371/journal.pgph.0006822.r005

18 Jun 2026

Attachment Submitted filename: Response_to_reviewers_comments_auresp_2.docx

# Editor report: Decision Letter 2

**DOI:** 10.1371/journal.pgph.0006822.r006

**Authors:** Dickson Amugsi

21 Jun 2026

Estimating the human development impacts and economic returns from an adolescent cash ‘plus’ programme in Kenya: An economic modelling study

PGPH-D-25-02429R2

Dear Dr Rudgard,

We are pleased to inform you that your manuscript 'Estimating the human development impacts and economic returns from an adolescent cash ‘plus’ programme in Kenya: An economic modelling study' has been provisionally accepted for publication in PLOS Global Public Health.

Before your manuscript can be formally accepted you will need to complete some formatting changes, which you will receive in a follow up email. A member of our team will be in touch with a set of requests.

Please note that your manuscript will not be scheduled for publication until you have made the required changes, so a swift response is appreciated.

IMPORTANT: The editorial review process is now complete. PLOS will only permit corrections to spelling, formatting or significant scientific errors from this point onwards. Requests for major changes, or any which affect the scientific understanding of your work, will cause delays to the publication date of your manuscript.

If your institution or institutions have a press office, please notify them about your upcoming paper to help maximize its impact. If they'll be preparing press materials, please inform our press team as soon as possible -- no later than 48 hours after receiving the formal acceptance. Your manuscript will remain under strict press embargo until 2 pm Eastern Time on the date of publication. For more information, please contact globalpubhealth@plos.org.

Thank you again for supporting Open Access publishing; we are looking forward to publishing your work in PLOS Global Public Health.

Best regards,

Dickson Abanimi Amugsi, PhD

Academic Editor

PLOS Global Public Health

***********************************************************

Reviewer Comments (if any, and for reference):
